# ScreenSeed as a novel high throughput seed germination phenotyping method

**DOI:** 10.1038/s41598-020-79115-2

**Published:** 2021-01-14

**Authors:** Nicolas Merieux, Pierre Cordier, Marie-Hélène Wagner, Sylvie Ducournau, Sophie Aligon, Dominique Job, Philippe Grappin, Edwin Grappin

**Affiliations:** 1 EffiSciency, ScreenSeed, Issy-les-Moulineaux, 97132 France; 2 Groupe d’Étude et de Contrôle des Variétés et des Semences (GEVES, Dept Seed Testing, Station Nationale d’Essais de Semences (SNES), 49071 Beaucouzé, France; 3 Institut de recherche en horticulture et semences (IRHS), UMR 1345 INRAE - Institut Agro - Université d’Angers, SFR 4207 QuaSav, 49071 Beaucouzé, France; 4 Microbiologie, Adaptation et Pathogénie, UMR 5240 CNRS - INSA - Université Claude Bernard Lyon1 - Bayer CropScience, 69009 Lyon, France

**Keywords:** Plant sciences, Plant biotechnology, Plant development, Plant genetics, Plant physiology, Plant stress responses

## Abstract

A high throughput phenotyping tool for seed germination, the ScreenSeed technology, was developed with the aim of screening genotype responsiveness and chemical drugs. This technology was presently used with *Arabidopsis thaliana* seeds to allow characterizing seed samples germination behavior by incubating seeds in 96-well microplates under defined conditions and detecting radicle protrusion through the seed coat by automated image analysis. This study shows that this technology provides a fast procedure allowing to handle thousands of seeds without compromising repeatability or accuracy of the germination measurements. Potential biases of the experimental protocol were assessed through statistical analyses of germination kinetics. Comparison of the ScreenSeed procedure with commonly used germination tests based upon visual scoring displayed very similar germination kinetics.

## Introduction

To face the challenge of climate and demographic changes a sustainable intensification of agricultural production is needed^1,2^. The discovery of new products (molecules and seed treatments) that stimulate crop growth and yield under environmental constraints^3^ and of new crop genotypes that are better adapted to such environmental stresses will be of paramount importance. Among several approaches, improving seed germination vigor is a strategic lever since it directly impacts crop yields^4^. Industrial development of seed technologies^5^ unraveled the usefulness of pre-germinative treatments including seed priming (controlled seed hydration followed by redrying), soaking or coating seeds with phytopharmaceuticals or various chemicals to improve seed vigor, seedling establishment and to limit pathogen transmission in the crop plants. The long-standing interest of plant biologists in seed germination allowed to evidence many aspects contributing to seed quality, such as desiccation tolerance, longevity, dormancy, vigor, and adaptability to biotic and abiotic stressed environments (for reviews, see^6–8^). In these studies the phenotypic criteria are generally expressed by kinetics of cumulative germination events during hydration time of seed samples and are often modeled by parametric probability distributions^9,10^. Thanks to the availability of genetic resources and the development of systems biology approaches for the *Arabidopsis thaliana* model species (herein referred to as Arabidopsis)^11^, germination physiology and seed response mechanisms to external stimuli have been widely described at the transcriptome, proteome and metabolome levels^12–20^ notably through the use of pharmacological and mutant approaches.

Phenotyping germination behavior in Arabidopsis has for a long time required the manual sowing of large numbers of seeds and the daily scoring of germinated seeds by visual monitoring using binocular magnifying glasses. Such studies have provided valuable insights on the genetic control of seed dormancy, seed longevity and seed tolerance to osmotic and temperature stresses^21–25^. Recently, the development of automated phenotyping methods allowed high-throughput screening of seed germination^26–30^. For example, a screening protocol used chlorophyll fluorescence-based imaging (ChIF) system to daily detect in Petri dishes the emerging cotyledons. This method has been successfully used to identify in Arabidopsis abscisic acid (ABA) resistant genotypes among candidate mutants affected in RING-type ubiquitin E3 ligase genes exhibiting ABA regulation^30^.

Also, three automated phenotyping systems using RGB image-based analysis of radicle emergence^26,27,31^ were shown to be efficient to monitor seed germination kinetics and to deliver useful germination-related metrics^32^, such as $t_{50}$ (time to reach 50% of seed germination), $U_{80-20}$ (time spread of germination expressed as the time interval between 20% and 80% germination) and $G_{max}$ (maximal germination percentage). This allowed comparing the seed germination characteristics of numerous plant genotypes^33–35^. The first one is a computer vision technology developed for monitoring germination from seeds sown in Jacobsen germination tables^27^. The second one, called Germinator, also proved efficient for automatic germination scoring from seeds sown in individual transparent trays^26^. SeedGerm^31^ uses Raspberry Pi controllers. Yet, these systems suffer from limiting factors to achieve the goal of high-throughput screening. Notably, manual seed sowing is time consuming and limits the throughput capacities of these systems. Likewise the relatively high volume of the germination medium is limiting for screening chemical library of natural extracts for most of them available in small quantities.

**Figure 1:**
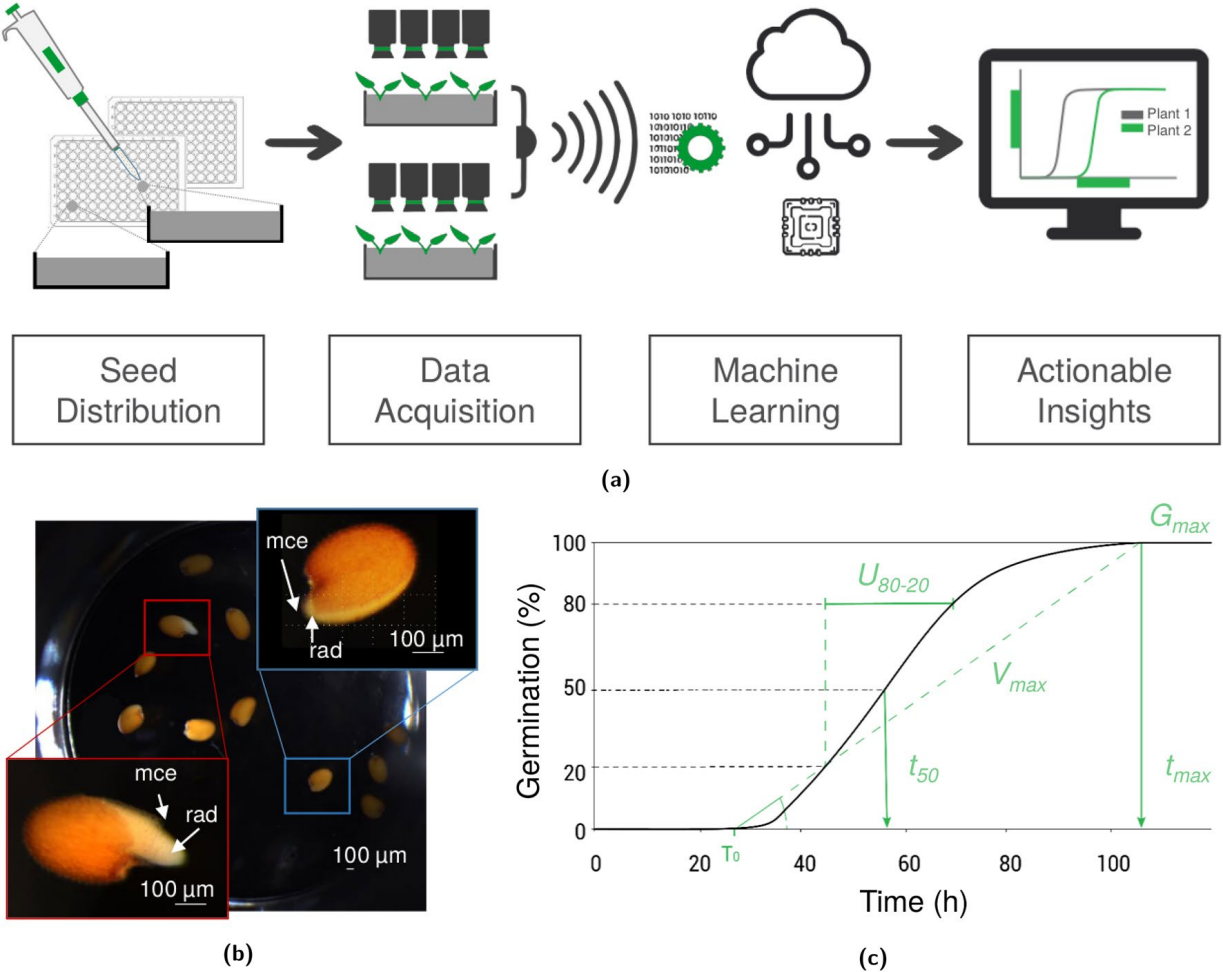
The ScreenSeed procedure workflow. (**a**) Seeds are sown by a pipetting method in microplates that are deposited in an automate (see Supplementary Fig. S1 online) taking hourly pictures of each well (see Supplementary Data S2 online). The images are transferred by Internet connection to a database for computational analysis and seed germination scoring. The software processing provides germination kinetics and extracts metrics with representation systems and statistical analyses that help to compare seed quality in a dashboard. (**b**) Imaging detection of germinated seeds. The imaging software was trained to score germinated seeds in microplate wells. In the microplate wells, the software identified germinated seeds (red square) among the detected seeds. A germinated seed illustrated by radicle protrusion through micropylar endosperm (see red square magnification details) is compared to non-germinated seed that only broke the testa but not the micropylar endosperm cap surrounding the radicle tip (see blue square magnification details). mce: micropylar endosperm; rad: radicle. The scale bar indicates $100\,\upmu \hbox {m}$. (**c**) Metrics of germination kinetics provided by the ScreenSeed dashboard. The kinetic curve is represented by cumulative germination percentage scored every hour during seed hydration. The metrics $G_{max}$ (maximal percentage of germination), $U_{80-20}$ (80/20 time spread it the time interval between 20% and 80% germination), $t_{50}$ (time required to reach 50% of the $G_{max}$) are automatically extracted from the database and can be compared between samples (Table 3).

In this paper, we present a novel seed germination tool named ScreenSeed. The procedure (Fig. 1a) uses 96-well microplates to sow seed samples in 96 splitted blocks and compact robots (described in Supplementary Fig. S1 online) providing automated image acquisition every hour and exporting the data to a dedicated database. A time series of images of a well can be seen in Supplementary data S2 online. These data are then used to score individual germination events, thereby delivering germination kinetic parameters of the analyzed seed samples. The ScreenSeed procedure benefits from a fast sowing method delivering droplets of Arabidopsis seed suspensions. Moreover, the use of 96-well microplates enables to separate blocks of samples thereby providing the independence between the wells and thus to use very small amounts of germination medium and chemicals potentially affecting seed germination. These characteristics make this procedure suitable for high-throughput screening of numerous Arabidopsis seed samples, genotypes, as well as chemical products or seed treatments to improve seed vigor. In this work, we present a critical analysis of the reliability of this automated screening tool in comparison to other described germination tests based on visual inspection of germinating seeds.

**Table 1:** Results of nine different tests to evaluate the edge effect risk with the Kruskal–Wallis test.

Measure	Group tested	p-value	Conclusion
$t_{50}$	Ring	0.5238	Not Significant
	Row	0.3381	Not Significant
	Column	0.8547	Not Significant
$U_{80-20}$	Ring	0.7795	Not Significant
	Row	0.3797	Not Significant
	Column	0.9724	Not Significant
$G_{max}$	Ring	0.9615	Not Significant
	Row	0.99998	Not Significant
	Column	0.9999992	Not Significant

The Measure column indicates the physiological index that is tested while the Group Tested column indicates which group factor (Column, Row, Ring) is assessed. The p-values are computed from 374 observations that accounts for 4804 seeds. The column Conclusion depicts that no significant edge effect has been identified.

**Figure 2:**
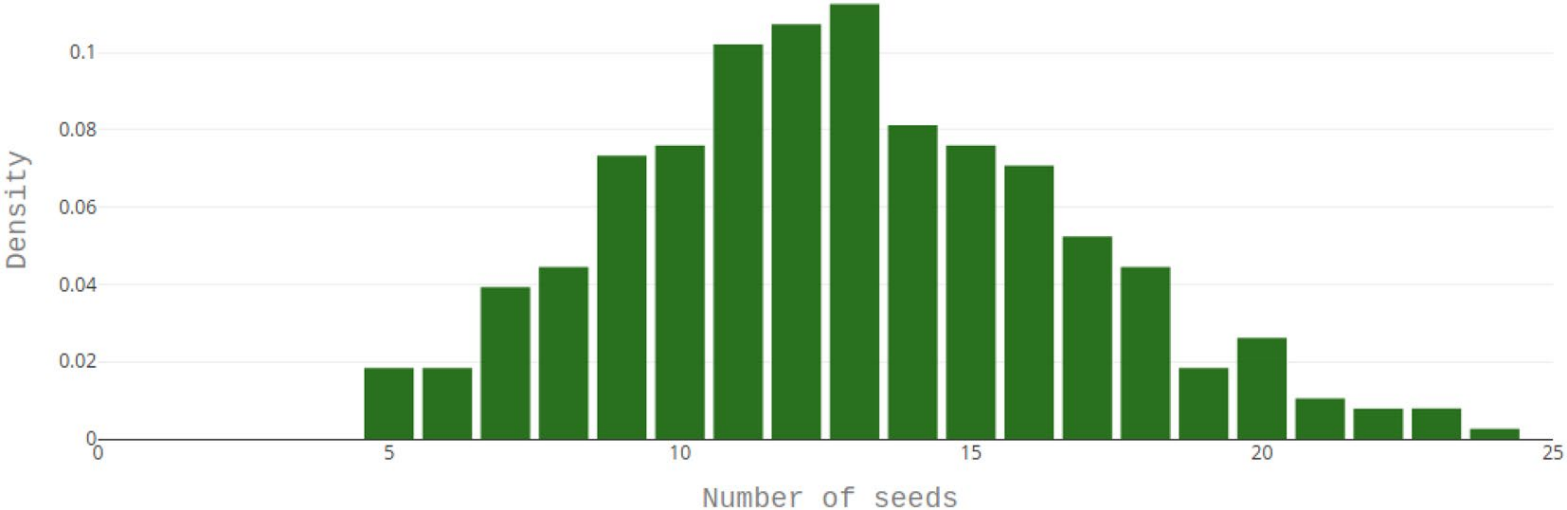
Observed distribution of the number of seeds deposited per well of microplates. In the replicates used in this study, the number of seeds observed in each well varied between 5 and 24 seeds with an average number of 13 seeds and a standard deviation of 4.

**Table 2:** Evaluation of the the impact of the number of seeds (*s*) on four physiological indices ($G_{max}$, $t_{20}$, $t_{50}$ and $t_{80}$) was achieved by linear regression estimations by ordinary least square analysis.

Model	${\hat{\alpha }}$ (std)	${\hat{\alpha }}$ p-value	C.I. ${\hat{\alpha }}$ ($90\%$)	${\hat{\beta }}$ (std)	${\hat{\beta }}$ p-value	C.I. ${\hat{\beta }}$ ($90\%$)	$R^{2}$
$G_{max} = \alpha + \beta s + \xi$	0.977 (0.01)	$1.7 \times 10^{-278}$	[0.962, 0.993]	0 (0.001)	0.58	[− 0.002, 0.001]	0.001
$t_{20} = \alpha + \beta s + \xi$	37.29 (1.1)	$4.70 \times 10^{-118}$	[35.5, 39.1]	0.44 (0.080)	$8.88 \times 10^{-7}$	[0.30, 0.57]	0.074
$t_{50} = \alpha + \beta s + \xi$	39.98 (1.2)	$3.48\times 10^{-109}$	[37.9, 42.0]	0.68 (0.092)	$9.42 \times 10^{-12}$	[0.53, 0.83]	0.128
$t_{80} = \alpha + \beta s + \xi$	44.24 (1.6)	$3.65\times 10^{-91}$	[41.6, 46.9]	0.95 (0.119)	$2.15\times 10^{-13}$	[0.75, 1.15]	0.145

The column Model defines the estimated linear regression where $\xi$ is a Gaussian noise, $\alpha$ is the intercept and $\beta$ the slope of the model. Estimated value of the intercept (${\hat{\alpha }}$) and the slope (${\hat{\beta }}$) are shown with their standard error values in bracket. Two-sided p-values against the null hypotheses that the coefficients $\alpha$ and $\beta$ are null are shown. The 90% symmetrical confidence intervals (C.I.) of each coefficient are displayed. The $R^2$ values of the linear models are listed.

**Figure 3:**
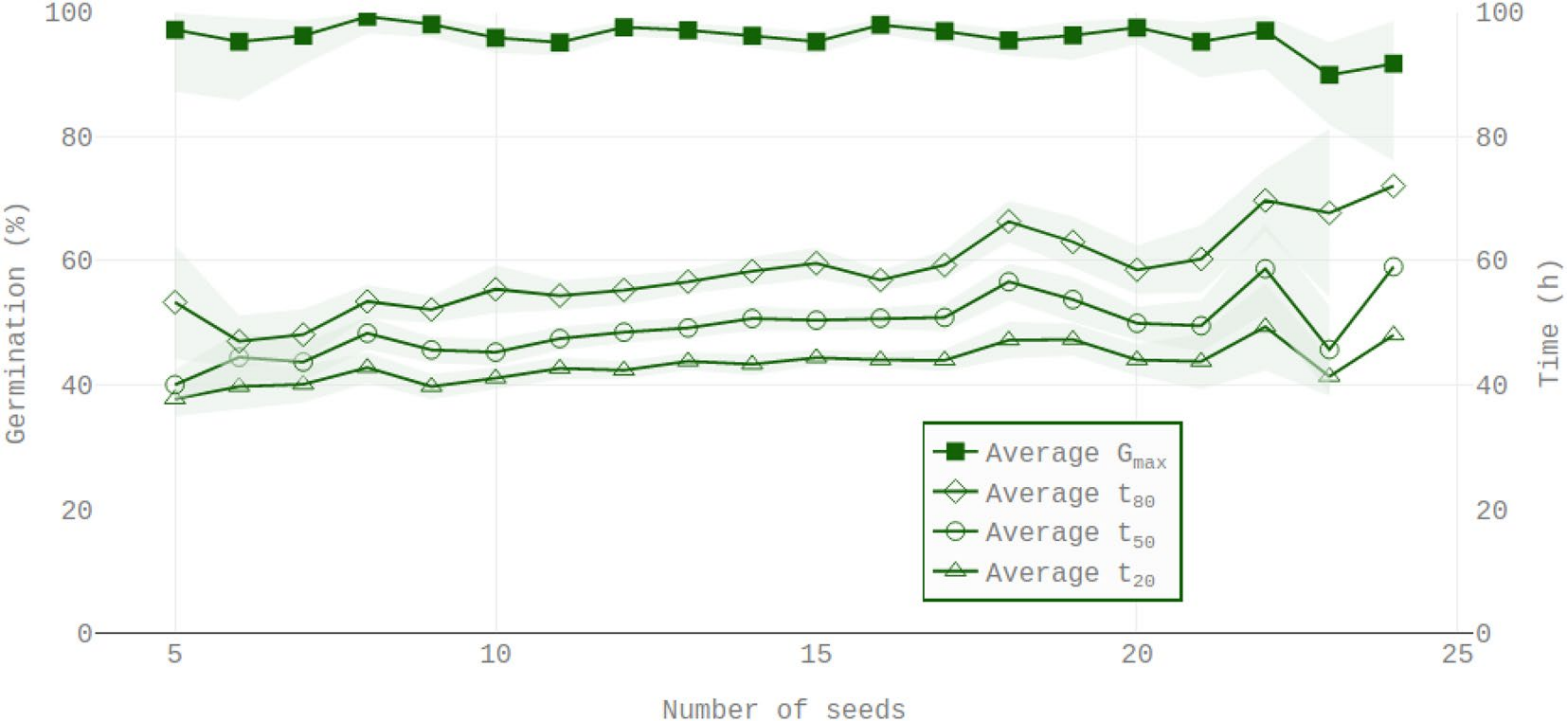
Average observed values of $G_{max}$, $t_{20}$, $t_{50}$ and $t_{80}$ conditionally to the number of seeds in each well. This figure is drawn over 374 observations and 4804 seeds. Left scale is germination percentage and right scale is related to time quantity ($t_{20}$, $t_{50}$ and $t_{80}$). Green transparent areas are 90% confidence intervals. Regarding time quantities ($t_{20}$, $t_{50}$ and $t_{80}$) the confidence intervals are based on Gaussian distributions. For proportion quantities ($G_{max}$), Fisher’s exact test is used to compute asymmetric confidence intervals.

**Figure 4:**
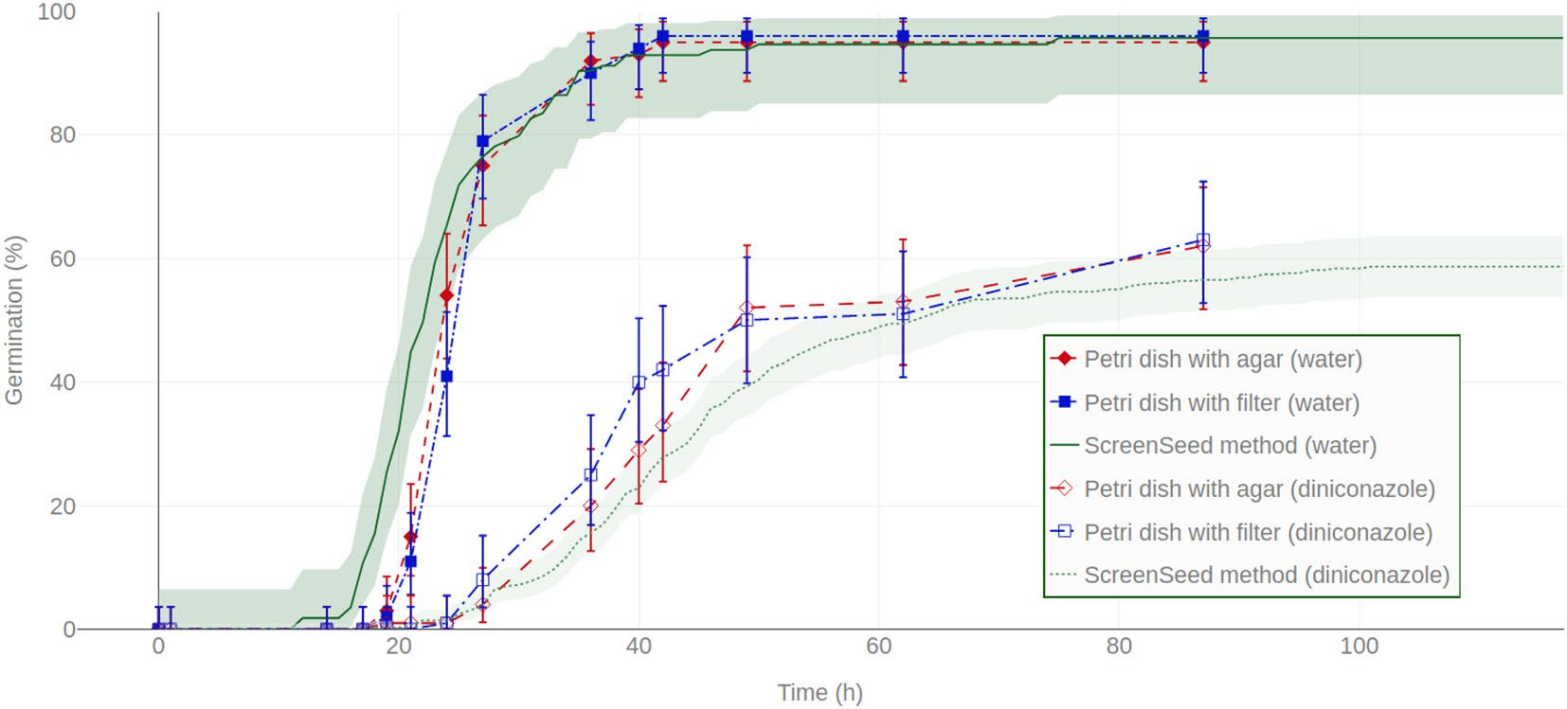
Comparison of seed germination kinetics obtained with the ScreenSeed robot and standard visual-based methods. Germination kinetic curves obtained with ScreenSeed phenotyping tools (line and dots for water and $10\,\upmu \hbox {M}$ diniconazole conditions respectively) are compared to standard methods (visual monitoring of seed germination) in Petri dishes using filter paper (square) or agar medium (diamonds), in water (filled symbols) or in $10\,\upmu \hbox {M}$ diniconazole (empty symbols), respectively. Shaded areas around the curves obtained using ScreenSeed technology represent the 90% confidence intervals matching Fisher Exact test as described in^50^. For standard methods by visual scoring in Petri dishes, the average of 4 blocks of replicates is shown with error bars representing the 90% Fisher Exact test confidence interval. As described in Supplementary Data S4 online, 100 seeds have been used for Petri dishes conditions. Regarding observation from the ScreenSeed robot, 55 seeds for the non treated (water) condition and 404 seeds for the $10\,\upmu \hbox {M}$ diniconazole conditions were observed respectively.

**Figure 5:**
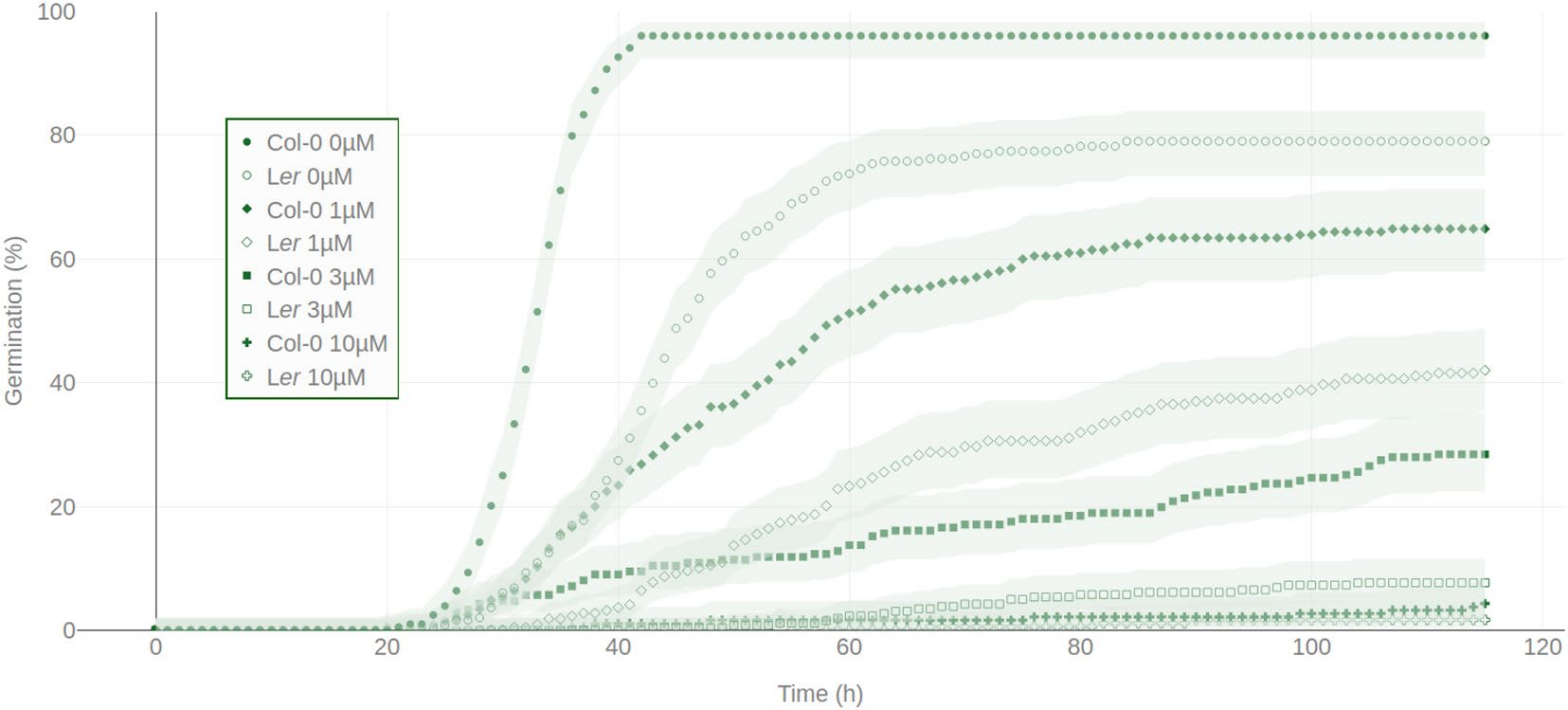
Comparison of ABA dose responses for germination inhibition of L*er* and Col-0 accessions. Germination kinetics of L*er* (diamonds and crosses) and Col-0 (dots and squares) were monitored in water (filled dots and diamonds) condition and in $1\,\upmu \hbox {M}$ (empty dots and diamonds), $3\,\upmu \hbox {M}$ (filled squares and crosses) or $10\,\upmu \hbox {M}$ (empty square and crosses) ABA. Each germination curve scored by a symbol in the graph is an average ± standard error of a representative experiment (12 blocks of replicates). Shaded area around each curve represents the 90% confidence intervals matching Fisher Exact test as described in^50^. Except for ABA at $10\,\upmu \hbox {M}$ that completely inhibited seed germination (c.f. Table 3), L*er* germination kinetics exhibited a significant higher ABA sensitivity compared to Col-0.

## Materials and methods

### Plant material

The seeds used in this study originated from the Arabidopsis accessions Columbia (Col-0) and Landsberg *erecta* (L*er*). They were obtained from the ABRC (Arabidopsis Biological Resource Center) and were produced in controlled conditions using a culture chamber (Memmert ICP 750) at 20 °C with a 16-h photoperiod of light ($120\,\upmu \hbox {mol}$ photons $\hbox {m}^{-2}\,\hbox {s}^{-1}$) at a relative humidity of 70% (RH). After seed maturation on the mother plants, Arabidopsis plants were submitted to water deprivation for three weeks and mature seeds were harvested by shaking stems with mature, dry siliques into large paper bags. Then seeds were stored at 7 °C, 40% RH in airtight tubes (Eppendorf 2 mL, Sigma).

### Germination assay

Prior to sowing, seeds were suspended in agarose 0.05% (w/v) at a density of 3 g $\hbox {L}^{-1}$ for 1 to 2 min, which is the time needed for stable seed homogenization. Agarose is polymerized in water by warming in a microwave and will be used at room temperature. Agarose 0.05% (w/v) remains in a liquid and somewhat viscous state at room temperature (i.e. 20 °C) which allows homogenous seed distribution by pipetting. Seeds were then distributed manually in 96 microplate wells (Greiner Bio-One, ref 655101 Germany) by sequential deposit of $8\,\upmu \hbox {L}$ (corresponding 5 to 24 seeds) of seed suspensions per well using a $200\,\upmu \hbox {L}$ pipette (Gilson) fitted with a cut-off tip. The final volume was adjusted to $200\,\upmu \hbox {L}$ per well with the appropriate liquid germination medium. Plates were then covered with lids treated by Tween 20 (Sigma-Aldrich)) in order to avoid evaporation while curbing condensation on the lids. Lids were sealed with a plastic film and incubated directly in the ScreenSeed automate inside a thermo-regulated incubator (Memmert ICP 750) regulated at 20 °C ($\pm \,1\,^{\circ }\hbox {C}$). It can be noticed that the used plastic film is a low-density polyetylene clingfilm characterized for its high oxygen permeability coefficient and is not limiting for oxygen supply^36^. To limit temperature variation in the plate between different experiments, the automate was always positioned exactly at the same place in the middle of the incubator. Abscisic acid (ABA; Sigma-Aldrich) and diniconazole (Sigma-Aldrich) solutions were prepared in purified water using a Millipore Milli-Q gradient water purification system. The ScreenSeed robot placed inside the incubator allowed monitoring seed germination with a given frequency (every hour in the context of this study) through an automated image acquisition system (Raspberry Pi Camera Module V2.1) that was externally controlled through an Internet connection using in-house software and hardware technology (Fig. 1a). This enabled the germination behavior of seed samples to be monitored every hour. For comparative purposes, germination tests in Petri dishes (Greiner Bio-one, diameter 9 cm) were carried out under the same incubation conditions (temperature, light and hygrometry) as for the analyses carried out in microplates (see Supplementary Data S3 online). The seeds (100 seeds per condition) were sown either on a water-soaked Whatman Grade filter paper ($80\,\upmu \hbox {L\,cm}^{-2}$) or on 0.8% (w/v) agarose (Dutscher). Four replicates were run in each condition analyzed (Supplementary Data S4 online). Seed germination was scored using a binocular magnifier (Olympus SZX10).

### Computational data analyses

Images of seeds were acquired every hour and were saved in an in-house database for computational analyses. To score that a seed has completed its germination, the software had been trained to consider the event of radicle protrusion through the endosperm layer of the seed (see Fig. 1b). The reliability of the annotation by this algorithm was estimated to be 99% for the determination of the number of seeds per well and 98% for the characterization of the germination event with time resolution of 1 h. All samples were also evaluated by visual inspection, which guaranteed a standardized annotation methodology uncorrelated to image recognition algorithms performance. Data visualization was provided by an in-house developed dashboard. This dashboard is designed for easy visual comparisons of metrics related to seed quality (ScreenSeed Lab.) and enables the download of tabular files for further analyses from the raw data. The software automatically determines the main metrics of seed germination kinetics of a seed sample (Fig. 1c), namely the maximal percentage of germination among total seeds ($G_{max}$), the time to reach 50% of germination ($t_{50}$), and the 80/20 time spread of germination ($U_{80-20}$, often called uniformity of germination), which is the time interval between the seeds reaching 20% to 80% of germination. All figures and tables reporting statistical results from the present study were acquired from this dashboard. Similar metrics can be computed from the R package *germinationmetrics*^37^. In this paper, the metrics relative to the time of germination are relative, in the sense that they measure the time to reach a given percentage of germination among the seeds that have germinated. Alternative metrics that are interesting as well are absolute time of germination, which measures the time needed to reach a given percentage of germination regardless of the final germination percentage. Relative and absolute measures are both accessible, however, this paper will rely on relative measure for the sake of the analyses. All the germination time seed by seed in the analyses are provided in the Supplementary Data S3 online for Col-0 accession in water condition, in Supplementary Data S4 online for comparison with standard assays and in Supplementary Data S5 online for the analyse of Col-0 and L*er* ABA dose response. From these data, we show that the time of germination would enable quantification of ABA sensitivity.

### Statistical analyses

Statistical tests were used to evaluate potential undesired biases or random effects associated with the ScreenSeed procedure. To this end, different methods were applied. When quantities corresponded to a time interval such as $t_{50}$ or $U_{80-20}$, the Kruskal-Wallis test was used^38^. This test is a non-parametric method based on ranks of the sample observations. It is considered as the non-parametric equivalent of the well-known one-way ANOVA test and is more robust when non-Gaussian data are observed. For estimating proportion descriptors of seed germination (e.g. germination percentage), binomial tests were used. One should emphasize that special care should be taken when proportions are close to lower (0%) and upper (100%) limits. It is common to see in the literature that the z-test (based on central limit theorem) is used for the comparison of proportions. However, this test assumes that proportions are normally distributed, which is not a realistic assumption when a proportion is close to the lower or the upper bounds of the interval considered. In such situation, the z-test suffers from underestimating the variance and therefore to overestimate the p-value and to wrongly narrow the confidence interval. An extreme example of such a situation is when the proportion of a sample reaches $100\%$ of the sampled population. Then, using a z-test would imply a null confidence interval of size, which would not be realistic. For these reasons, in this article, Fisher’s exact tests were applied to statistically analyze observations of proportions. Proportions (e.g. germination percentage) were estimated by weight-averaging proportionally to the number of seeds in each well^39^. By considering that the number of seeds in each well is an independent random variable, such a method is more appropriate than the arithmetic average for binomial distributions comparisons. Since the number of seeds per well is a stochastic quantity, uniform averaging methods would overestimate the variance of the observations. This could hinder some effects that would be wrongly considered as insignificant.

## Results

### The ScreenSeed tool designed to automate Arabidopsis seed germination analyses

In this work, the criterion used to score that a seed has accomplished its germination is the developmental stage when elongating radicle is protruding from micropilar endosperm tissue (c.f. red rectangle in Fig. 1b). As illustrated in Fig. 1b, computational imaging analysis efficiently allowed distinguishing germinated seeds from non-germinated seeds (Fig. 1b). Seeds were considered as non-germinated when the testa remained intact or corresponded to the only testa rupture as illustrated in the blue rectangle of Fig. 1b.

### Assessing random variation effects

The use of random but fast distribution of seeds in microplates raised the question of uncontrolled effects on seed germination such as biases or increasing noise-to-signal ratio in the observed data. For this reason, we assessed the risk of edge effects with respect to well positions, and we also quantified the impact of the number of seeds per well on key indicators of seed germination. To this end, we considered the metrics $G_{max}$ (as illustrated in Fig. 1c), $t_{50}$ and $U_{80-20}$ in four microplate analyses (5 to 24 seeds per well). Three spatial effects have been studied, namely the column, row and ring positioning within the microplates (see Supplementary Fig. S6 online). A ring is defined by the distance of a well to the closest border of the microplate. Kruskal-Wallis tests applied to the metrics $t_{50}$ and $U_{80-20}$ provided p values higher than 1/3 for each type of group while the $\chi ^2$ test applied to $G_{max}$ was higher than 0.95 for every positional factor (Table 1). We conclude that no significant group effects can be evidenced.

**Table 3:** Comparison of average physiological indices regarding ABA dose response for germination inhibition of L*er* and Col-0 accessions.

Ecotype	ABA conc.	$G_{max}$ (std)	$t_{20}$ (std)	$t_{50}$ (std)	$t_{80}$ (std)	$U_{80-20}$ (std)	Nb. of wells
Col-0	0 μm	96.56 (8.3)	29.58 (2.15)	33.42 (1.62)	36.25 (1.71)	6.67 (2.35)	12
	1 μm	64.19 (30.4)	39.25 (7.70)	52.33 (14.58)	65.17 (16.83)	25.92 (15.01)	12
	3 μm	31 (18.53)	45.91 (23.89)	67 (26.74)	88.91 (18.91)	43 (23.96)	12
	10 μm	3.88 (6.53)	79.25 (32.39)	81.75 (33.09)	84.5 (6.5)	5.25 (3.54)	12
L*er*	0 μm	80.91 (16.06)	36.75 (5.31)	43.92 (3.58)	53.50 (6.5)	18.75 (6.05)	12
	1 μm	46.02 (24.26)	50.17 (13.22)	61.08 (12.31)	78.42 (17.24)	28.25 (22.82)	12
	3 μm	8.60 (8.84)	61 (12.84)	68.33 (16.81)	72.78 (20.80)	11.78 (17.58)	12
	10 μm	3.02 (4.16)	73.25 (15.99)	73.25 (15.99)	73.25 (15.99)	NA	12

In brackets are the standard deviations of the means. In stressed condition, only wells with more than 2 germinated seeds were used to compute the averages of $t_{20}$, $t_{50}$, $t_{80}$ and $U_{80-20}$ respectively. Treatments have been randomized (c.f. Supplementary Data S5 online). The experiment has been done from a single microplate in order to illustrate that expected effect of ABA can be detected from a single microplate. If one would like to draw conclusions regarding the impact of ABA on germination, multiple independent analyses would be required.

The present proposed protocol is based on pipetting Arabidopsis seed suspensions. However, this protocol entailed some variability in the number of seeds deposited in each well. We found that this number can vary from 5 to 24 seeds (Fig. 2). Despite such variation, an examination of the results in Table 2 and in Fig. 3 disclosed that there was no significant impact of the number of seeds on the maximal percentage of germination. Ordinary least square regressions were also carried out for the metrics $t_{20}$, $t_{50}$ and $t_{80}$ respectively (times to reach respectively 20%, 50% and 80% of seed germination) as a function of number of seeds per well (Table 2; Fig. 3). In contrast to $G_{max}$ determinations, the data showed that in the observed range of 5 to 24 seeds the $t_{20}$, $t_{50}$ and $t_{80}$ values slightly increased in a linear fashion with the number of seeds per well. Furthermore, the increase in $t_{80}$ with respect to the number of seeds was higher than that of $t_{50}$, which was higher than that of $t_{20}$ (Table 2 and Fig. 3). This indicates that the 80/20 time spread described by $U_{80-20}$ increased with the number of seeds per well although such increase was very small.

### Comparison with standard germination tests

The ScreenSeed germination phenotyping method was compared to other methods commonly used in seed testing laboratories. In these protocols, Arabidopsis seeds are generally sown in Petri dishes either in agarose medium or on imbibed filter paper. Then, germination is visually scored using binocular lens. Presently, such comparisons were carried out in water and in diniconazole. In water, as in diniconazole, the germination kinetics provided by the ScreenSeed phenotyping methods proved very similar to these obtained by standardized assays in agar or on blotting paper (Fig. 4). Supplementary Data S4 online described the raw germination data of this analysis.

### Assessing responsiveness of Arabidopsis accessions to exogenous ABA application

ABA is well described as a potent germination inhibitor^40–42^. By using the ScreenSeed method, the sensitivity to ABA of the two natural accessions Col-0 and L*er* has been evaluated (Fig. 5, Table 3 and Supplementary Data S5 online). By applying exogenously ABA to the germination medium in the concentration range of 0-$10\,\upmu \hbox {M}$, average $G_{max}$ values were 96% ($0\,\upmu \hbox {M}$), 64% ($1\,\upmu \hbox {M}$), 31% ($3\,\upmu \hbox {M}$) and 4% ($10\,\upmu \hbox {M}$), respectively for Col-0, and 81% ($0\,\upmu \hbox {M}$), 46% ($1\,\upmu \hbox {M}$), 9% ($3\,\upmu \hbox {M}$) and 2% ($10\,\upmu \hbox {M}$), respectively for L*er*. Also, $U_{80-20}$ and $t_{50}$ values increased with increasing ABA concentrations. For each ABA concentration, these metrics (Table 3) allowed to categorize germination behavior of the two examined Arabidopsis genotypes and provided useful parameters to quantify their ABA sensitivity (Fig. 5). Further analysis to quantify the sensitivity of genotypes can be applied from the acquired data (see Supplementary Data S5 online) as described in^43^. For the sake of illustration, results of such method are described in Supplementary Table S7 online.

## Discussion

Automated monitoring of seed germination by imaging has been previously developed as described by^26^ for the Germinator technology^29^ and by^27,28^ for real-time monitoring of seed germination in Jacobsen germination tables. Regarding these technologies, the relatively large volume of the germination media used can be a limiting factor in order to run high-throughput screening of chemical libraries in which the tested compounds are usually available in only tiny amounts. To circumvent this difficulty, we have in the present work miniaturized the imaging technology to monitor Arabidopsis seed germination behaviors using 96-well microplates.

Thanks to the small size of the seeds, i.e. $0.4\,\upmu \hbox {m}$ length, imaging-based automated annotation for scoring seed germination remained reliable for up to 24 seeds per microplate well. This allows identifying significant differences in germination behavior for high throughput prescreening experiments in the case we are looking for marked differences. However, for more in-depth analysis of the germination quality according to the criteria of ISTA standards, it would be necessary to multiply the conditions tested on several wells in the same plate and to aggregate in the analysis the germination scores on the total number of seeds tested in the same condition. Moreover four independent experiments have been replicated. This type of analysis was used in the study presented in Fig. 5 and does not present any bias due to the fact that there is no identified effect of the position of the wells on the microplate as shown in Table 1. Practically, the monitoring of seed germination proved feasible for nearly 2000 seeds per plate in a controlled environment. Furthermore, the designed procedure enabled hourly observations in each of the 96 independent assays run in the microplate wells.

In this study, we have examined the possible biases of this method that could interfere with seed germination behaviors. In this aim, statistical analyses allowed considering the variability of the main germination parameters (see Fig. 1c), i.e. $G_{max}$, $t_{50}$ and $U_{80-20}$, from collected analyses carried out under the same experimental conditions and from the same seed sample.

Statistical analyses showed that seed germination behaviors did not suffer from potential edge effect (see Supplementary Fig. S6 online), which can be of interest to further simplify experimental protocols. Also, the ring, column and row position effects on $G_{max}$, $t_{50}$ and $U_{80-20}$ have been evaluated and no effect could be statistically observed. One should note that, until proven otherwise, replicability between plates would remain challenging. Further seed-handling automation and well controlled environment would curb the impact of external parameters. Thus, control replicates remain necessary.

Manual sowing of Arabidopsis seed requires intense laboratory work. To overcome this limitation and maintain the high-throughput character of the screening procedure, we have chosen a pipetting method for seed sowing. However, this method showed that a variable number of seeds is distributed in each well (mainly between 5 and 24 seeds per well, Fig. 2). The $G_{max}$ values were not significantly affected by a variation of number of seeds per well (Fig. 3 and Table 2). Nevertheless, it is noticeable that when considering the $t_{50}$ parameter, the random number of seeds in each well slightly impacted the germination behavior of the seed population (Fig. 3). In general, the times needed to reach 20%, 50% or 80% of germination ($t_{20}$ , $t_{50}$ and $t_{80}$) increased with the number of seeds in the wells (Fig. 3), and significant positive correlations could be evidenced (Table 2). The accuracy of the hour-by-hour analysis makes it possible to identify an effect on the $t_{50}$ parameter which is less than 20 h (Fig. 3) between the rare extremes of seed concentration in the $200\,\upmu \hbox {L}$ medium (Fig. 3). This effect that would not have been detected by a standard daily monitoring of germination score raises the hypothesis of a possible competition mechanism between seeds within the microplate wells. One explanation could be a reduced oxygen availability in the imbibed seeds^44^. Alternatively, the imbibed Arabidopsis seeds might have released compounds inhibiting their germination^45,46^. However, we observed that this feature was marginal with respect to the intrinsic biological variability within the experiments (c.f. $R^2$ values in Table 2). Indeed, the intrinsic variability conditionally to a given number of seeds in a well was greater than the average effect due to increased number of seeds per well (Fig. 3 and Table 2).

The germination kinetics obtained by automated imaging in the microplates were compared with classical analyses in Petri dishes as described in most scientific publications. As shown in Fig. 4, germination behavior in the microplate wells did not significantly differ from standard protocol observations where seeds are sown on water-imbibed filter paper or on agarose (Fig. 4). In particular, the use of seed germination inhibitors (i.e. the ABA catabolism-inhibitor diniconazole) showed that such molecules affected the germination kinetics very similarly in both automated and visual-based methods.

For a comprehensive large-scale phenotyping process to be actionable, the described technology should be combined to additional software modules including a fast (or automated) annotation of seed germination (Fig. 1a) and a web application to analyze and share the observation and statistical results. The statistical analyses and figures of this paper have been drawn from such a ScreenSeed web application.

Comparison of the germination behavior of the seeds of the L*er* and Col-0 accessions incubated in a range of ABA concentrations (Fig. 5) illustrated the value of this tool to characterize with high resolution genotype sensitivity to this inhibitor. In all tested ABA concentrations, L*er* exhibited a lower $G_{max}$ than Col-0 (Table 3), which is in excellent agreement with the previously identified phenotypes of more pronounced dormancy and of higher ABA sensitivity in the L*er* accession compared to Col-0^21,47,48^. We conclude that the ScreenSeed technology is operational to screen products (molecules or treatments) for their positive or negative effects on seed vigor, as well as to explore responsiveness to these products among the well-characterized diversity of genetic resources in Arabidopsis, as well as in other plant species. The application of such phenotyping platform would extend capacities of research in seed biology. Moreover, it would provide valuable tools to evaluate seed samples quality in Arabidopsis genotypes collections and manage the renewing of seed stocks.

In this study, we have used hand pipetting to deliver seed suspensions and various solutions (water, ABA, diniconazole) in the wells of microplates. However, several pipetting robots are presently available (e.g. the OT-2 Robot), and therefore there would not be any difficulty in equipping the present ScreenSeed automate with such a pipetting robot, notably for applications aiming at screening large chemical libraries to find novel molecules affecting the seed germination process (rate, uniformity, damping off....) that can be used for the development of new priming and/or treatment procedures^49^. We anticipate that the present technology can be used to investigate the germination behavior of a broad panel of seeds from crop species.

## Supplementary information

Supplementary Information 1.

Supplementary Information 2.

Supplementary Information 3.

Supplementary Information 4.

Supplementary Information 5.

## Data Availability

The data supporting the findings of the present study are available from EffiSciency and the authors upon reasonable request.
