# Supplementary material for: ScreenSeed as a novel high throughput seed germination phenotyping method
Source: Sci Rep. 2021 Jan 14;11:1404. doi: 10.1038/s41598-020-79115-2 (PMC7809209; doi:10.1038/s41598-020-79115-2)
Supplement: Supplementary file 1 — Supplementary Information 1. [file 41598_2020_79115_MOESM1_ESM.pdf]

Scientific Reports

Article title: ScreenSeed – A novel high throughput seed germination phenotyping method

Authors: Nicolas Merieux, Pierre Cordier Marie-Hélène Wagner, Sylvie Ducournau, Sophie Aligon, Dominique Job, Philippe Grappin, and Edwin Grappin (edwin.grappin@screenseed.com)

Supplementary notes S1 – ScreenSeed hardware design

Design specification of the ScreenSeed automate. Around 2000 Arabidopsis seeds (an average of 20 seeds by well, 96 wells per microplate) are analyzed in a single run.

For the analysis:
- The compact machine (200 mm X 400 mm X 255 mm) is installed in a thermo-regulated incubator (Memmert, ICP750). It is positioned halfway up and always in the same place to limit temperature variations.
- Pictures are acquired every hour for the duration necessary to obtain germination kinetics (4 to 7 days).
- At each run, the microplate moves sequentially under the camera system (Raspberry Pi Camera Module V2.1) to allow individual shootings of each well of the microplate (cf. Supplementary Data S2 online).
- A white light (polychromatic LEDVero 30x30, 10W, 800lm, 4500K) enlightens the samples for the duration of each run.
- The distance between the camera lens and the microplate is 10 mm.
- The movements of the microplate (NEMA 17 Stepper Motor, XSOURCE) and the shots (Raspberry Pi Camera Module V2.1) are controlled using a Raspberry computer (Raspberry Pi 3 Model B+)
- Images are imported to a database hosted on a server using an internet connection.

(a)

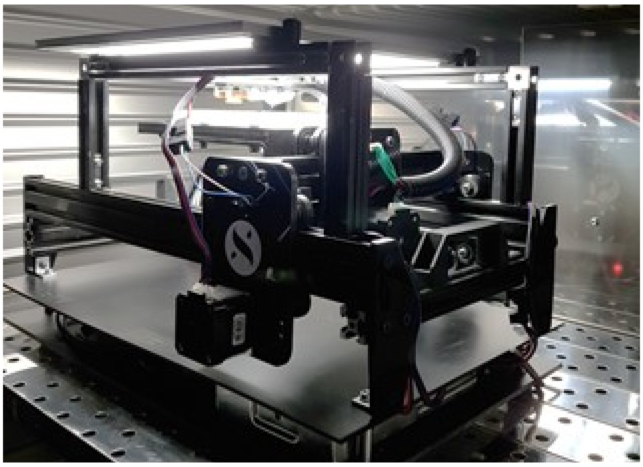

(b)

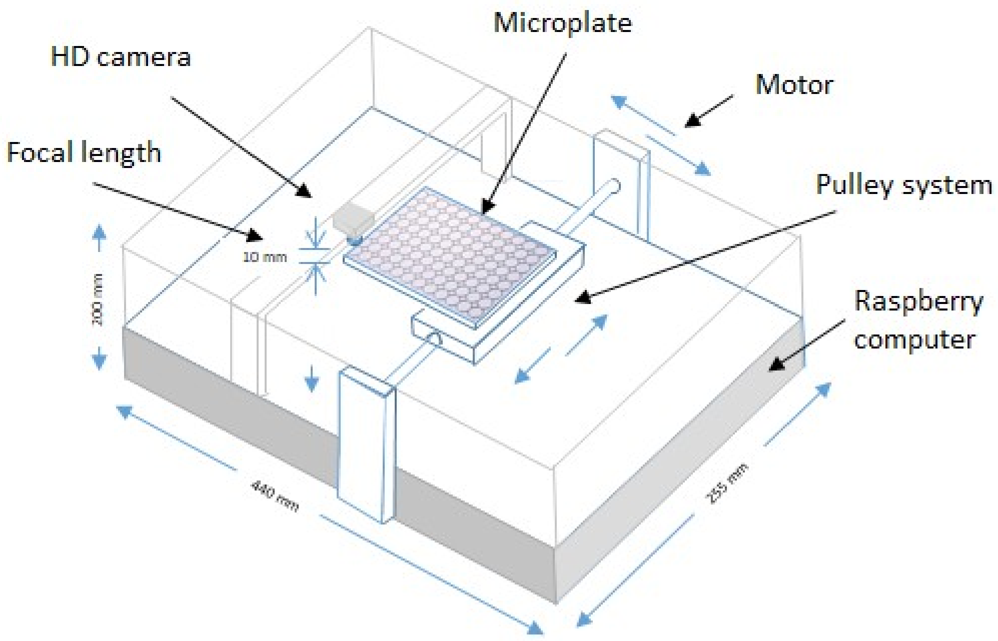

Supplementary Figure S1: ScreenSeed hardware design. (a) Shot of the robot in operation in its temperature-controlled chamber. (b) Schematic representation of its design.

Supplementary Dataset S2

Time series of shots on the well of coordinate D5 of a microplate. Each file results from a shot of the well hour by hour and is used to assess germination percentage at each hour of the analysis. Each of 96 wells of the microplate is sequentially shoot every hour.

C.f. Supplementary Dataset S2 (zip file)

Supplementary Dataset S3

Germination time seed by seed of Col-0 accession in water condition.

C.f. Supplementary Dataset S3 (xlsx file)

Supplementary Dataset S4

Comparison of germination assays of ScreenSeed tool analysis with standard conditions (i.e. petri dish blotting paper and petri dish agar conditions)

C.f. Supplementary Dataset S4 (xlsx file)

Supplementary Dataset S5

Responsiveness of Col-0 and L*er*Arabidopsis accessions to exogenous ABA application.

C.f. Supplementary Dataset S5 (xlsx file)

Supplementary Figure S6 – ScreenSeed hardware design

	Col. 1	Col. 2	Col. 3	Col. 4	Col. 5	Col. 6	Col. 7	Col. 8	Col. 9	Col. 10	Col. 11	Col. 12
Row A	Ring 1	Ring 1	Ring 1	Ring 1	Ring 1	Ring 1	Ring 1	Ring 1	Ring 1	Ring 1	Ring 1	Ring 1
Row B	Ring 1	Ring 2	Ring 2	Ring 2	Ring 2	Ring 2	Ring 2	Ring 2	Ring 2	Ring 2	Ring 2	Ring 1
Row C	Ring 1	Ring 2	Ring 3	Ring 3	Ring 3	Ring 3	Ring 3	Ring 3	Ring 3	Ring 3	Ring 2	Ring 1
Row D	Ring 1	Ring 2	Ring 3	Ring 4	Ring 4	Ring 4	Ring 4	Ring 4	Ring 4	Ring 3	Ring 2	Ring 1
Row E	Ring 1	Ring 2	Ring 3	Ring 4	Ring 4	Ring 4	Ring 4	Ring 4	Ring 4	Ring 3	Ring 2	Ring 1
Row F	Ring 1	Ring 2	Ring 3	Ring 3	Ring 3	Ring 3	Ring 3	Ring 3	Ring 3	Ring 3	Ring 2	Ring 1
Row G	Ring 1	Ring 2	Ring 2	Ring 2	Ring 2	Ring 2	Ring 2	Ring 2	Ring 2	Ring 2	Ring 2	Ring 1
Row H	Ring 1	Ring 1	Ring 1	Ring 1	Ring 1	Ring 1	Ring 1	Ring 1	Ring 1	Ring 1	Ring 1	Ring 1

Supplementary Figure S6: Representation of a microplate wells grouped in 12 different columns (from Col. 1 to Col. 12), in eight different rows (from Row A to Row H), and in four different rings (from Ring 1 to Ring 4).

Supplementary Table S7 – Sensitivity to ABA table

Genotype	Factor	ABA Time Constants	Mean Base Values	σ	r^2
Col-0	ABA	-54.98 log(M) h	1.72 µM	-0.68 log(M)	0.88
L*er*	ABA	-65.73 log(M) h	0.64 µM	-0.73 log(M)	0.85

Supplementary Table S7: Model of ABA sensitivity of L*er* and Col-0 genotypes from germination data of Supplementary Data S5. The notations follows[1], where Mean Base Values is the estimated ABA dose to inhibit the germination to 50%, and σ and r^2 are respectively the standard deviation of its estimation and the coefficient of determination the probit regression.

[1] Bradford, K. J. A water relations analysis of seed germination rates.Plant Physiol.94, 840–849 (1990).
