# Supplementary figures and images for: ScreenSeed as a novel high throughput seed germination phenotyping method (part 2 of 2)
Source: Sci Rep. 2021 Jan 14;11:1404. doi: 10.1038/s41598-020-79115-2 (PMC7809209; doi:10.1038/s41598-020-79115-2)

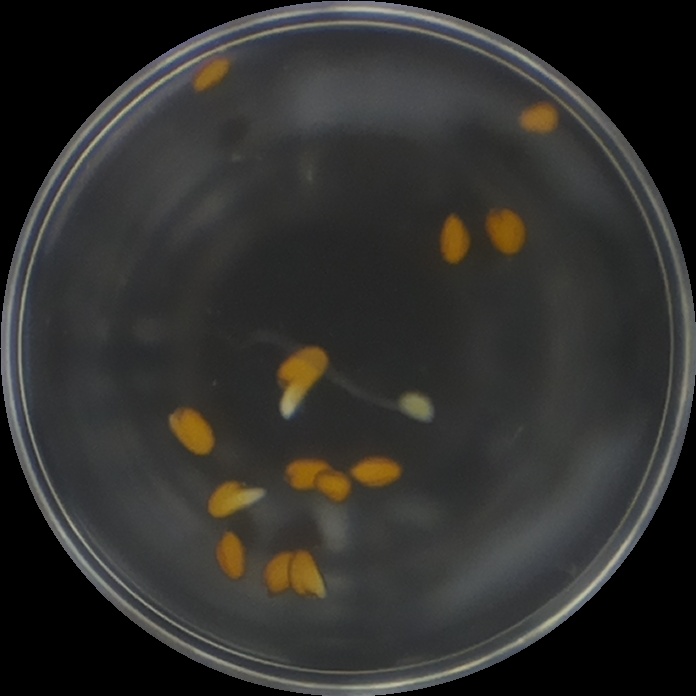

Supplement: Supplementary file 2 — Supplementary Information 2. [file 41598_2020_79115_MOESM2_ESM.zip › PictureOneWell/D5/94_593174]

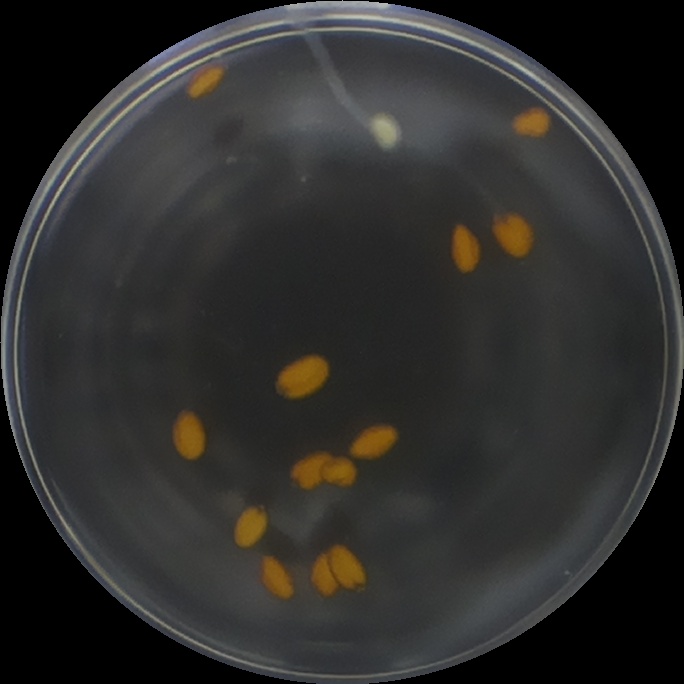

Supplement: Supplementary file 2 — Supplementary Information 2. [file 41598_2020_79115_MOESM2_ESM.zip › PictureOneWell/D5/30_580949]

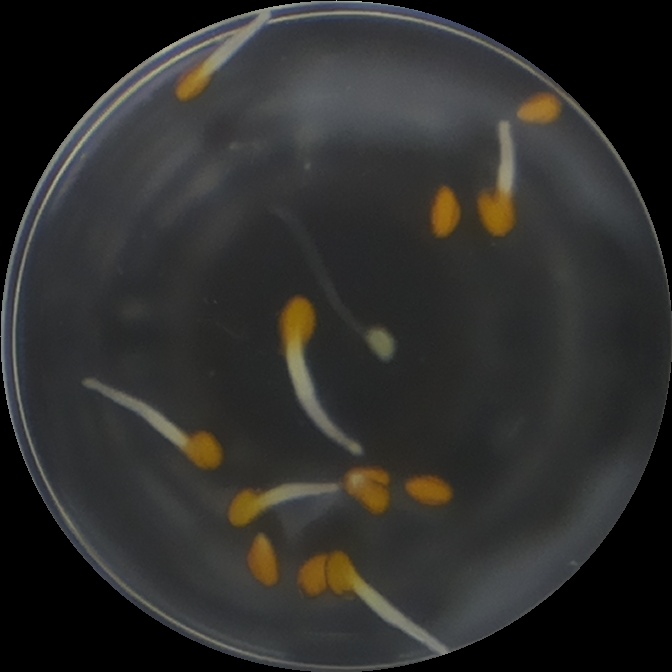

Supplement: Supplementary file 2 — Supplementary Information 2. [file 41598_2020_79115_MOESM2_ESM.zip › PictureOneWell/D5/150_604099]

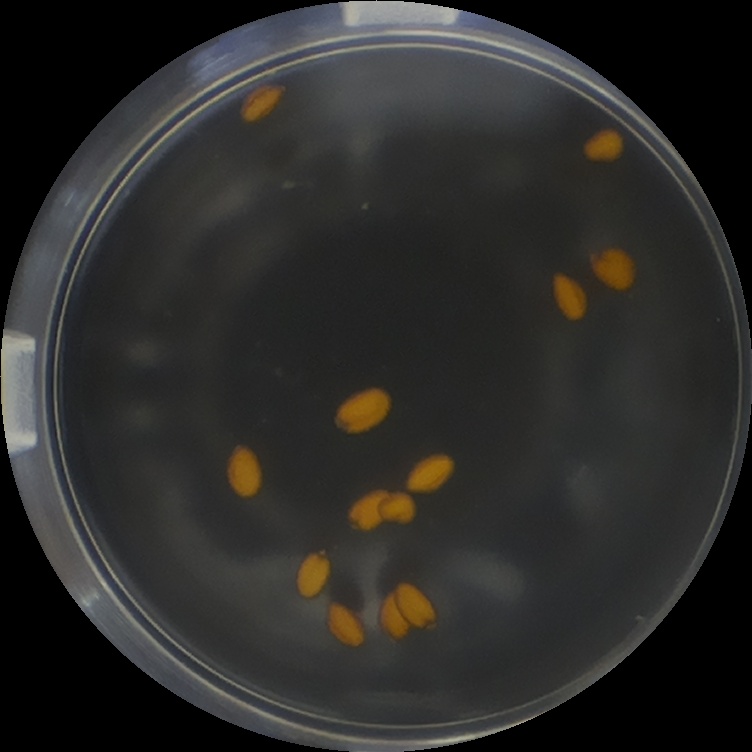

Supplement: Supplementary file 2 — Supplementary Information 2. [file 41598_2020_79115_MOESM2_ESM.zip › PictureOneWell/D5/9_577112]

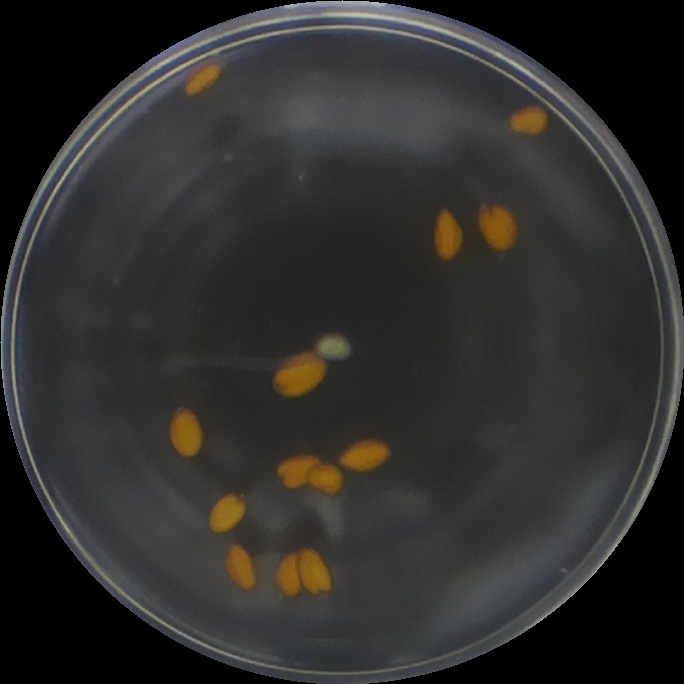

Supplement: Supplementary file 2 — Supplementary Information 2. [file 41598_2020_79115_MOESM2_ESM.zip › PictureOneWell/D5/49_584522]

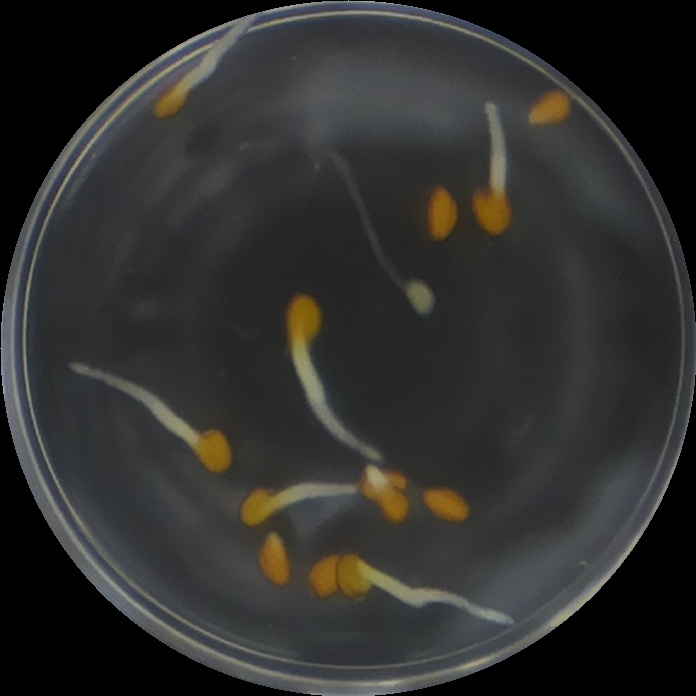

Supplement: Supplementary file 2 — Supplementary Information 2. [file 41598_2020_79115_MOESM2_ESM.zip › PictureOneWell/D5/158_605605]

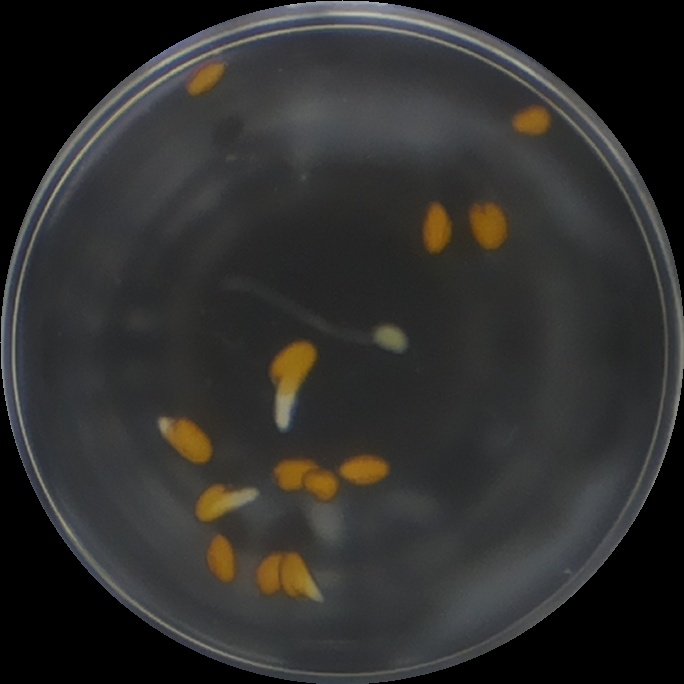

Supplement: Supplementary file 2 — Supplementary Information 2. [file 41598_2020_79115_MOESM2_ESM.zip › PictureOneWell/D5/104_595157]

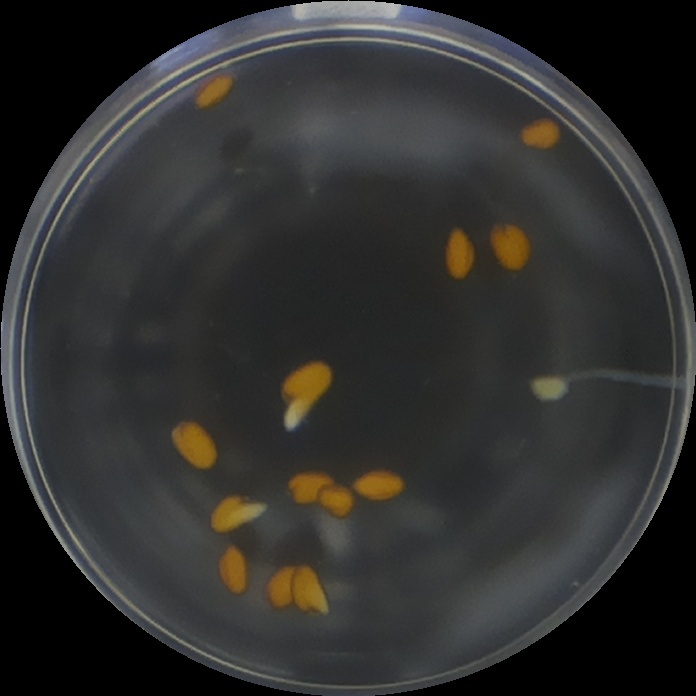

Supplement: Supplementary file 2 — Supplementary Information 2. [file 41598_2020_79115_MOESM2_ESM.zip › PictureOneWell/D5/93_593029]

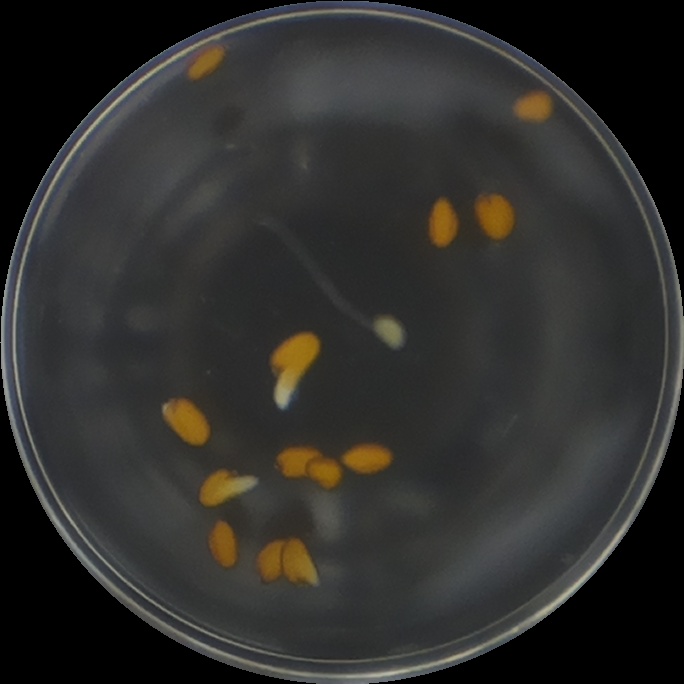

Supplement: Supplementary file 2 — Supplementary Information 2. [file 41598_2020_79115_MOESM2_ESM.zip › PictureOneWell/D5/97_593824]

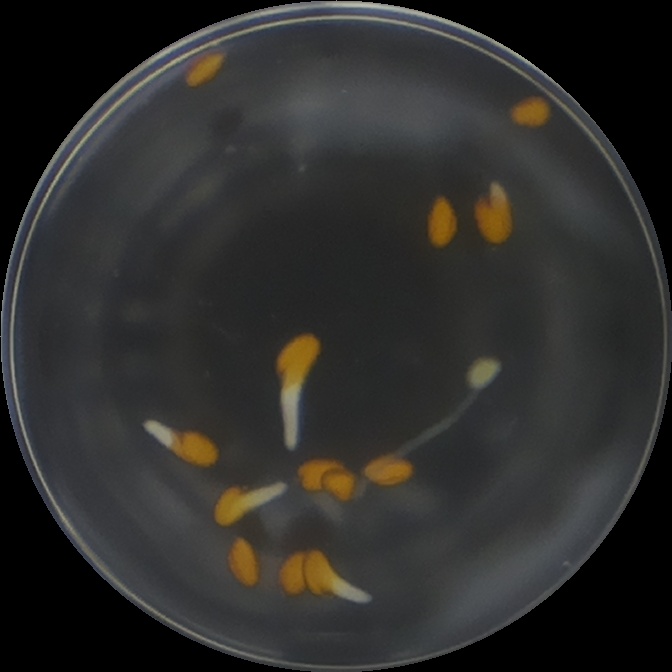

Supplement: Supplementary file 2 — Supplementary Information 2. [file 41598_2020_79115_MOESM2_ESM.zip › PictureOneWell/D5/116_597473]

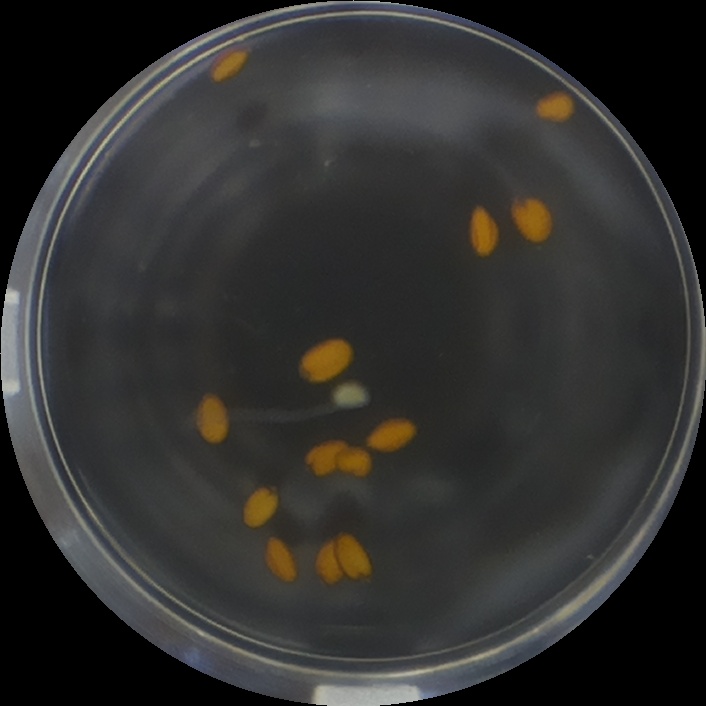

Supplement: Supplementary file 2 — Supplementary Information 2. [file 41598_2020_79115_MOESM2_ESM.zip › PictureOneWell/D5/44_583576]

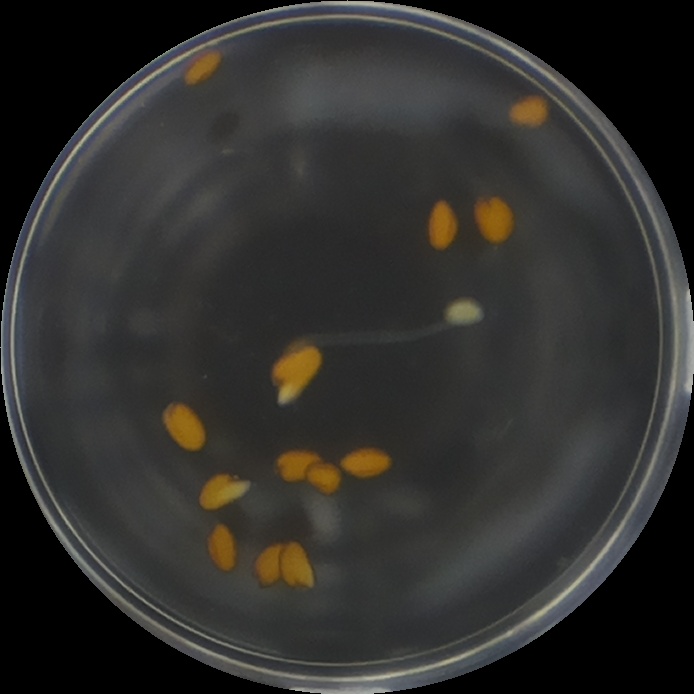

Supplement: Supplementary file 2 — Supplementary Information 2. [file 41598_2020_79115_MOESM2_ESM.zip › PictureOneWell/D5/84_591305]

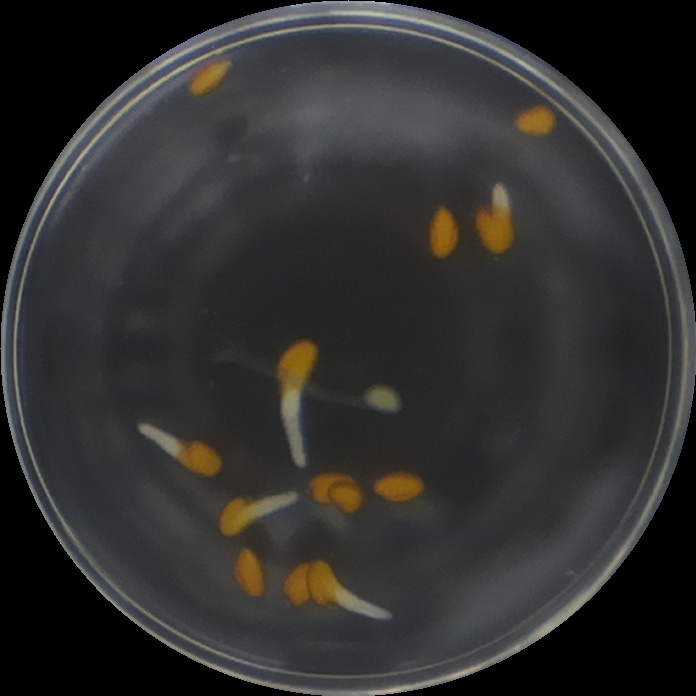

Supplement: Supplementary file 2 — Supplementary Information 2. [file 41598_2020_79115_MOESM2_ESM.zip › PictureOneWell/D5/122_598598]

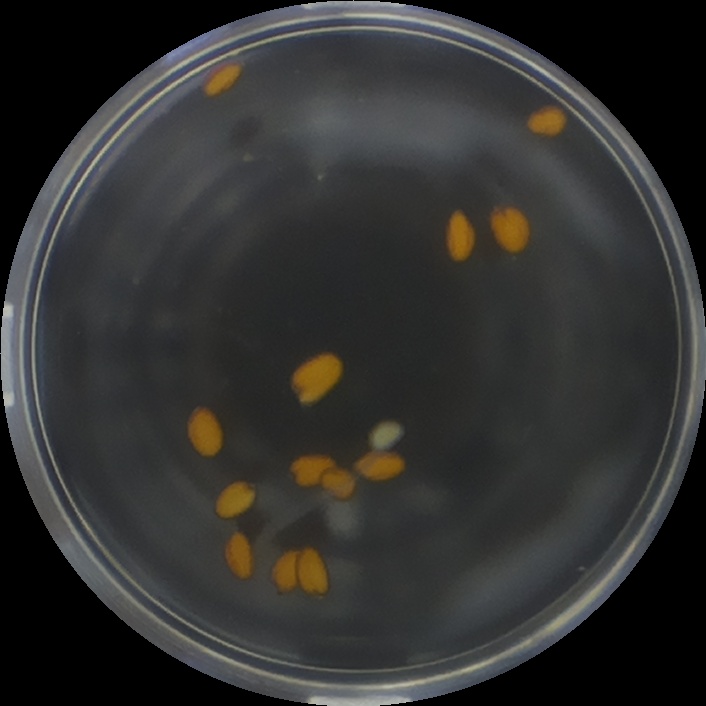

Supplement: Supplementary file 2 — Supplementary Information 2. [file 41598_2020_79115_MOESM2_ESM.zip › PictureOneWell/D5/71_588750]

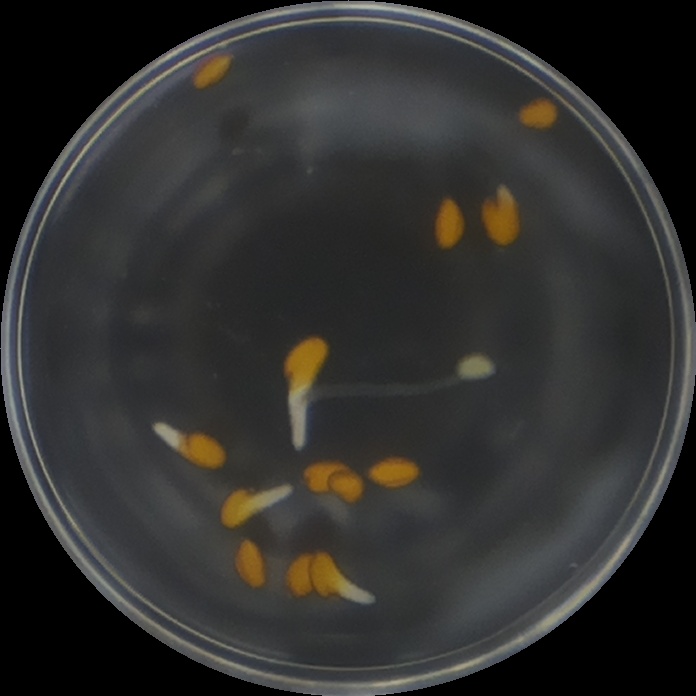

Supplement: Supplementary file 2 — Supplementary Information 2. [file 41598_2020_79115_MOESM2_ESM.zip › PictureOneWell/D5/115_597207]

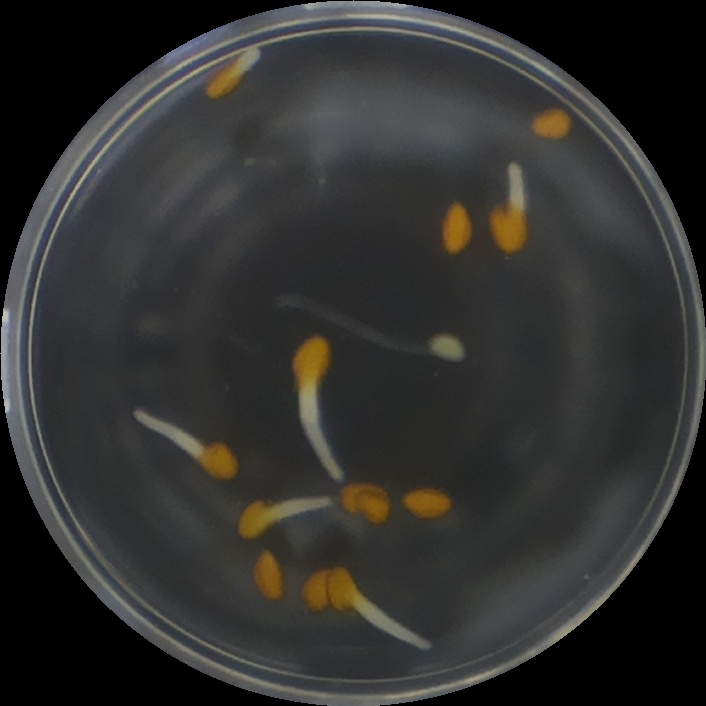

Supplement: Supplementary file 2 — Supplementary Information 2. [file 41598_2020_79115_MOESM2_ESM.zip › PictureOneWell/D5/135_601095]

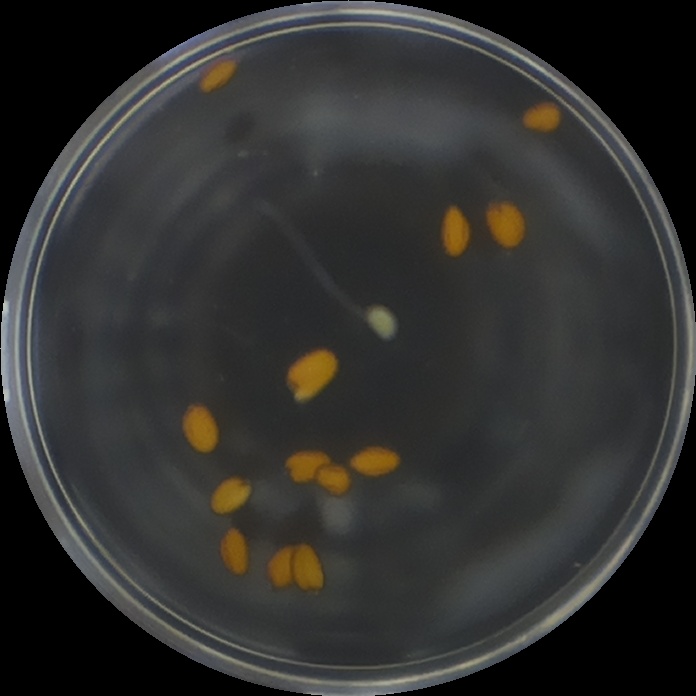

Supplement: Supplementary file 2 — Supplementary Information 2. [file 41598_2020_79115_MOESM2_ESM.zip › PictureOneWell/D5/73_589203]

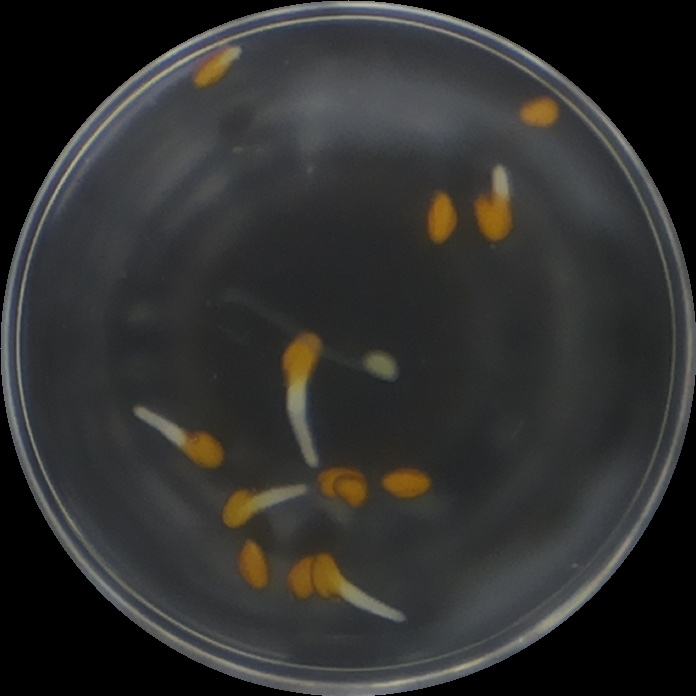

Supplement: Supplementary file 2 — Supplementary Information 2. [file 41598_2020_79115_MOESM2_ESM.zip › PictureOneWell/D5/127_599551]

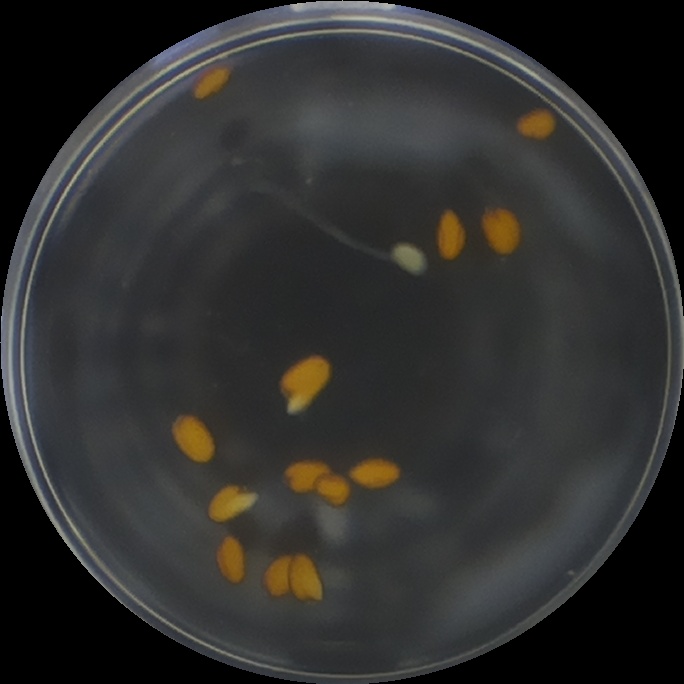

Supplement: Supplementary file 2 — Supplementary Information 2. [file 41598_2020_79115_MOESM2_ESM.zip › PictureOneWell/D5/82_590874]

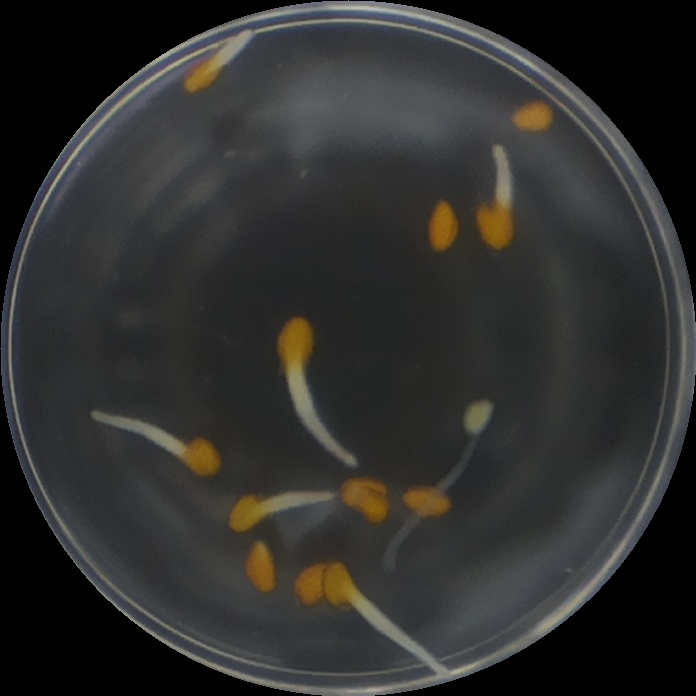

Supplement: Supplementary file 2 — Supplementary Information 2. [file 41598_2020_79115_MOESM2_ESM.zip › PictureOneWell/D5/144_602911]

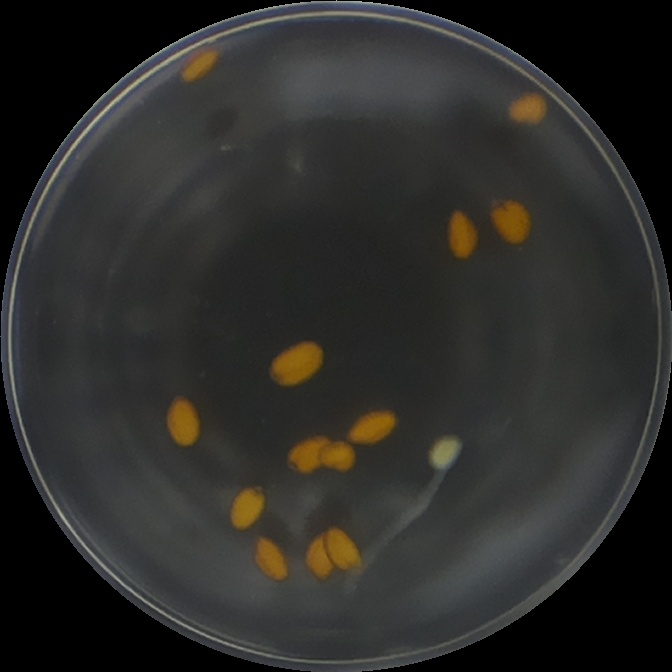

Supplement: Supplementary file 2 — Supplementary Information 2. [file 41598_2020_79115_MOESM2_ESM.zip › PictureOneWell/D5/35_581845]

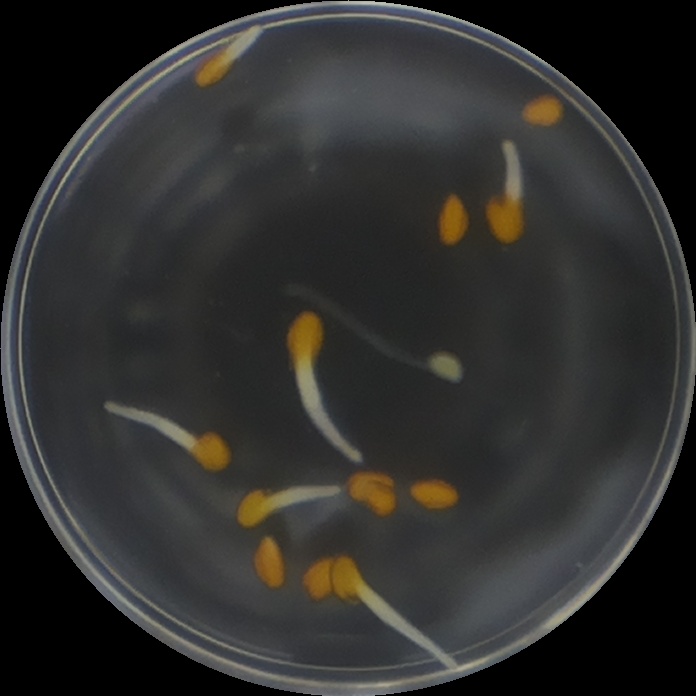

Supplement: Supplementary file 2 — Supplementary Information 2. [file 41598_2020_79115_MOESM2_ESM.zip › PictureOneWell/D5/143_602696]

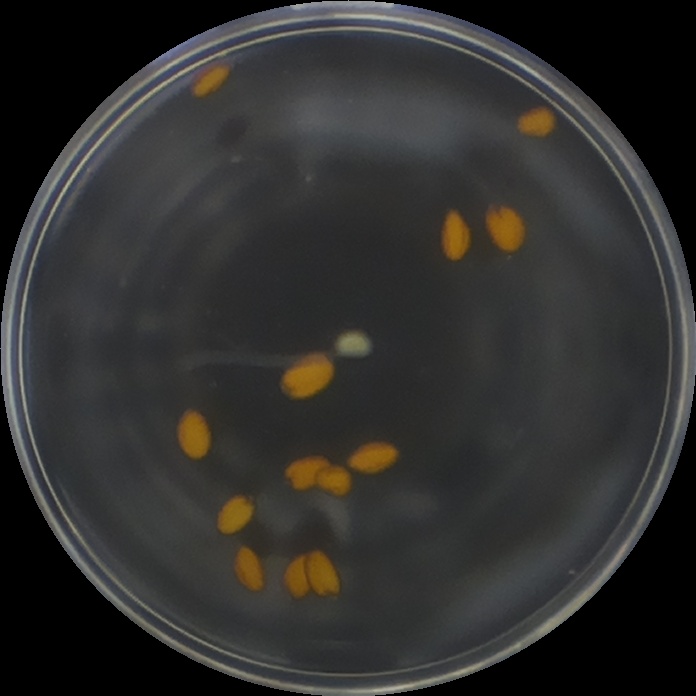

Supplement: Supplementary file 2 — Supplementary Information 2. [file 41598_2020_79115_MOESM2_ESM.zip › PictureOneWell/D5/50_584746]

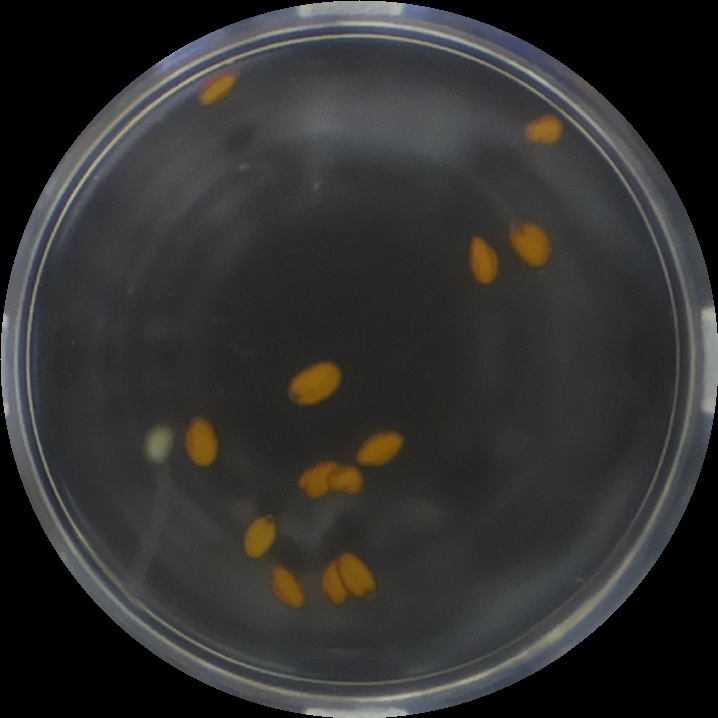

Supplement: Supplementary file 2 — Supplementary Information 2. [file 41598_2020_79115_MOESM2_ESM.zip › PictureOneWell/D5/26_580121]

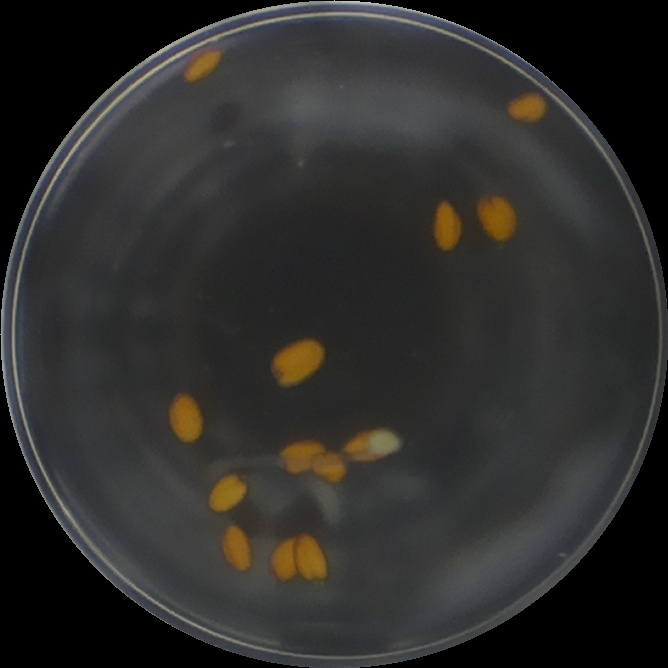

Supplement: Supplementary file 2 — Supplementary Information 2. [file 41598_2020_79115_MOESM2_ESM.zip › PictureOneWell/D5/67_587981]

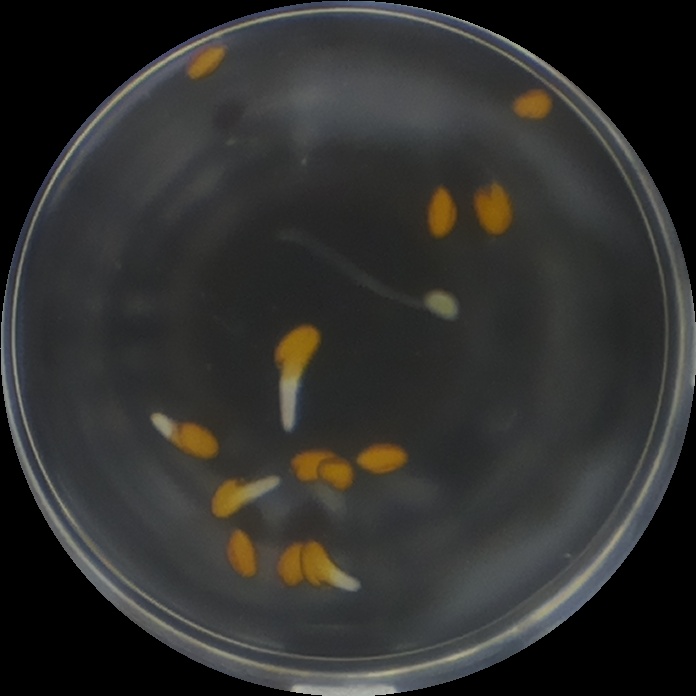

Supplement: Supplementary file 2 — Supplementary Information 2. [file 41598_2020_79115_MOESM2_ESM.zip › PictureOneWell/D5/112_596616]

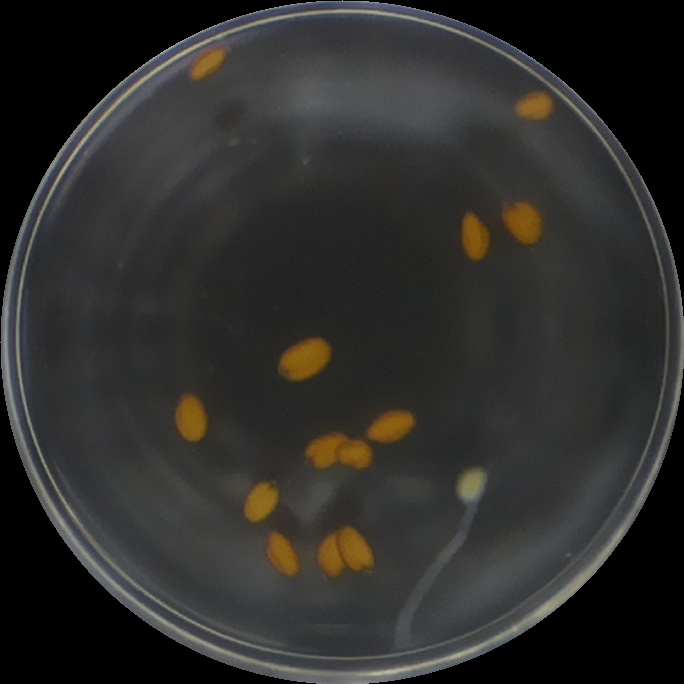

Supplement: Supplementary file 2 — Supplementary Information 2. [file 41598_2020_79115_MOESM2_ESM.zip › PictureOneWell/D5/39_582654]

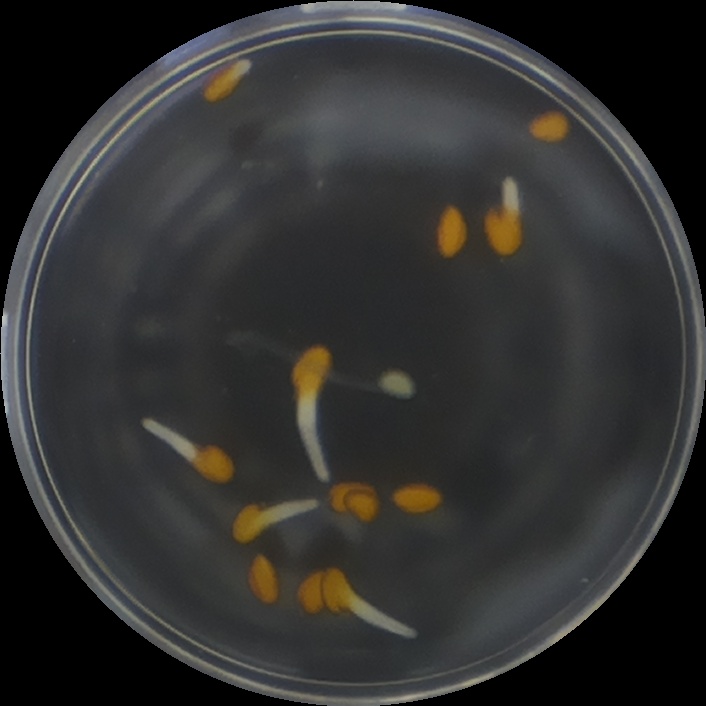

Supplement: Supplementary file 2 — Supplementary Information 2. [file 41598_2020_79115_MOESM2_ESM.zip › PictureOneWell/D5/128_599734]

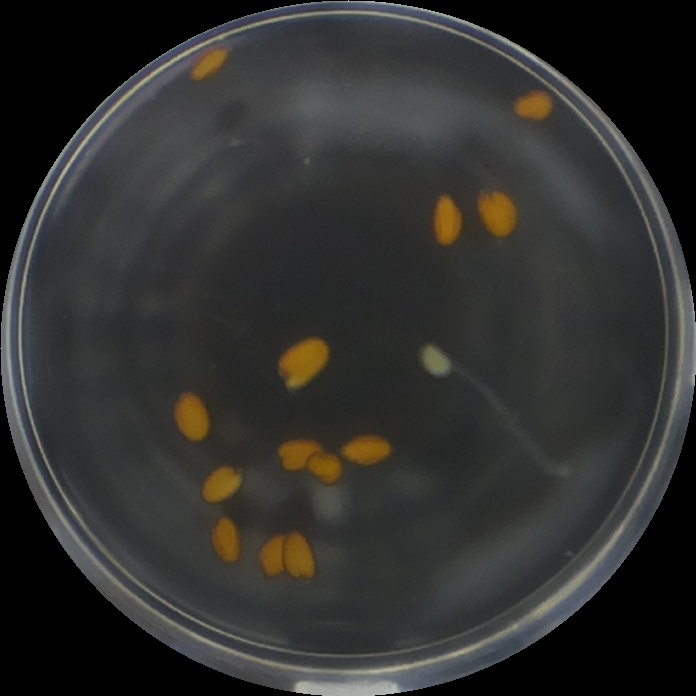

Supplement: Supplementary file 2 — Supplementary Information 2. [file 41598_2020_79115_MOESM2_ESM.zip › PictureOneWell/D5/72_588971]

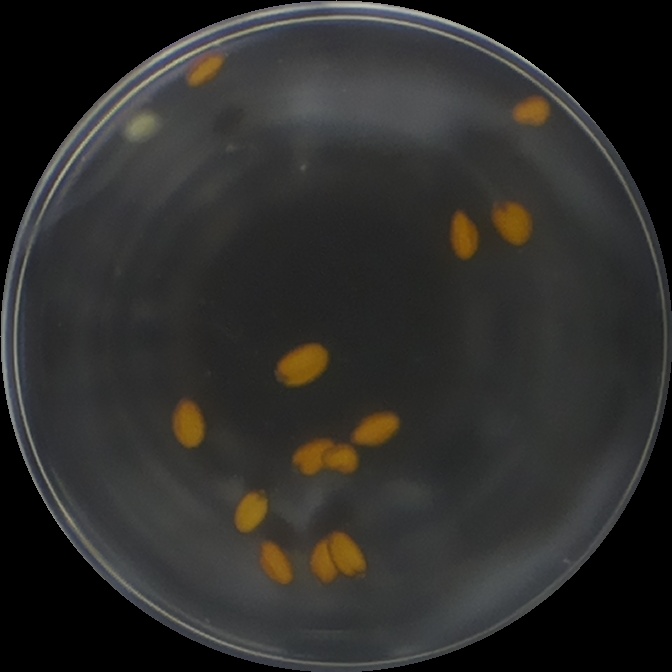

Supplement: Supplementary file 2 — Supplementary Information 2. [file 41598_2020_79115_MOESM2_ESM.zip › PictureOneWell/D5/32_581269]

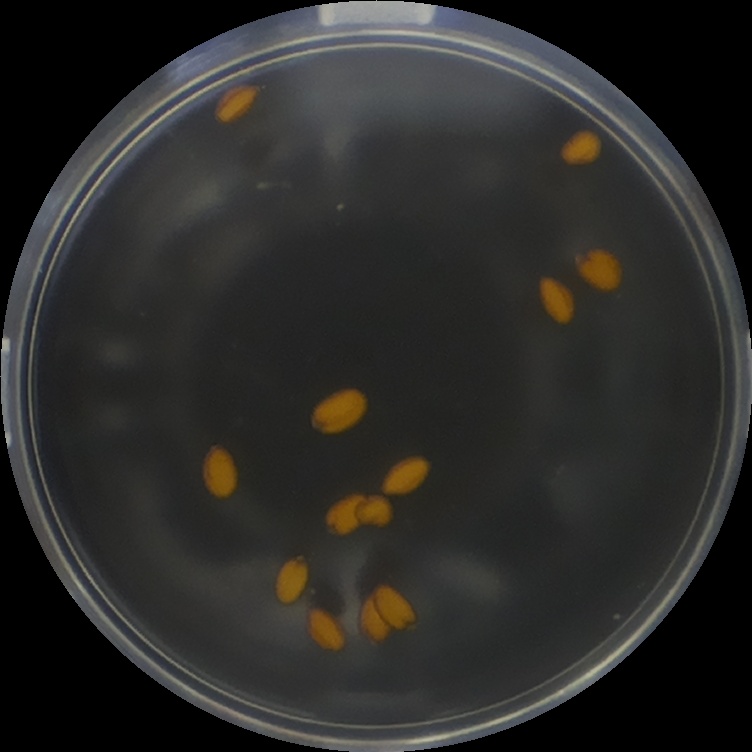

Supplement: Supplementary file 2 — Supplementary Information 2. [file 41598_2020_79115_MOESM2_ESM.zip › PictureOneWell/D5/7_576656]

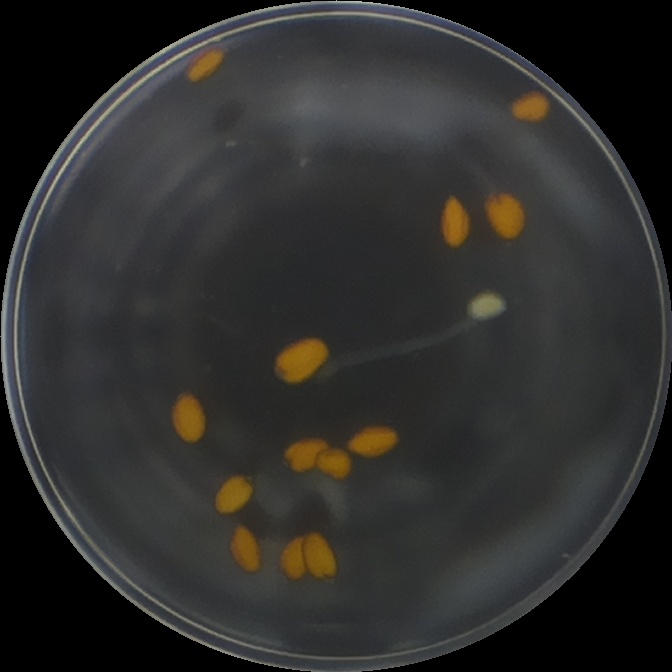

Supplement: Supplementary file 2 — Supplementary Information 2. [file 41598_2020_79115_MOESM2_ESM.zip › PictureOneWell/D5/60_586704]

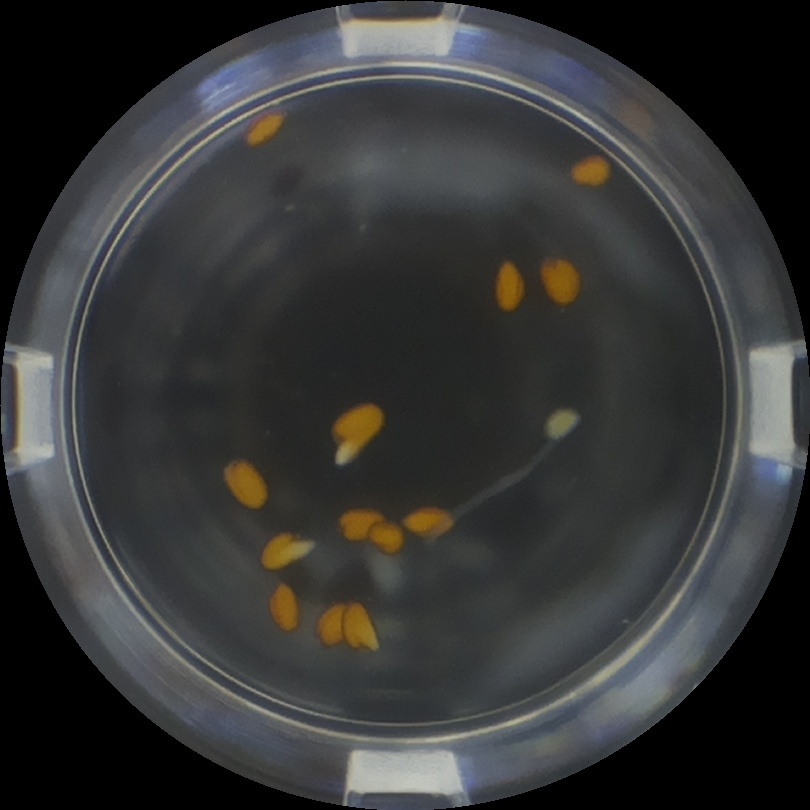

Supplement: Supplementary file 2 — Supplementary Information 2. [file 41598_2020_79115_MOESM2_ESM.zip › PictureOneWell/D5/89_592215]

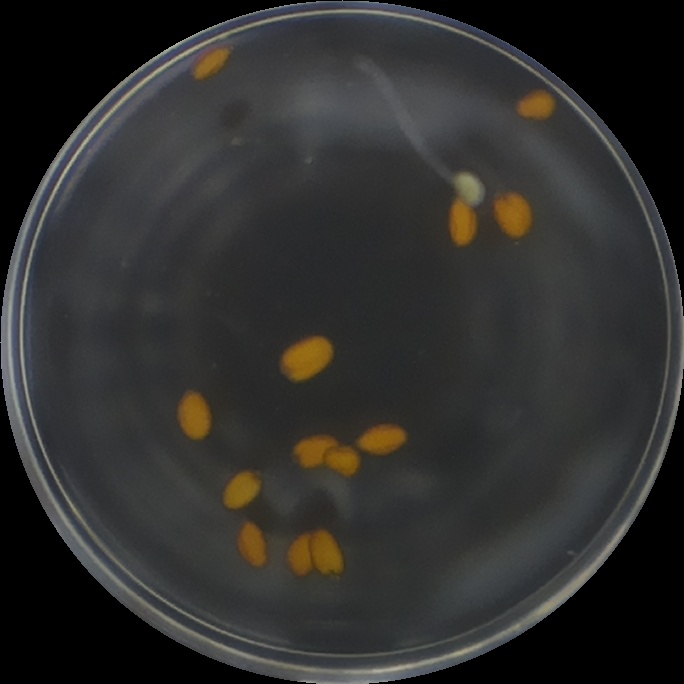

Supplement: Supplementary file 2 — Supplementary Information 2. [file 41598_2020_79115_MOESM2_ESM.zip › PictureOneWell/D5/62_587070]

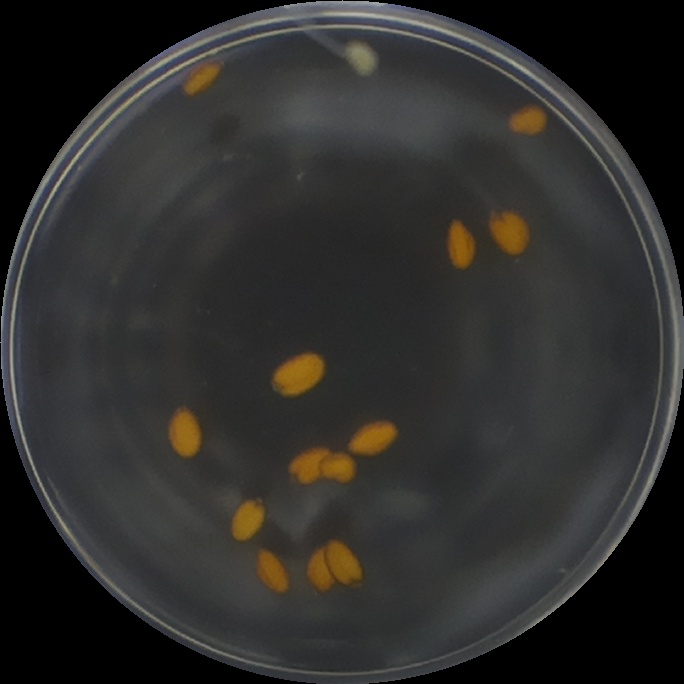

Supplement: Supplementary file 2 — Supplementary Information 2. [file 41598_2020_79115_MOESM2_ESM.zip › PictureOneWell/D5/33_581522]

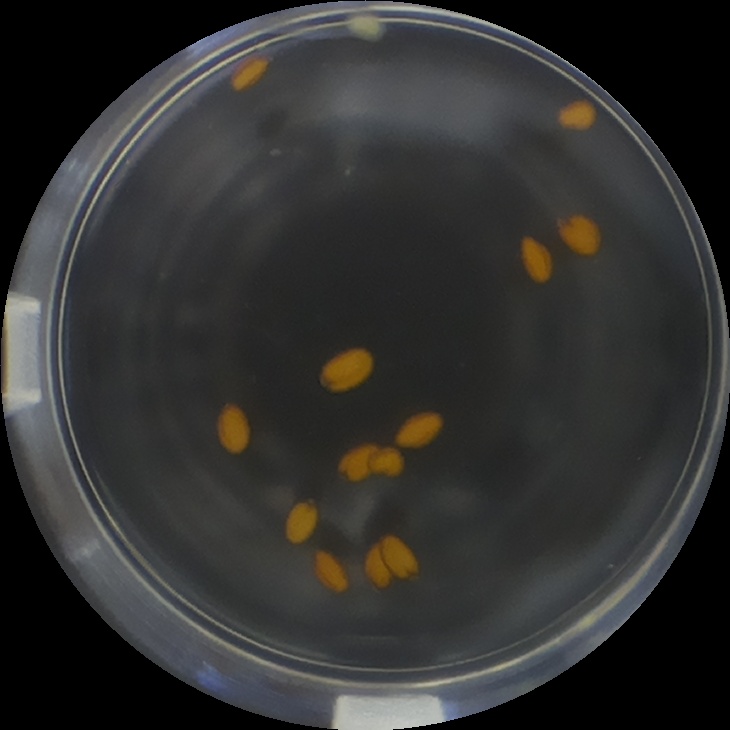

Supplement: Supplementary file 2 — Supplementary Information 2. [file 41598_2020_79115_MOESM2_ESM.zip › PictureOneWell/D5/20_579025]

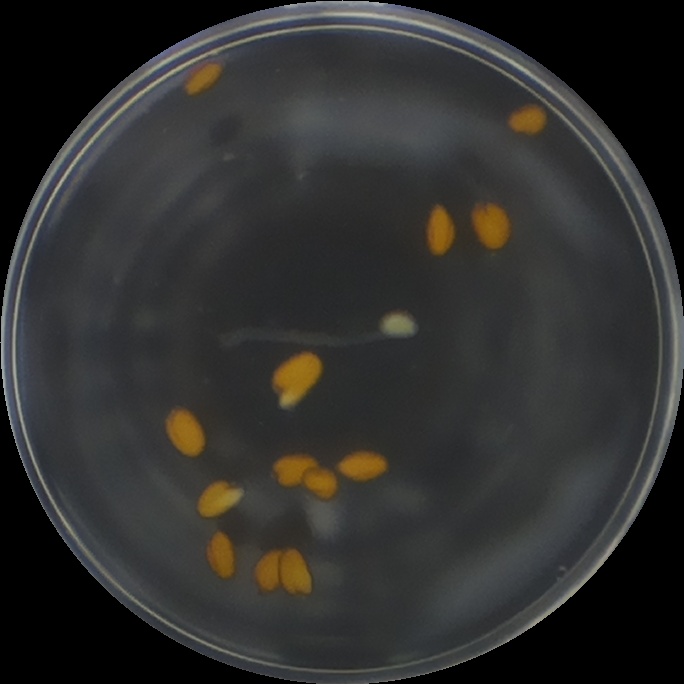

Supplement: Supplementary file 2 — Supplementary Information 2. [file 41598_2020_79115_MOESM2_ESM.zip › PictureOneWell/D5/79_590351]

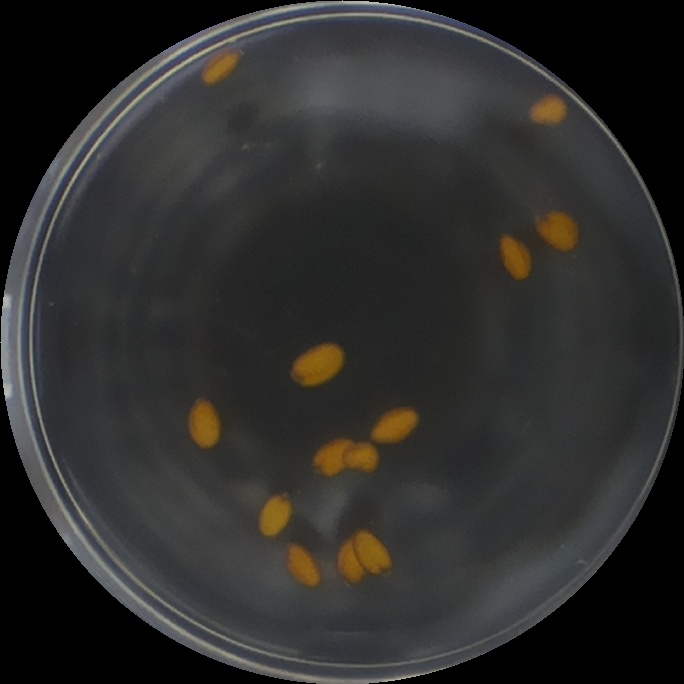

Supplement: Supplementary file 2 — Supplementary Information 2. [file 41598_2020_79115_MOESM2_ESM.zip › PictureOneWell/D5/18_578574]

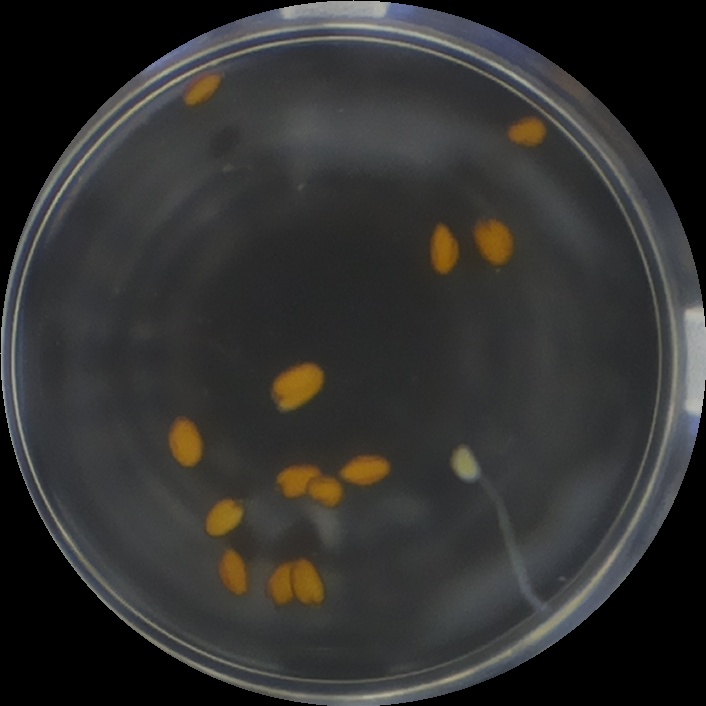

Supplement: Supplementary file 2 — Supplementary Information 2. [file 41598_2020_79115_MOESM2_ESM.zip › PictureOneWell/D5/68_588197]

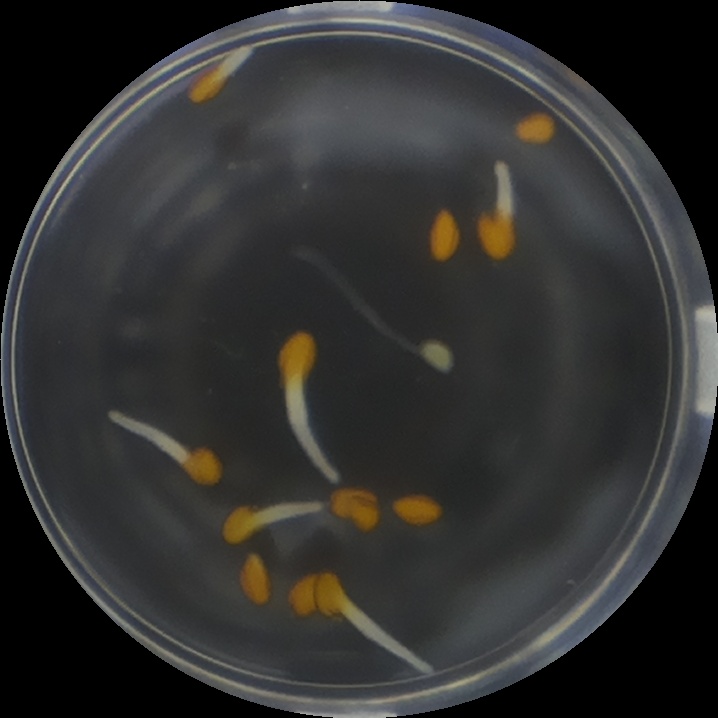

Supplement: Supplementary file 2 — Supplementary Information 2. [file 41598_2020_79115_MOESM2_ESM.zip › PictureOneWell/D5/141_602225]

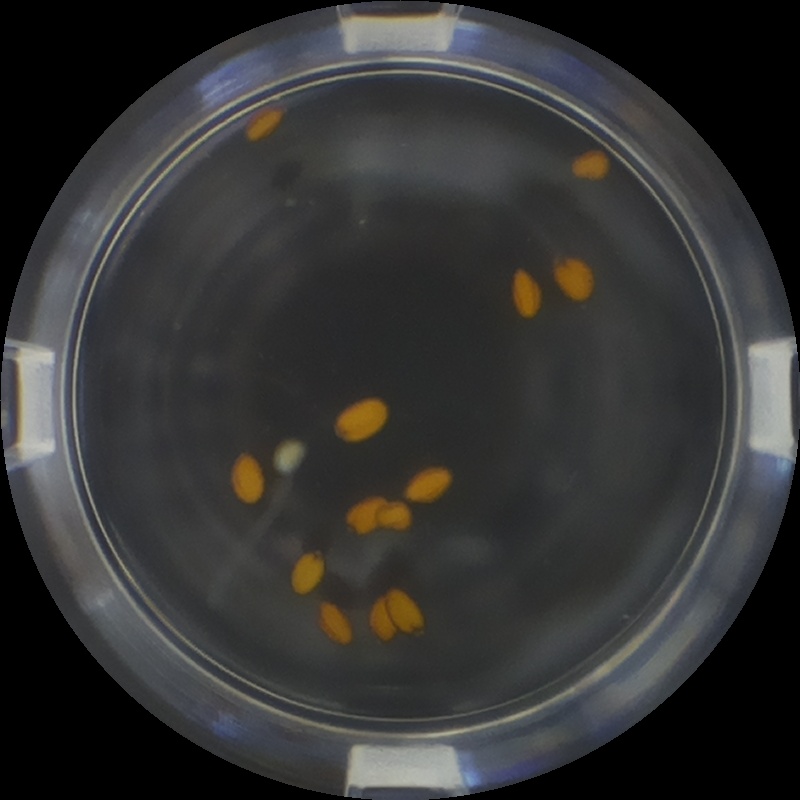

Supplement: Supplementary file 2 — Supplementary Information 2. [file 41598_2020_79115_MOESM2_ESM.zip › PictureOneWell/D5/28_580512]

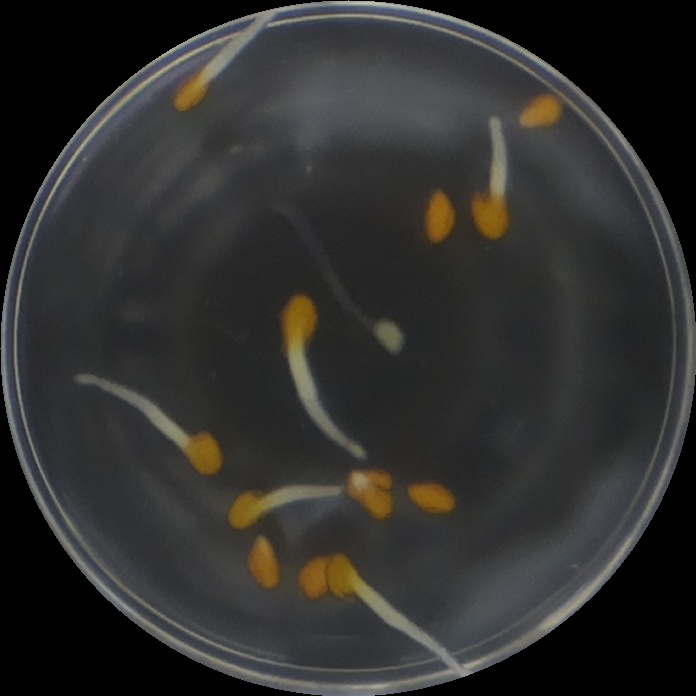

Supplement: Supplementary file 2 — Supplementary Information 2. [file 41598_2020_79115_MOESM2_ESM.zip › PictureOneWell/D5/153_604663]

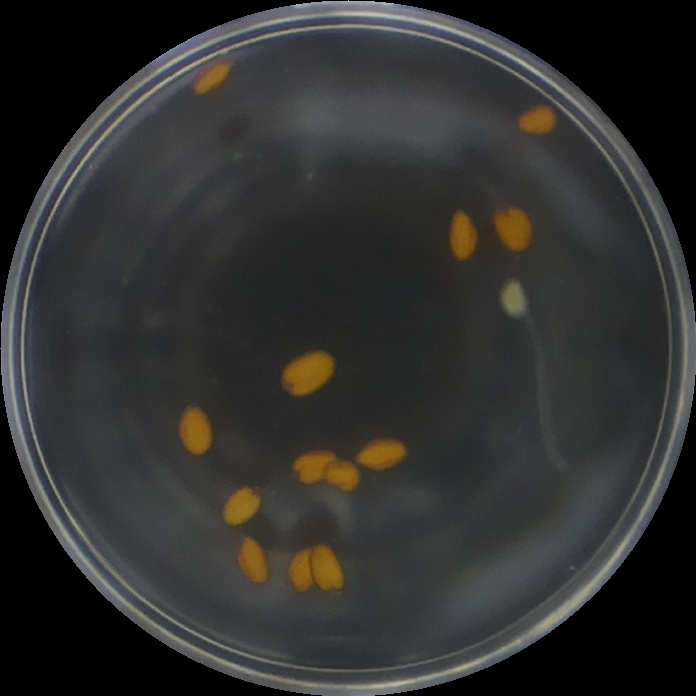

Supplement: Supplementary file 2 — Supplementary Information 2. [file 41598_2020_79115_MOESM2_ESM.zip › PictureOneWell/D5/61_586915]

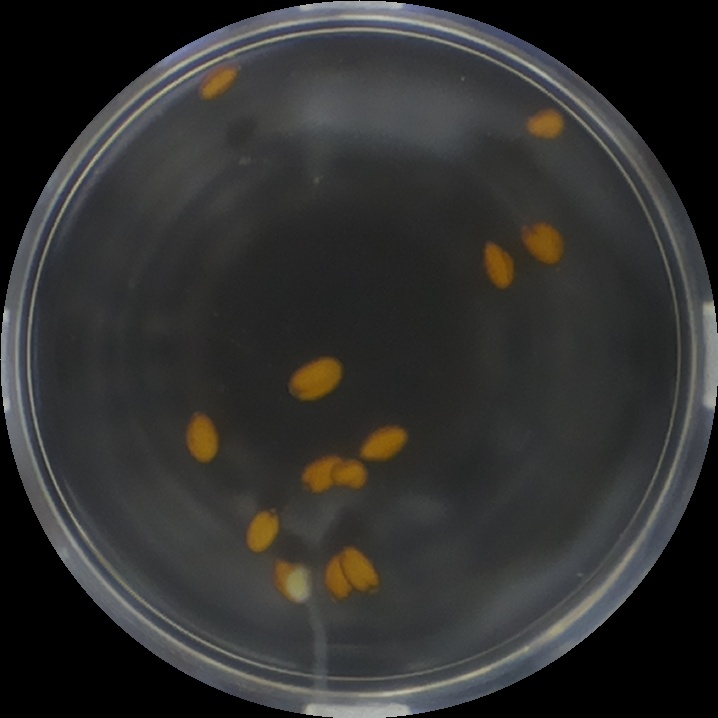

Supplement: Supplementary file 2 — Supplementary Information 2. [file 41598_2020_79115_MOESM2_ESM.zip › PictureOneWell/D5/22_579342]

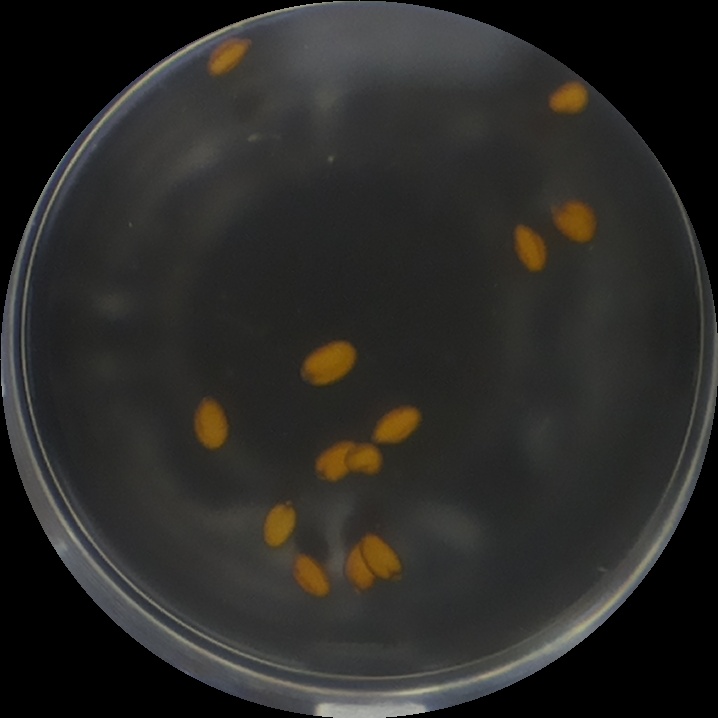

Supplement: Supplementary file 2 — Supplementary Information 2. [file 41598_2020_79115_MOESM2_ESM.zip › PictureOneWell/D5/10_577305]

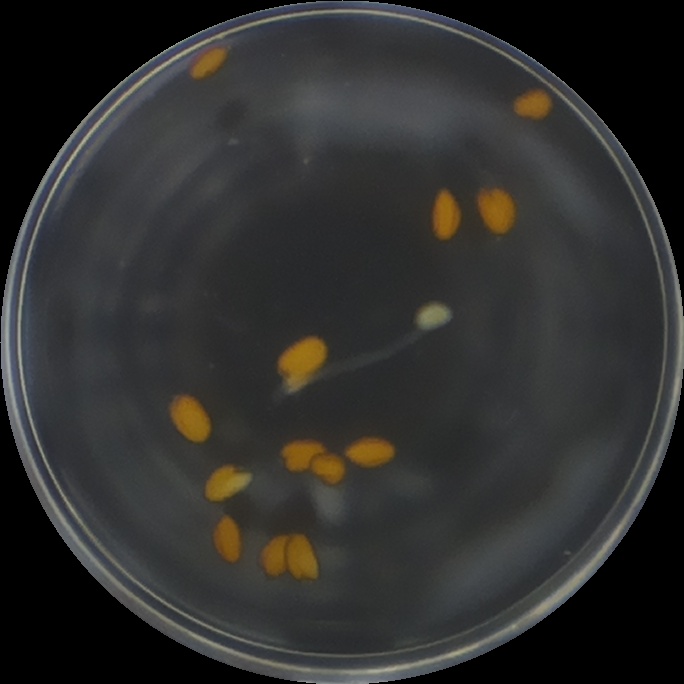

Supplement: Supplementary file 2 — Supplementary Information 2. [file 41598_2020_79115_MOESM2_ESM.zip › PictureOneWell/D5/80_590561]

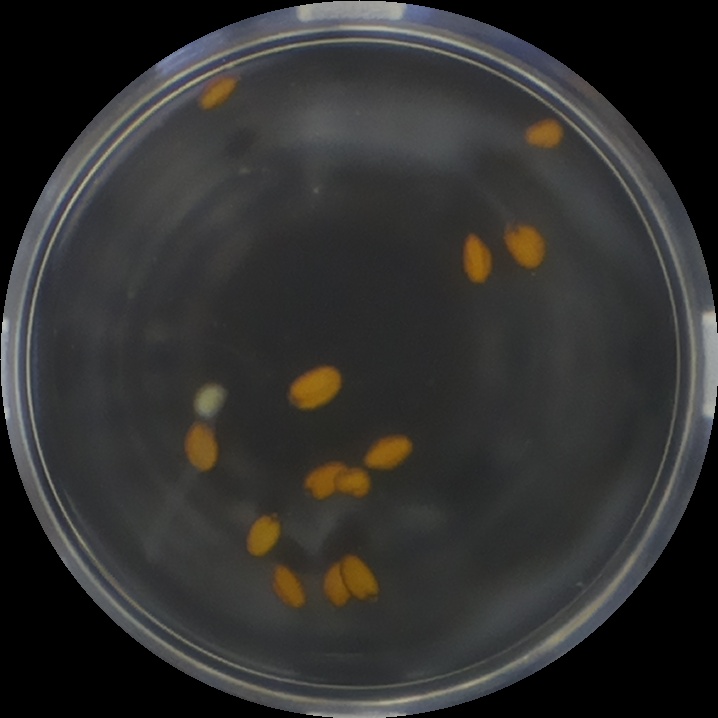

Supplement: Supplementary file 2 — Supplementary Information 2. [file 41598_2020_79115_MOESM2_ESM.zip › PictureOneWell/D5/31_581113]

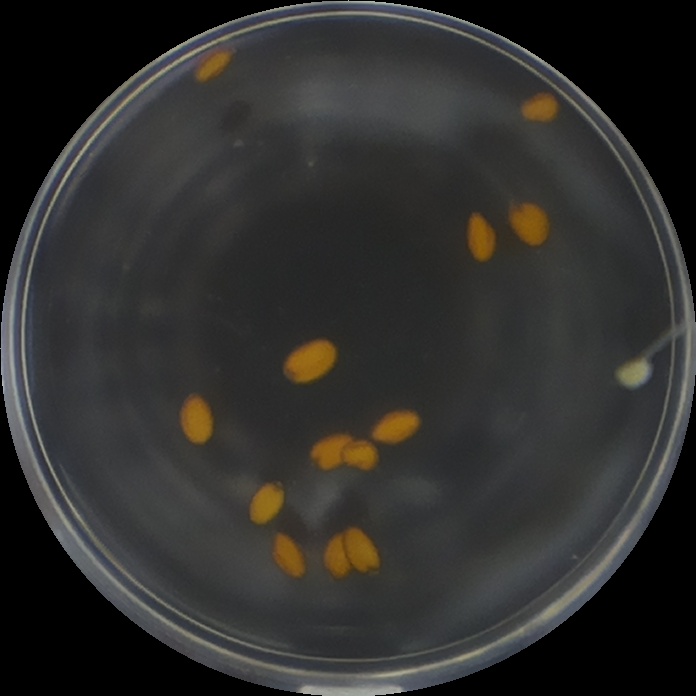

Supplement: Supplementary file 2 — Supplementary Information 2. [file 41598_2020_79115_MOESM2_ESM.zip › PictureOneWell/D5/38_582428]

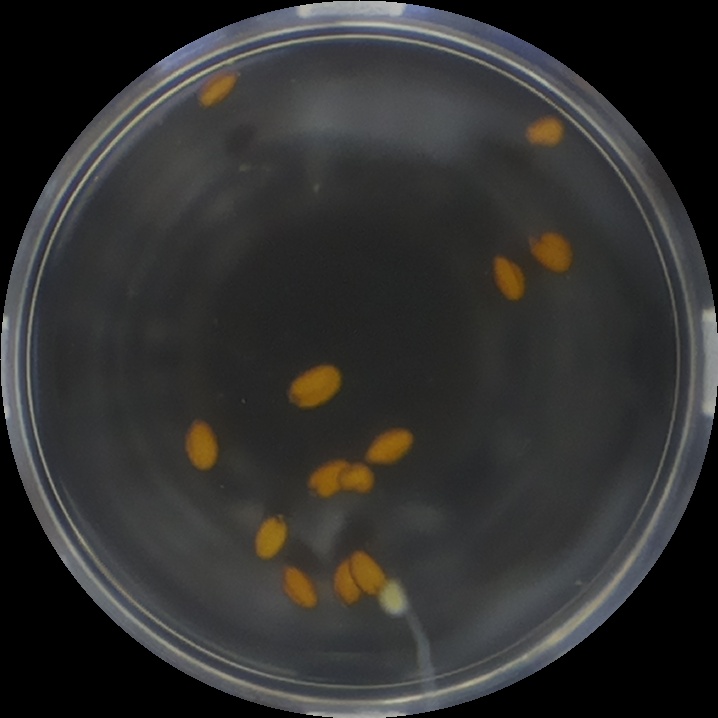

Supplement: Supplementary file 2 — Supplementary Information 2. [file 41598_2020_79115_MOESM2_ESM.zip › PictureOneWell/D5/19_578819]

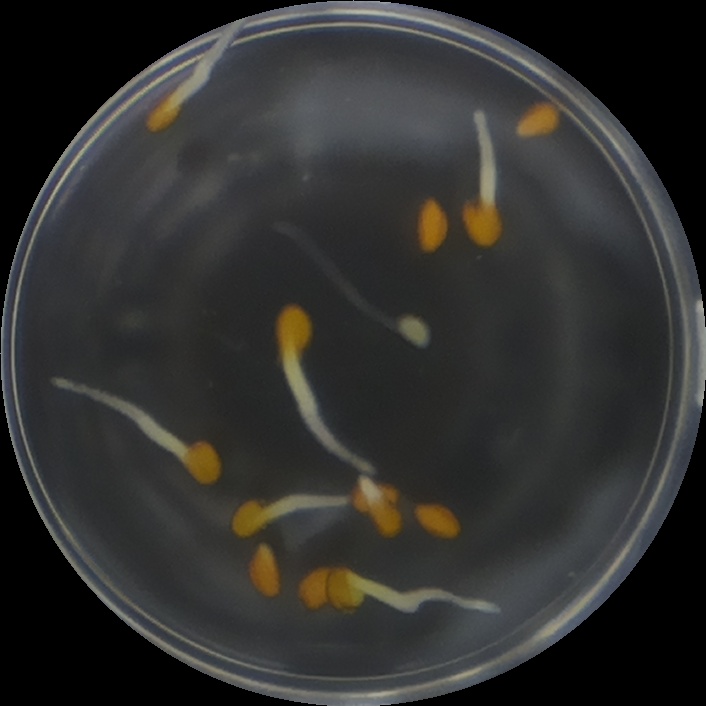

Supplement: Supplementary file 2 — Supplementary Information 2. [file 41598_2020_79115_MOESM2_ESM.zip › PictureOneWell/D5/160_606050]

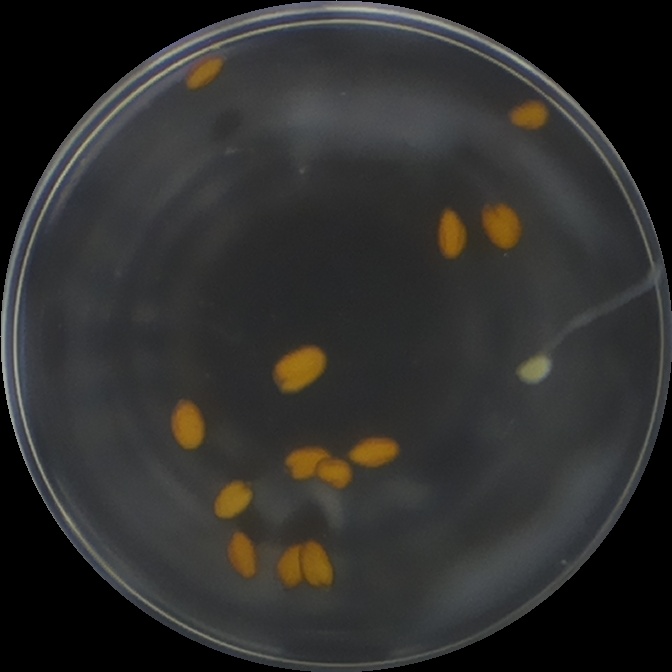

Supplement: Supplementary file 2 — Supplementary Information 2. [file 41598_2020_79115_MOESM2_ESM.zip › PictureOneWell/D5/66_587788]

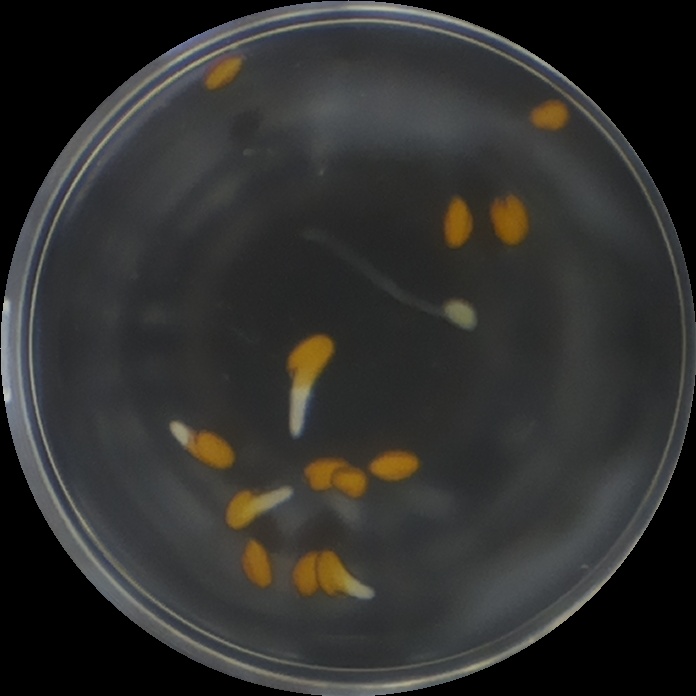

Supplement: Supplementary file 2 — Supplementary Information 2. [file 41598_2020_79115_MOESM2_ESM.zip › PictureOneWell/D5/111_596494]

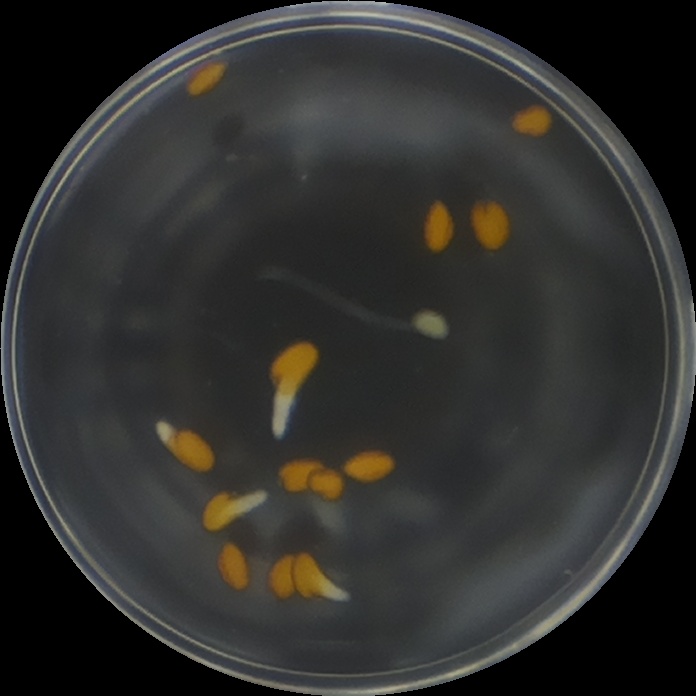

Supplement: Supplementary file 2 — Supplementary Information 2. [file 41598_2020_79115_MOESM2_ESM.zip › PictureOneWell/D5/108_595892]

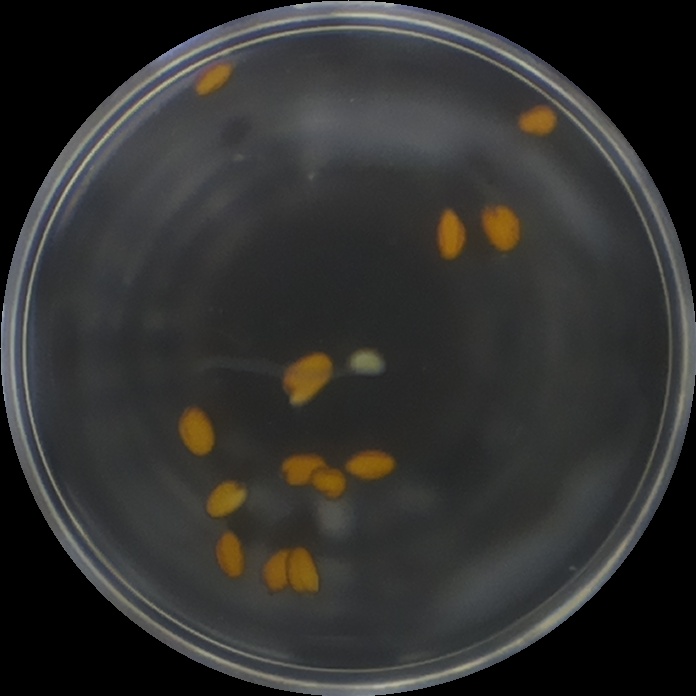

Supplement: Supplementary file 2 — Supplementary Information 2. [file 41598_2020_79115_MOESM2_ESM.zip › PictureOneWell/D5/74_589353]

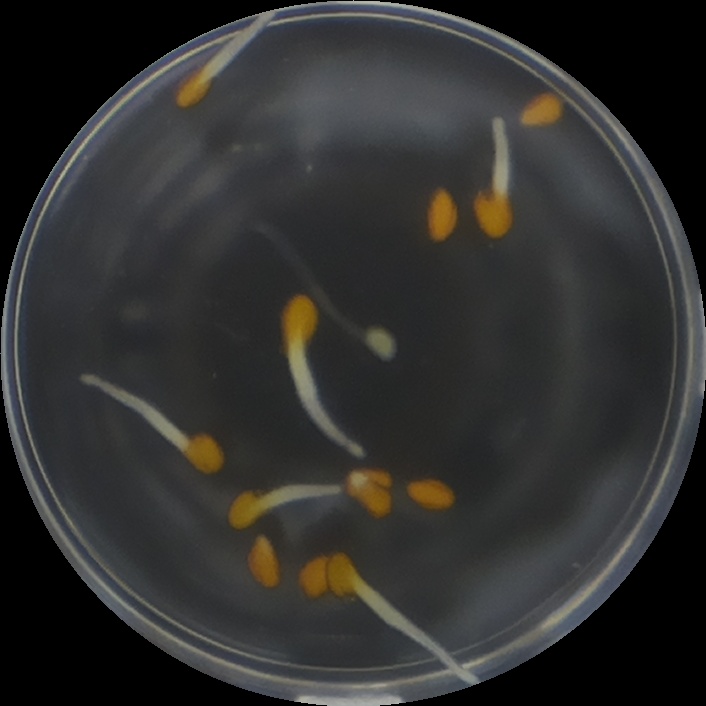

Supplement: Supplementary file 2 — Supplementary Information 2. [file 41598_2020_79115_MOESM2_ESM.zip › PictureOneWell/D5/152_604425]

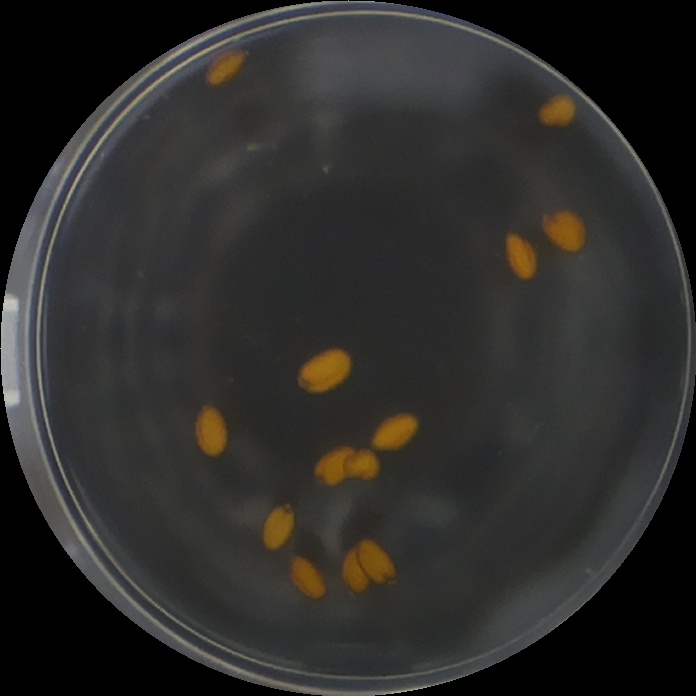

Supplement: Supplementary file 2 — Supplementary Information 2. [file 41598_2020_79115_MOESM2_ESM.zip › PictureOneWell/D5/12_577620]

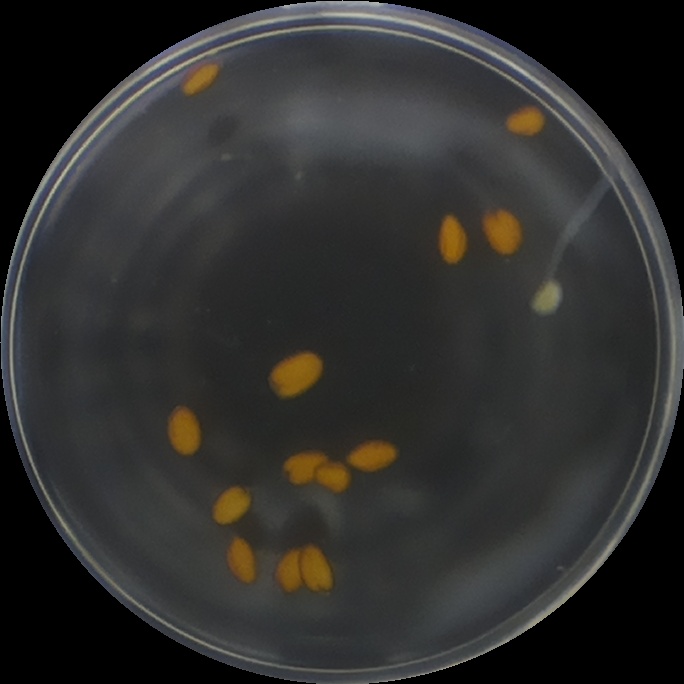

Supplement: Supplementary file 2 — Supplementary Information 2. [file 41598_2020_79115_MOESM2_ESM.zip › PictureOneWell/D5/64_587433]

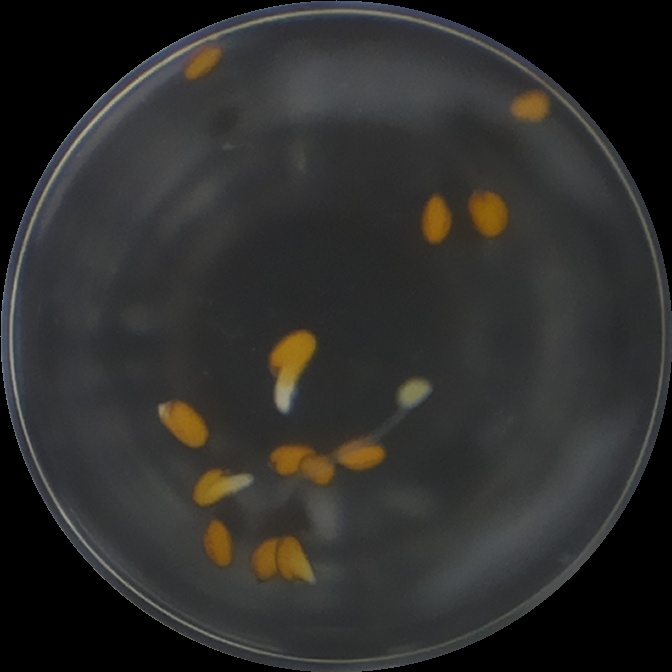

Supplement: Supplementary file 2 — Supplementary Information 2. [file 41598_2020_79115_MOESM2_ESM.zip › PictureOneWell/D5/100_594318]

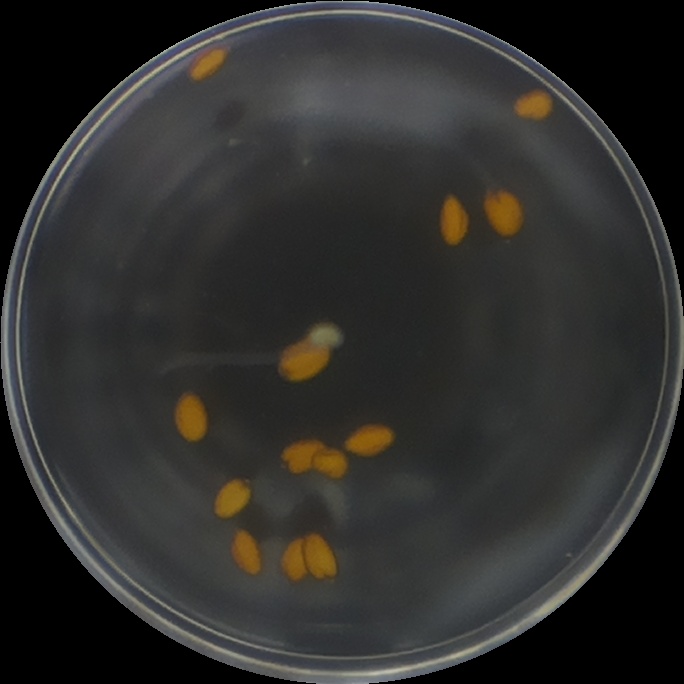

Supplement: Supplementary file 2 — Supplementary Information 2. [file 41598_2020_79115_MOESM2_ESM.zip › PictureOneWell/D5/48_584401]

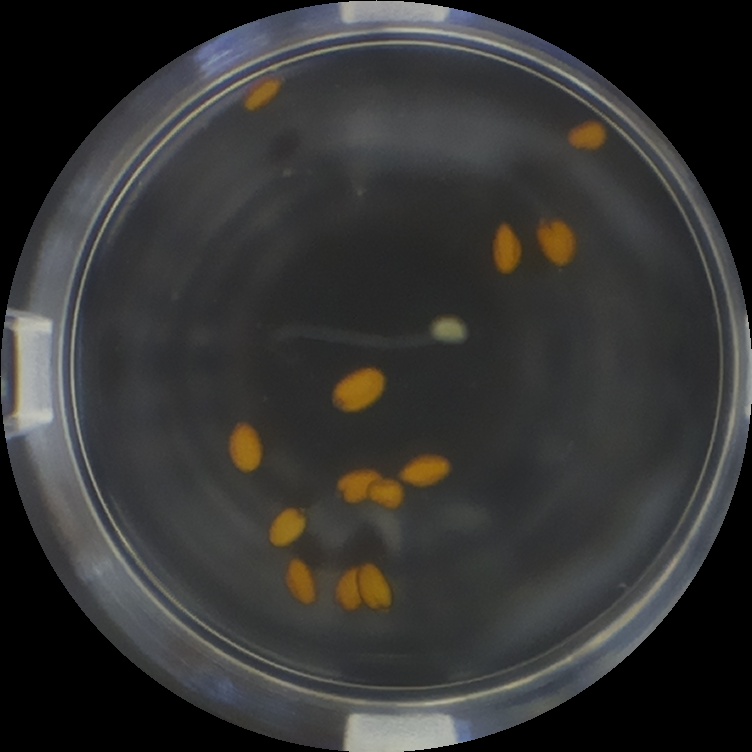

Supplement: Supplementary file 2 — Supplementary Information 2. [file 41598_2020_79115_MOESM2_ESM.zip › PictureOneWell/D5/53_585299]

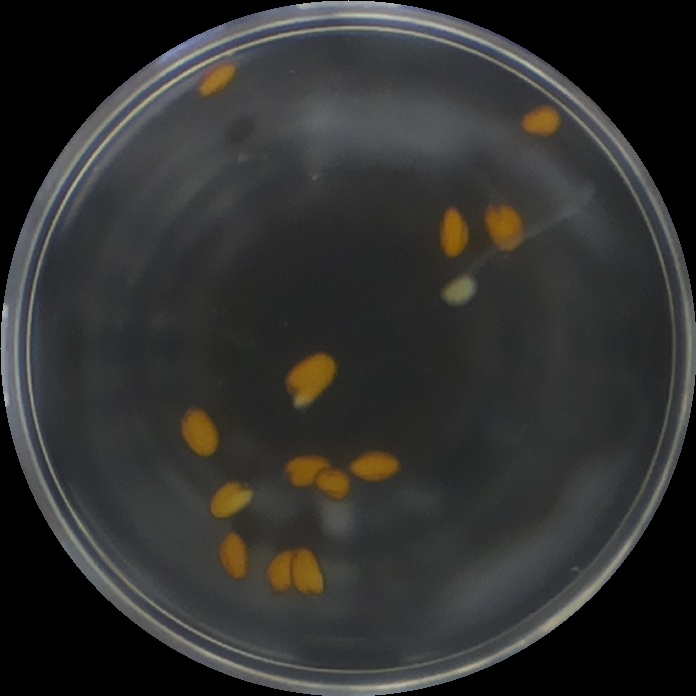

Supplement: Supplementary file 2 — Supplementary Information 2. [file 41598_2020_79115_MOESM2_ESM.zip › PictureOneWell/D5/77_589971]
