# Supplementary figures and images for: ScreenSeed as a novel high throughput seed germination phenotyping method (part 1 of 2)
Source: Sci Rep. 2021 Jan 14;11:1404. doi: 10.1038/s41598-020-79115-2 (PMC7809209; doi:10.1038/s41598-020-79115-2)

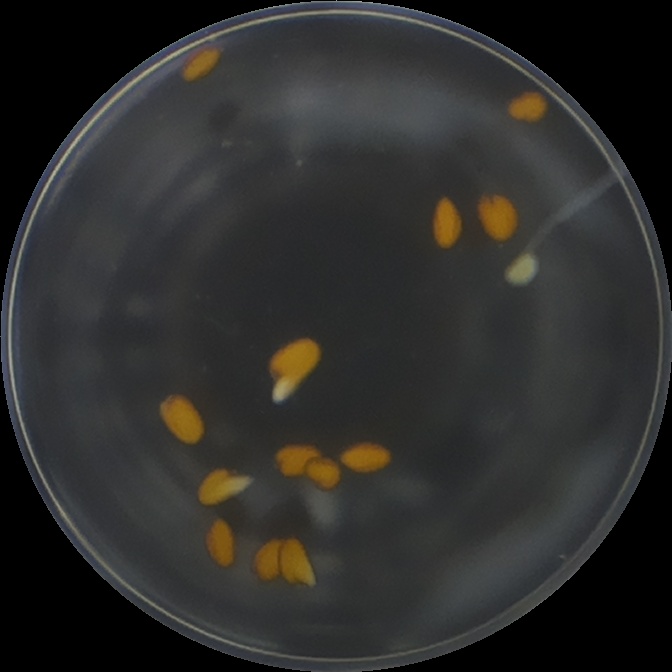

Supplement: Supplementary file 2 — Supplementary Information 2. [file 41598_2020_79115_MOESM2_ESM.zip › PictureOneWell/D5/90_592476]

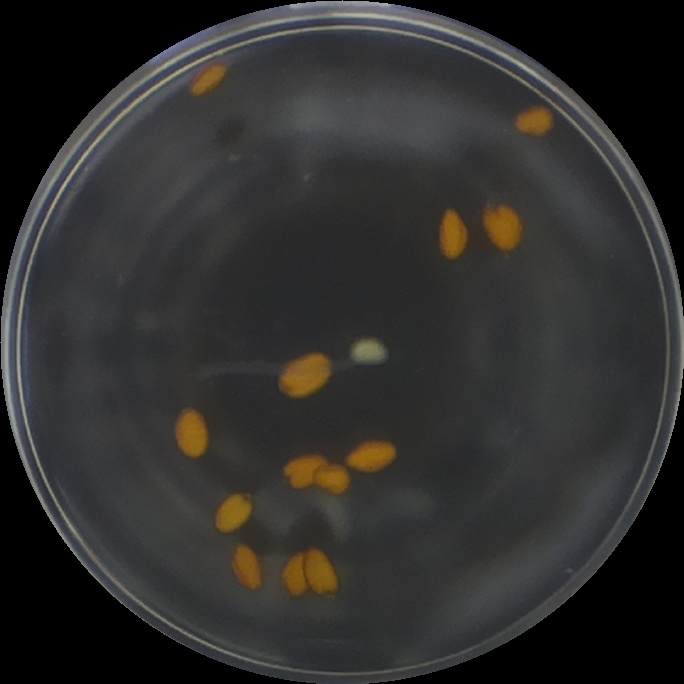

Supplement: Supplementary file 2 — Supplementary Information 2. [file 41598_2020_79115_MOESM2_ESM.zip › PictureOneWell/D5/52_585176]

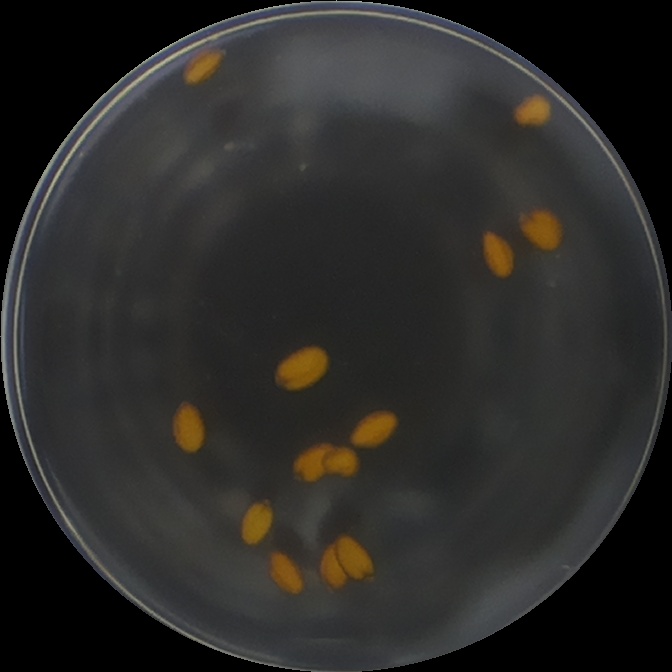

Supplement: Supplementary file 2 — Supplementary Information 2. [file 41598_2020_79115_MOESM2_ESM.zip › PictureOneWell/D5/13_577844]

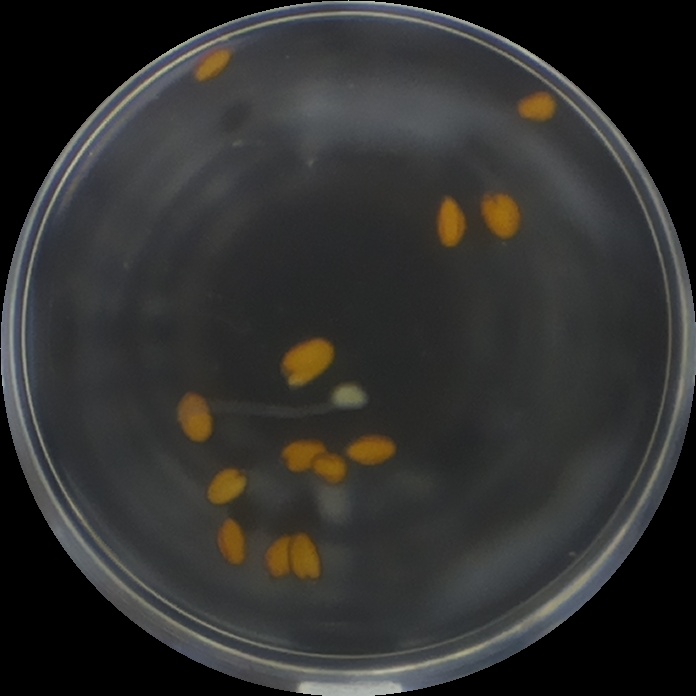

Supplement: Supplementary file 2 — Supplementary Information 2. [file 41598_2020_79115_MOESM2_ESM.zip › PictureOneWell/D5/70_588568]

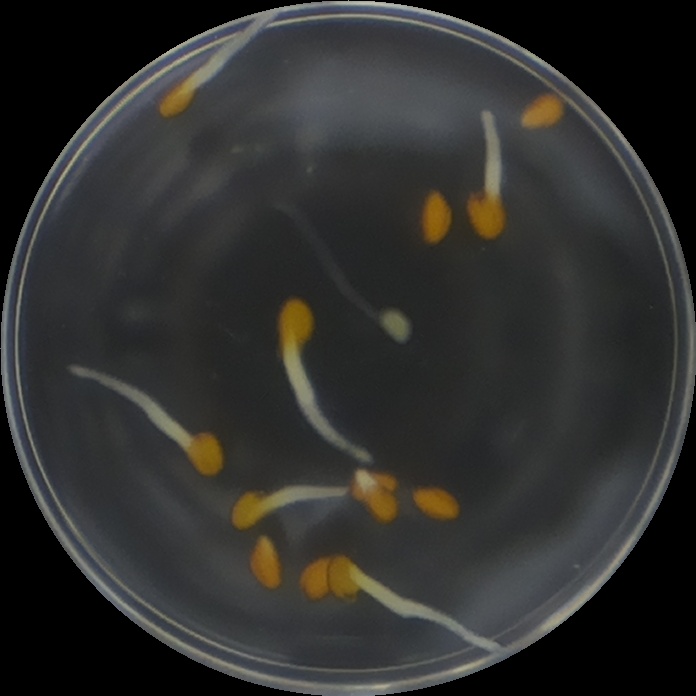

Supplement: Supplementary file 2 — Supplementary Information 2. [file 41598_2020_79115_MOESM2_ESM.zip › PictureOneWell/D5/156_605209]

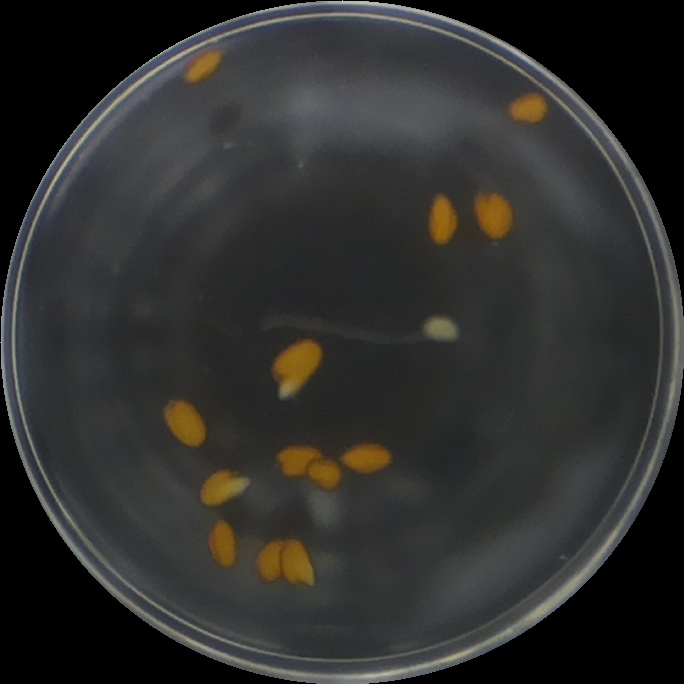

Supplement: Supplementary file 2 — Supplementary Information 2. [file 41598_2020_79115_MOESM2_ESM.zip › PictureOneWell/D5/83_591083]

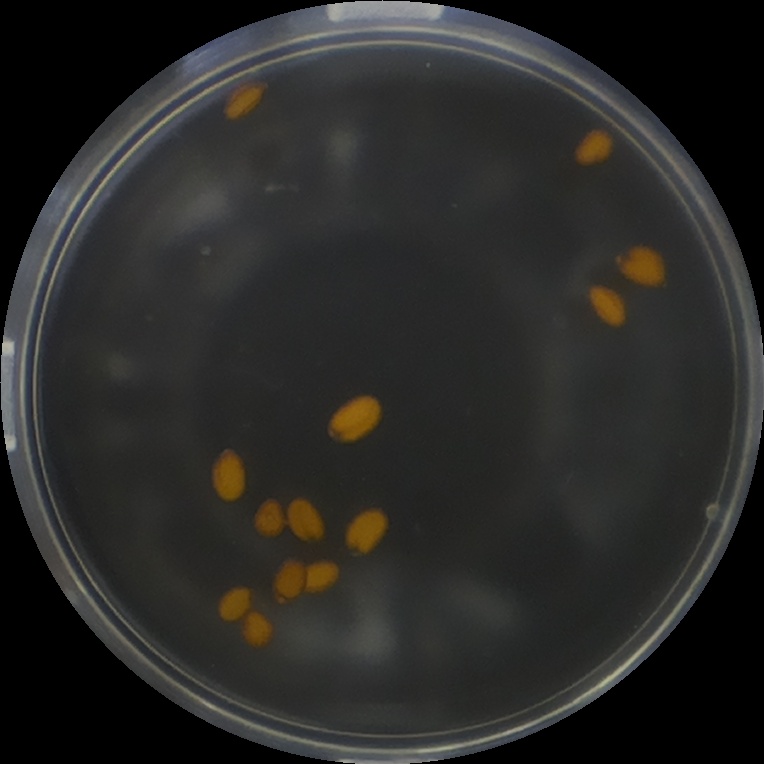

Supplement: Supplementary file 2 — Supplementary Information 2. [file 41598_2020_79115_MOESM2_ESM.zip › PictureOneWell/D5/0_575350]

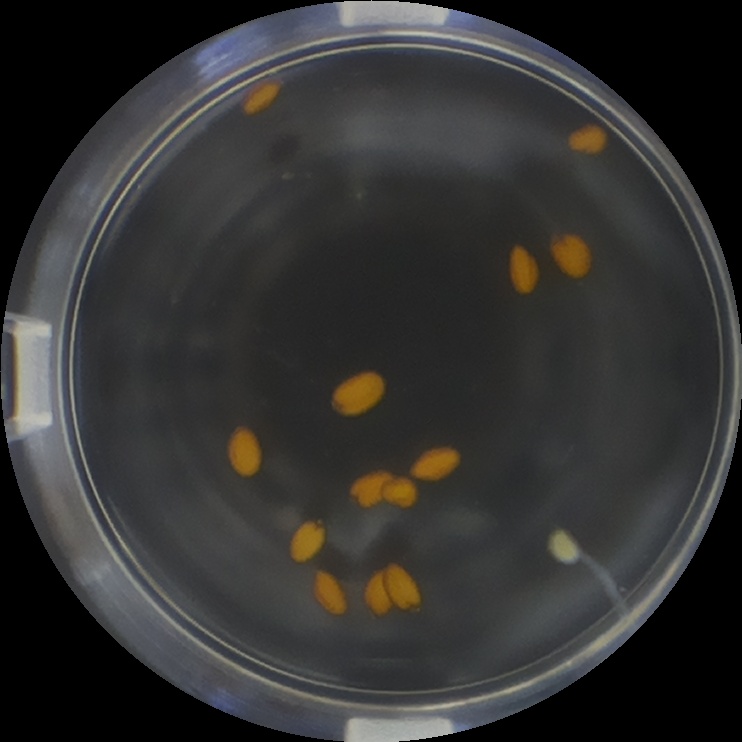

Supplement: Supplementary file 2 — Supplementary Information 2. [file 41598_2020_79115_MOESM2_ESM.zip › PictureOneWell/D5/41_583070]

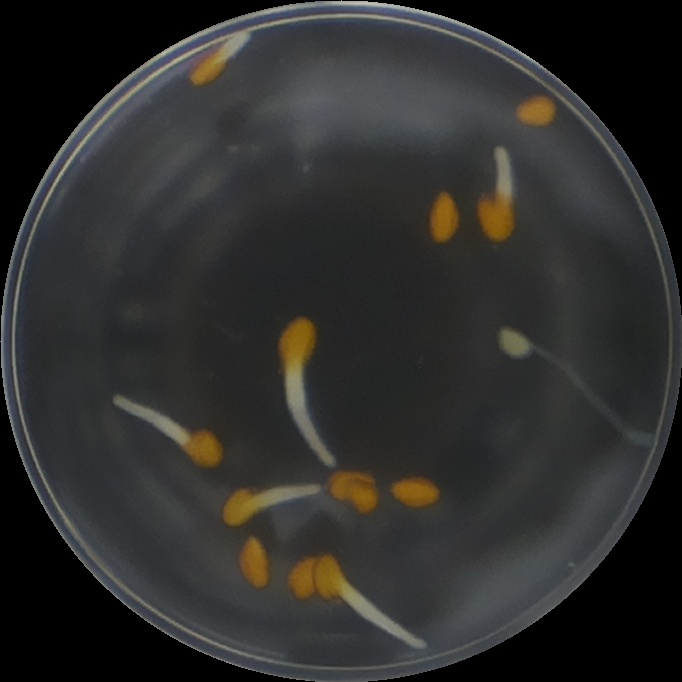

Supplement: Supplementary file 2 — Supplementary Information 2. [file 41598_2020_79115_MOESM2_ESM.zip › PictureOneWell/D5/139_601819]

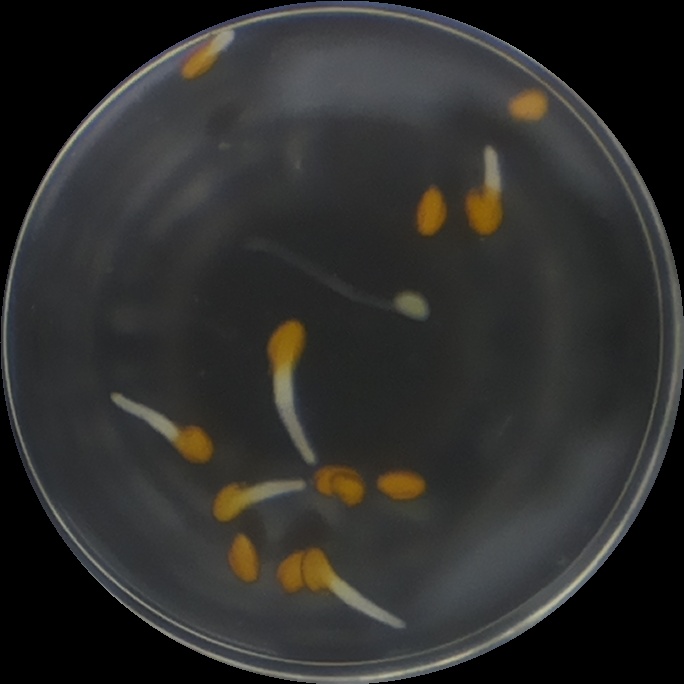

Supplement: Supplementary file 2 — Supplementary Information 2. [file 41598_2020_79115_MOESM2_ESM.zip › PictureOneWell/D5/134_600917]

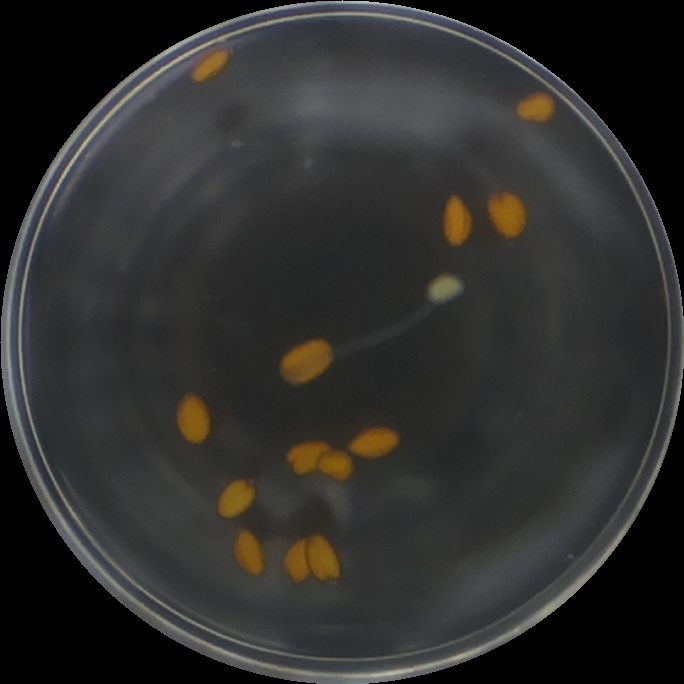

Supplement: Supplementary file 2 — Supplementary Information 2. [file 41598_2020_79115_MOESM2_ESM.zip › PictureOneWell/D5/56_585888]

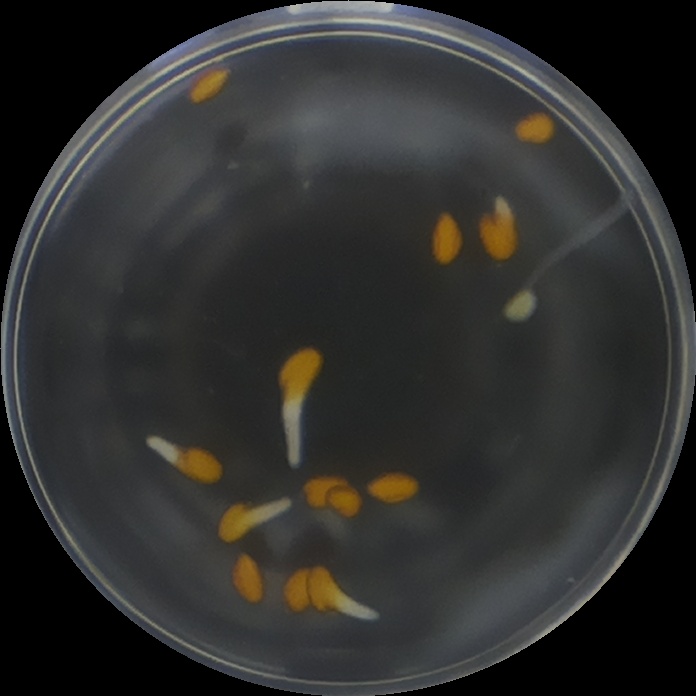

Supplement: Supplementary file 2 — Supplementary Information 2. [file 41598_2020_79115_MOESM2_ESM.zip › PictureOneWell/D5/117_597619]

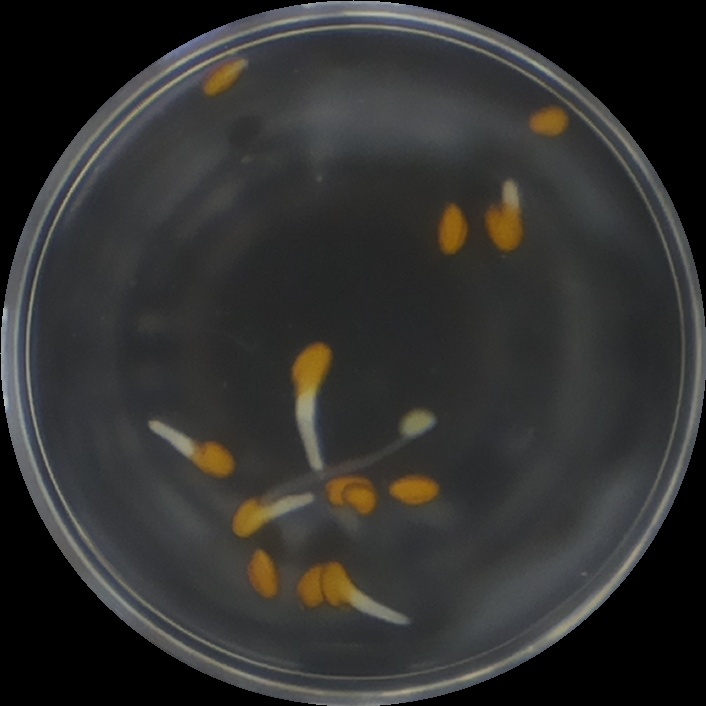

Supplement: Supplementary file 2 — Supplementary Information 2. [file 41598_2020_79115_MOESM2_ESM.zip › PictureOneWell/D5/124_598943]

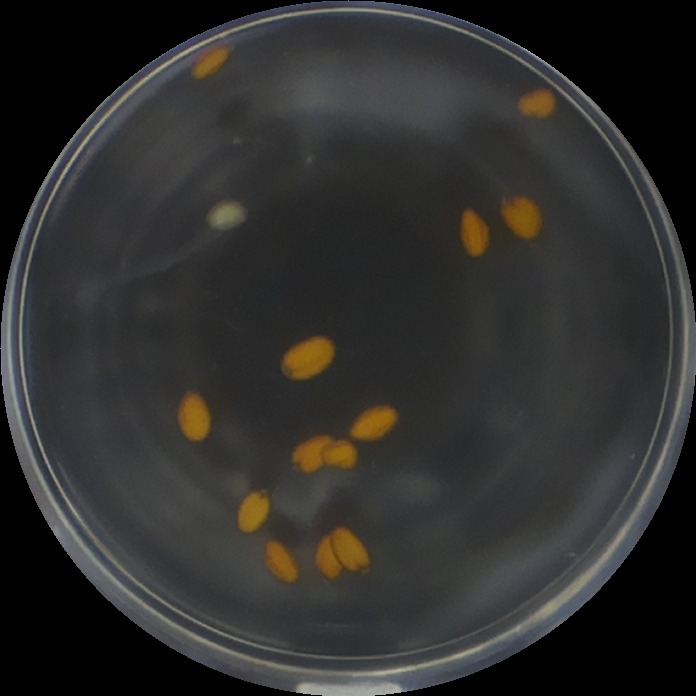

Supplement: Supplementary file 2 — Supplementary Information 2. [file 41598_2020_79115_MOESM2_ESM.zip › PictureOneWell/D5/27_580309]

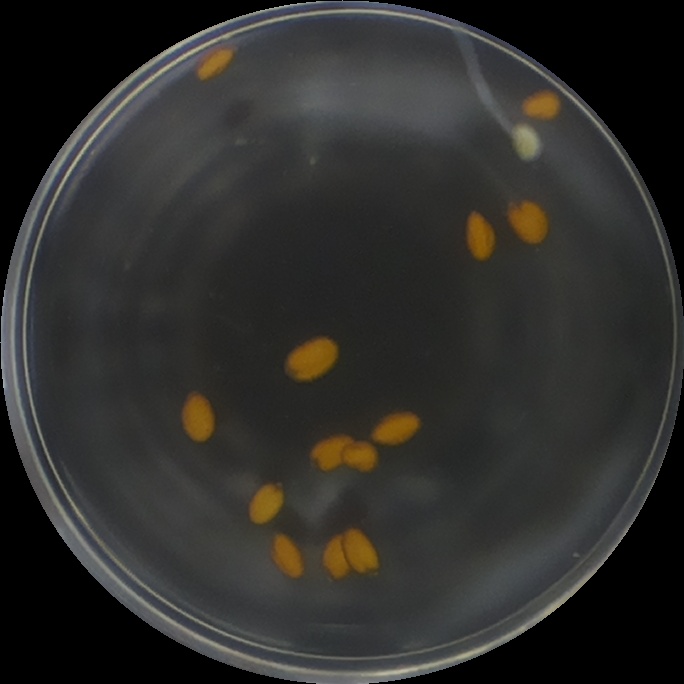

Supplement: Supplementary file 2 — Supplementary Information 2. [file 41598_2020_79115_MOESM2_ESM.zip › PictureOneWell/D5/40_582825]

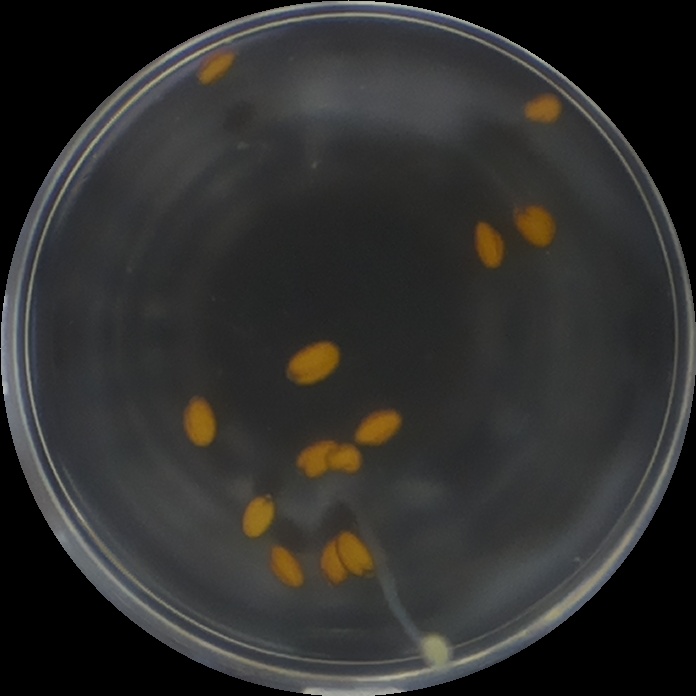

Supplement: Supplementary file 2 — Supplementary Information 2. [file 41598_2020_79115_MOESM2_ESM.zip › PictureOneWell/D5/24_579725]

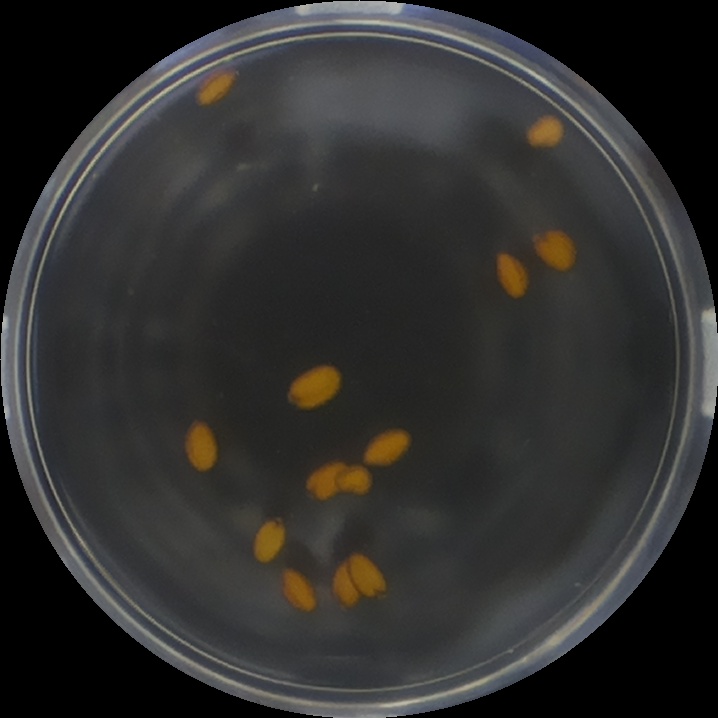

Supplement: Supplementary file 2 — Supplementary Information 2. [file 41598_2020_79115_MOESM2_ESM.zip › PictureOneWell/D5/14_578074]

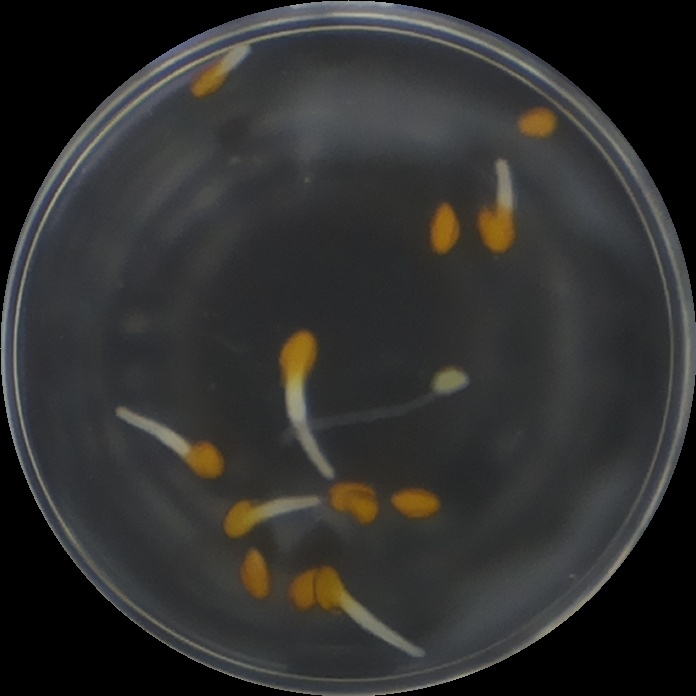

Supplement: Supplementary file 2 — Supplementary Information 2. [file 41598_2020_79115_MOESM2_ESM.zip › PictureOneWell/D5/138_601604]

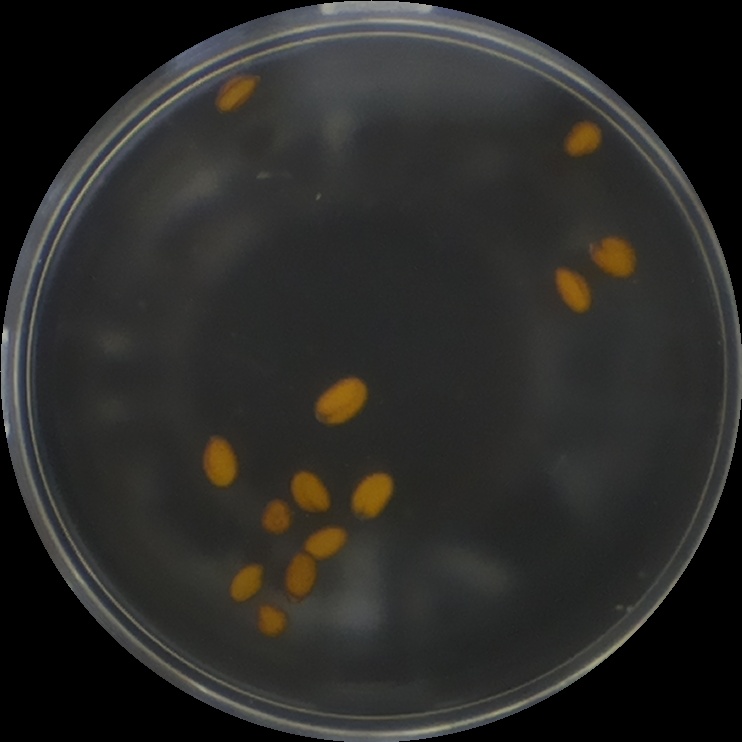

Supplement: Supplementary file 2 — Supplementary Information 2. [file 41598_2020_79115_MOESM2_ESM.zip › PictureOneWell/D5/4_576114]

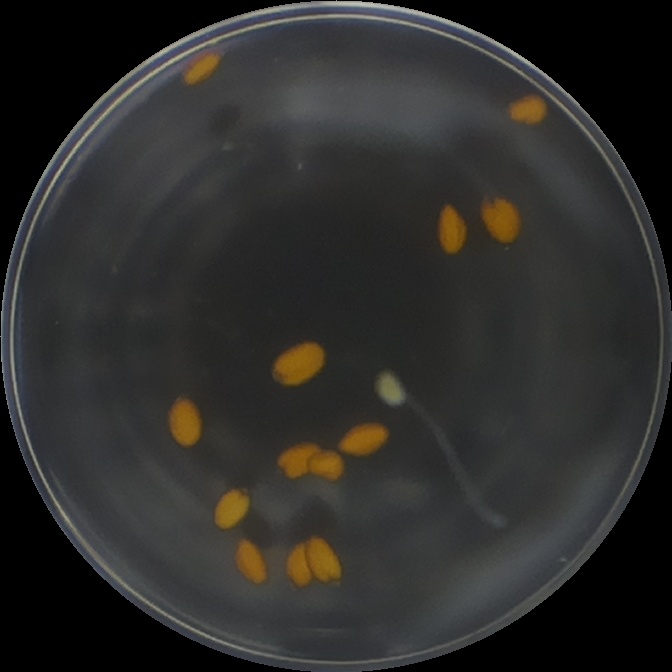

Supplement: Supplementary file 2 — Supplementary Information 2. [file 41598_2020_79115_MOESM2_ESM.zip › PictureOneWell/D5/46_583995]

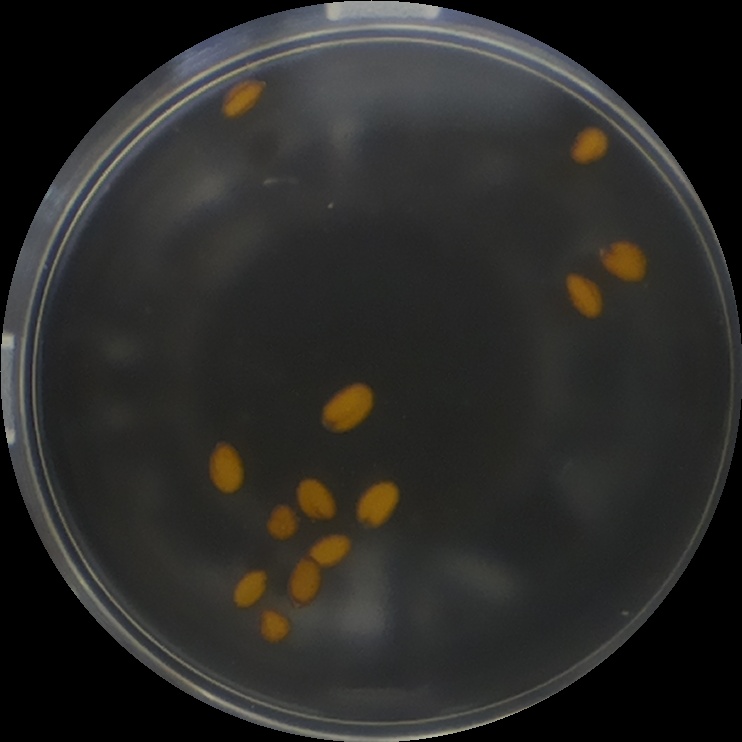

Supplement: Supplementary file 2 — Supplementary Information 2. [file 41598_2020_79115_MOESM2_ESM.zip › PictureOneWell/D5/3_575940]

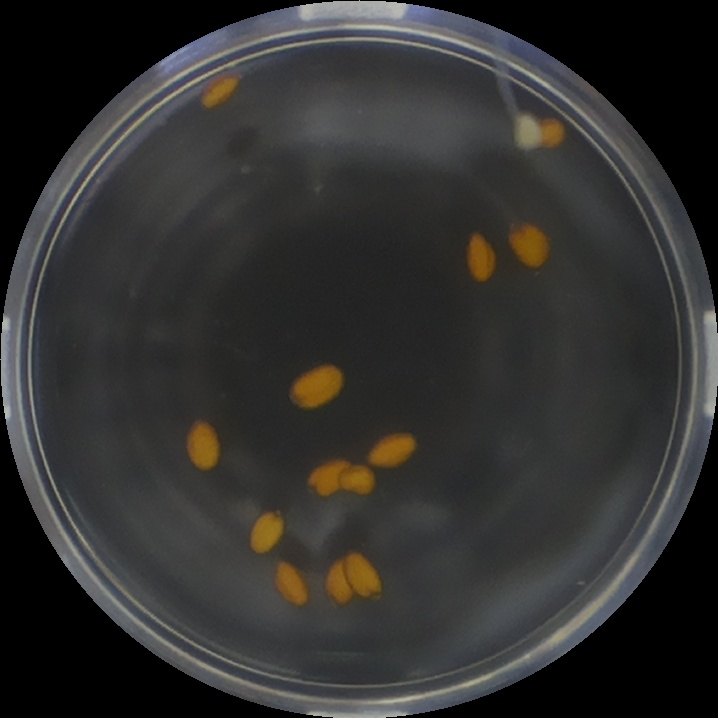

Supplement: Supplementary file 2 — Supplementary Information 2. [file 41598_2020_79115_MOESM2_ESM.zip › PictureOneWell/D5/34_581678]

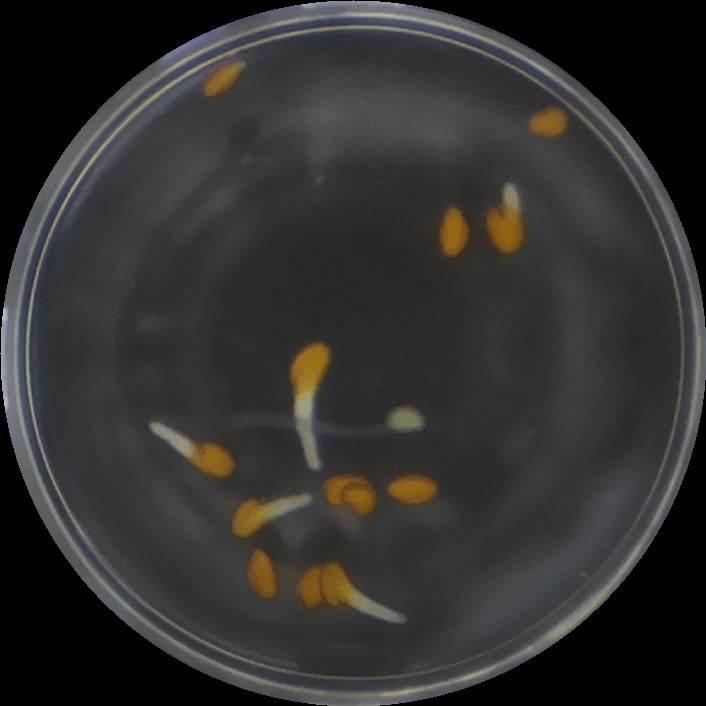

Supplement: Supplementary file 2 — Supplementary Information 2. [file 41598_2020_79115_MOESM2_ESM.zip › PictureOneWell/D5/123_598765]

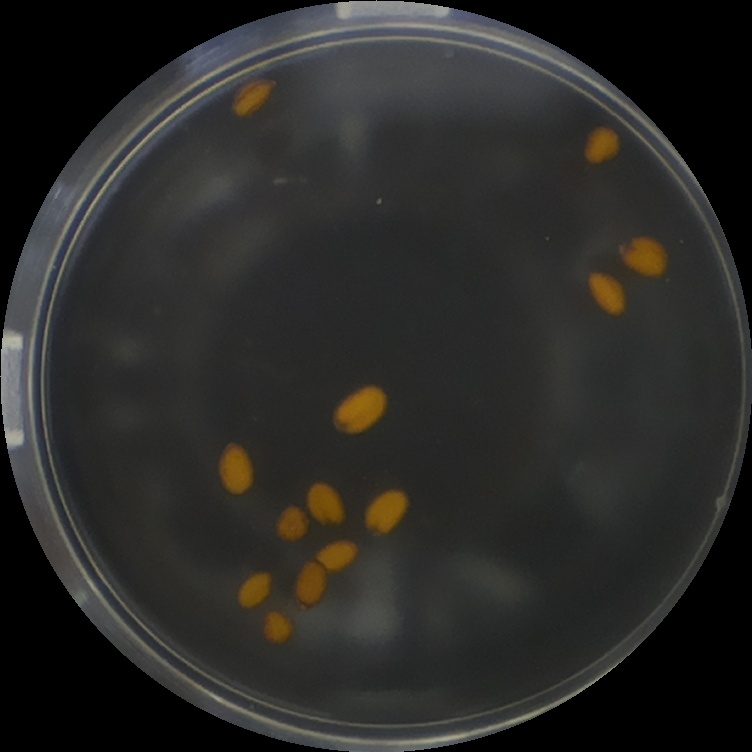

Supplement: Supplementary file 2 — Supplementary Information 2. [file 41598_2020_79115_MOESM2_ESM.zip › PictureOneWell/D5/1_575544]

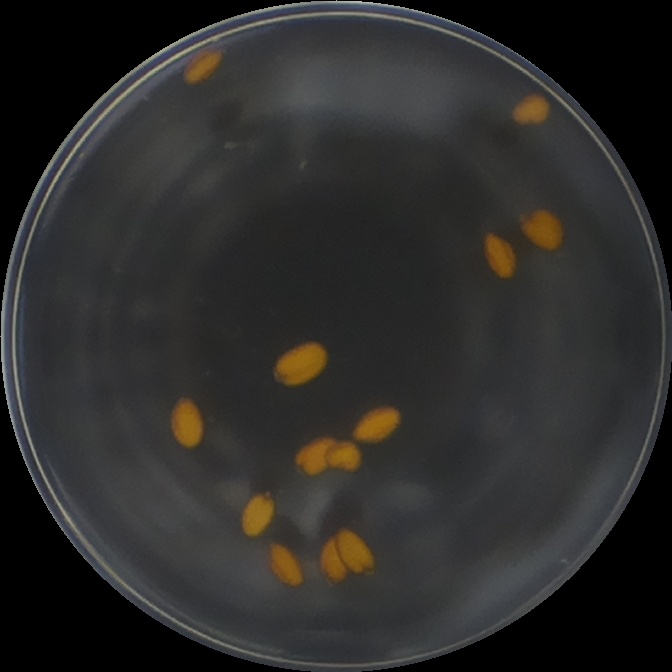

Supplement: Supplementary file 2 — Supplementary Information 2. [file 41598_2020_79115_MOESM2_ESM.zip › PictureOneWell/D5/17_578376]

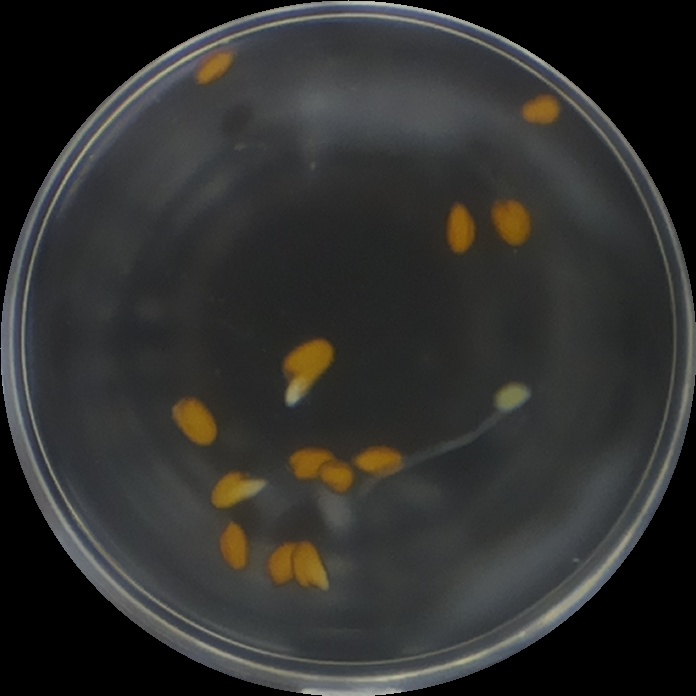

Supplement: Supplementary file 2 — Supplementary Information 2. [file 41598_2020_79115_MOESM2_ESM.zip › PictureOneWell/D5/92_592812]

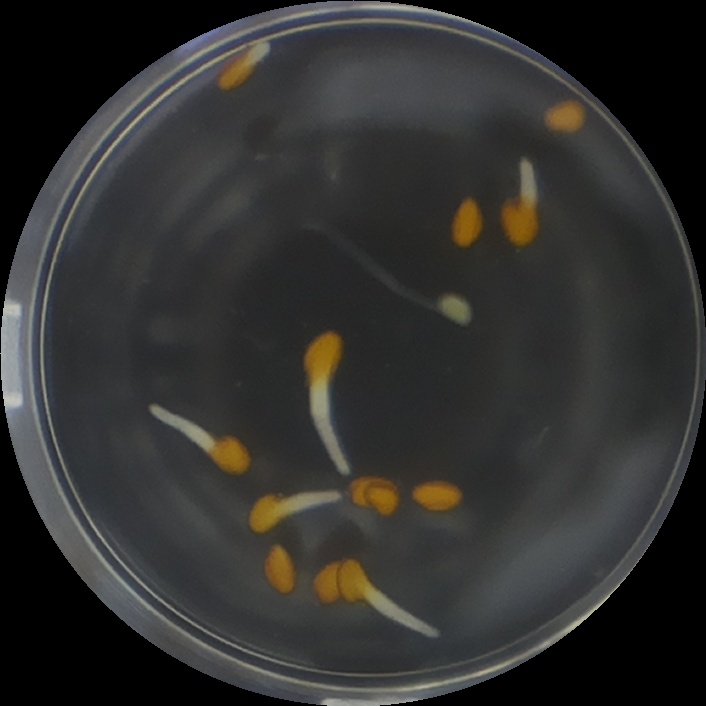

Supplement: Supplementary file 2 — Supplementary Information 2. [file 41598_2020_79115_MOESM2_ESM.zip › PictureOneWell/D5/133_600736]

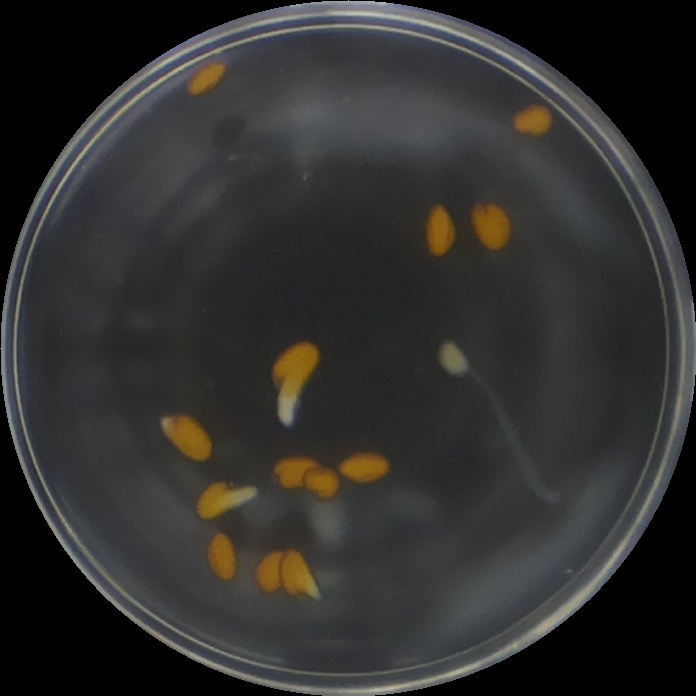

Supplement: Supplementary file 2 — Supplementary Information 2. [file 41598_2020_79115_MOESM2_ESM.zip › PictureOneWell/D5/101_594586]

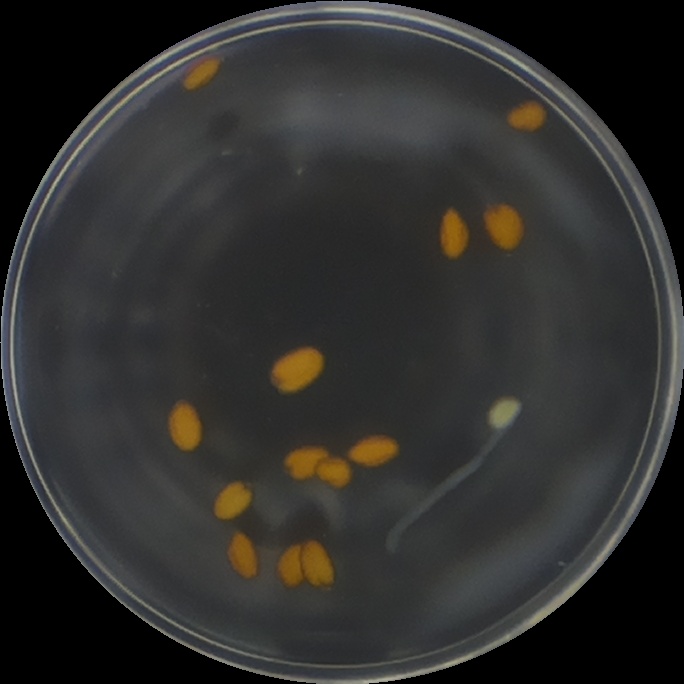

Supplement: Supplementary file 2 — Supplementary Information 2. [file 41598_2020_79115_MOESM2_ESM.zip › PictureOneWell/D5/63_587279]

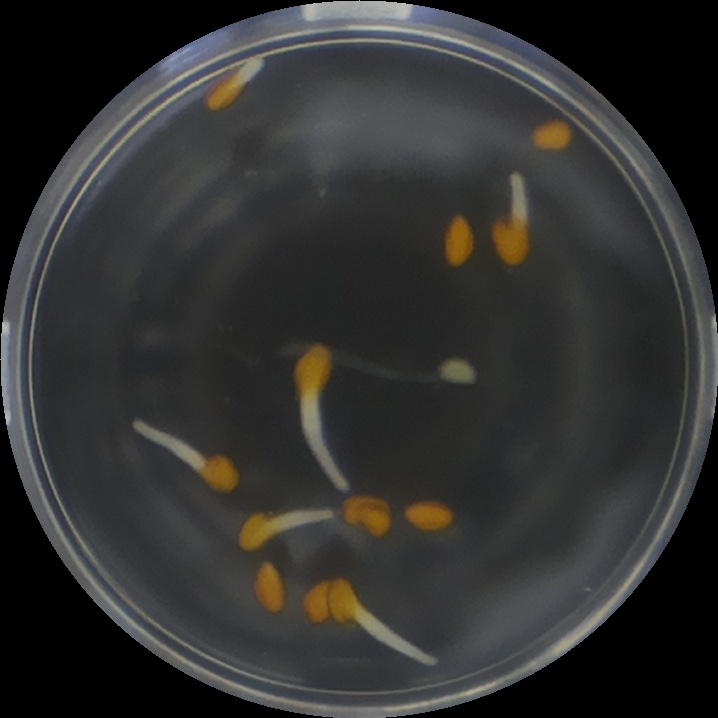

Supplement: Supplementary file 2 — Supplementary Information 2. [file 41598_2020_79115_MOESM2_ESM.zip › PictureOneWell/D5/137_601440]

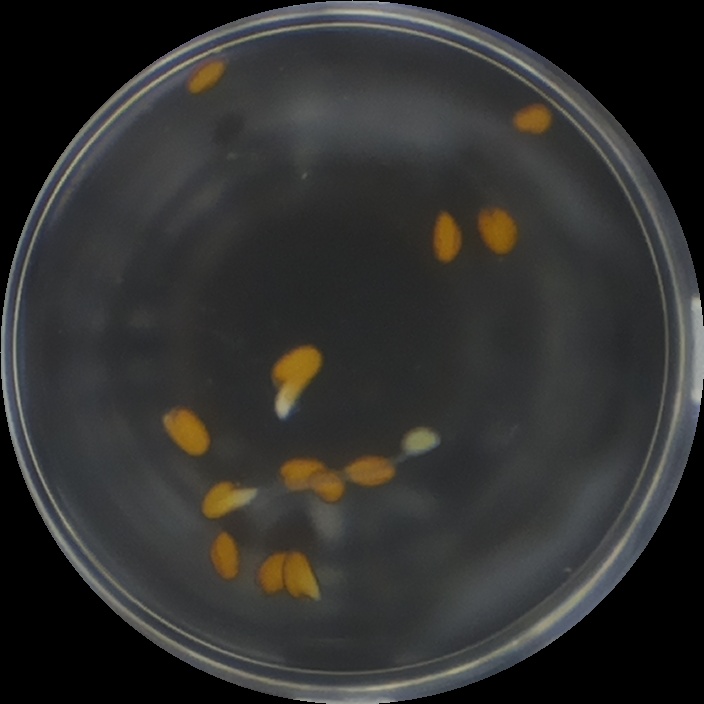

Supplement: Supplementary file 2 — Supplementary Information 2. [file 41598_2020_79115_MOESM2_ESM.zip › PictureOneWell/D5/95_593373]

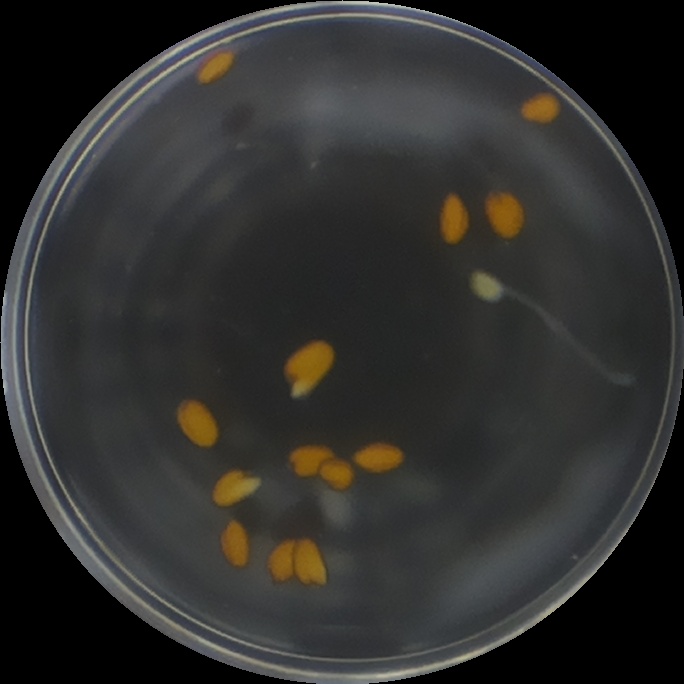

Supplement: Supplementary file 2 — Supplementary Information 2. [file 41598_2020_79115_MOESM2_ESM.zip › PictureOneWell/D5/81_590670]

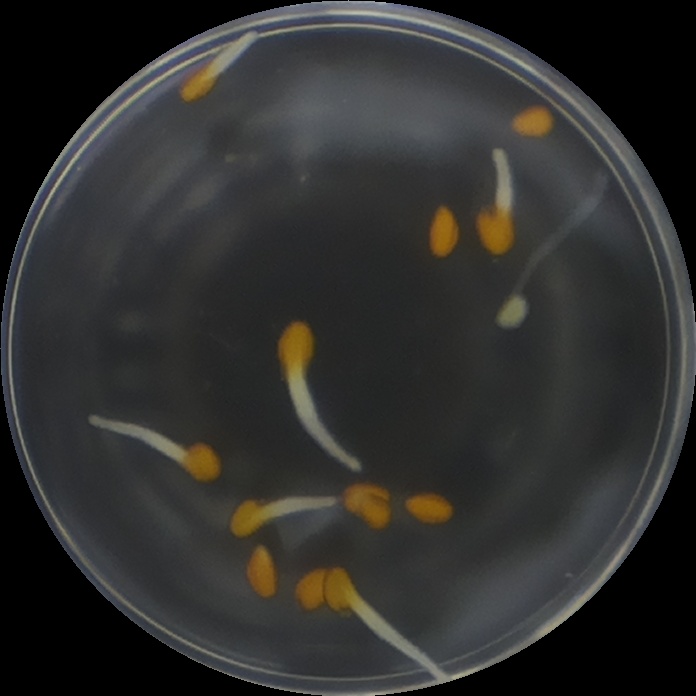

Supplement: Supplementary file 2 — Supplementary Information 2. [file 41598_2020_79115_MOESM2_ESM.zip › PictureOneWell/D5/145_603131]

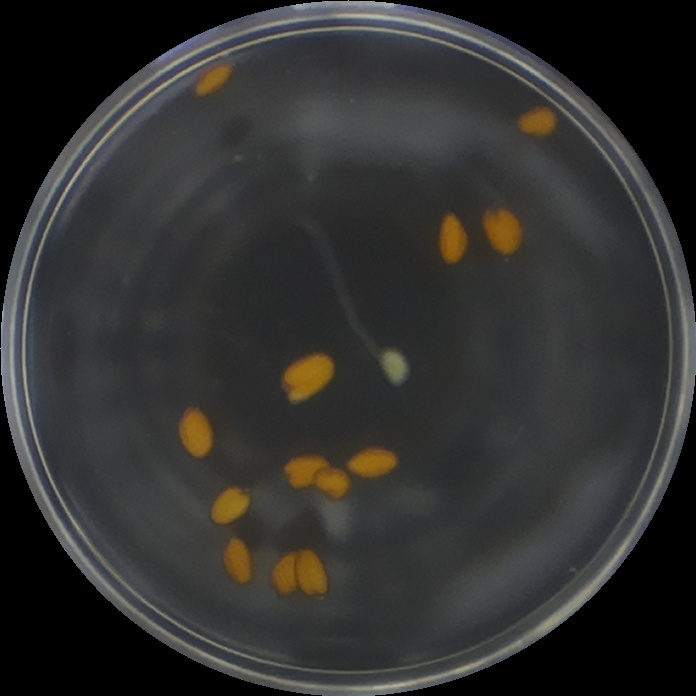

Supplement: Supplementary file 2 — Supplementary Information 2. [file 41598_2020_79115_MOESM2_ESM.zip › PictureOneWell/D5/69_588378]

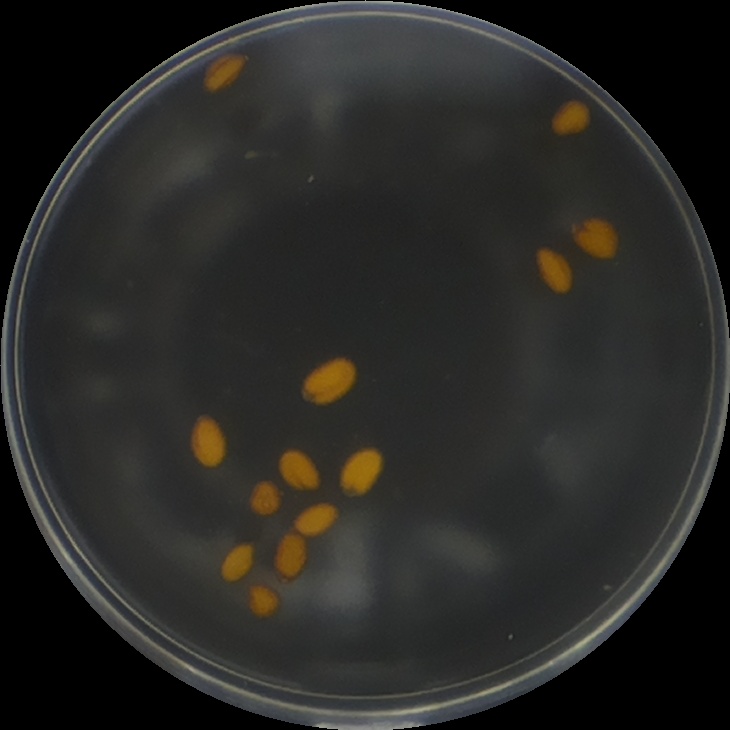

Supplement: Supplementary file 2 — Supplementary Information 2. [file 41598_2020_79115_MOESM2_ESM.zip › PictureOneWell/D5/5_576291]

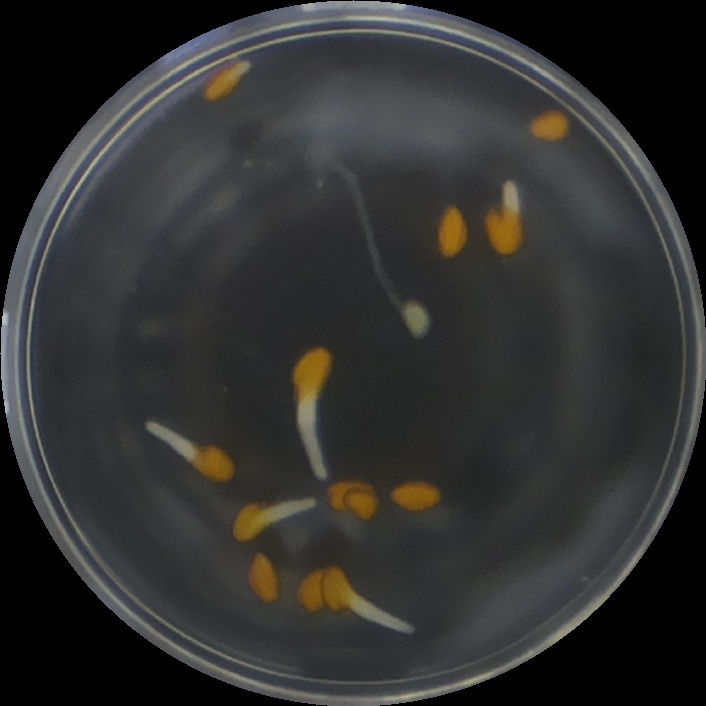

Supplement: Supplementary file 2 — Supplementary Information 2. [file 41598_2020_79115_MOESM2_ESM.zip › PictureOneWell/D5/126_599310]

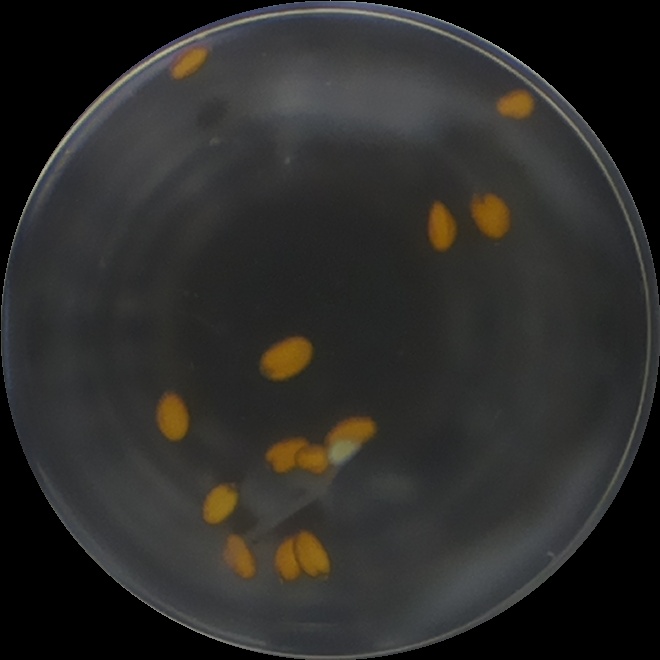

Supplement: Supplementary file 2 — Supplementary Information 2. [file 41598_2020_79115_MOESM2_ESM.zip › PictureOneWell/D5/45_583781]

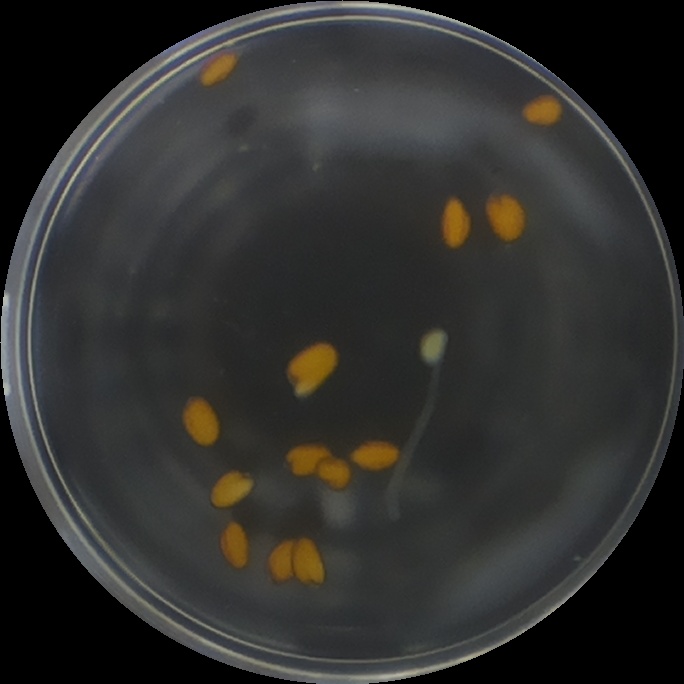

Supplement: Supplementary file 2 — Supplementary Information 2. [file 41598_2020_79115_MOESM2_ESM.zip › PictureOneWell/D5/76_589736]

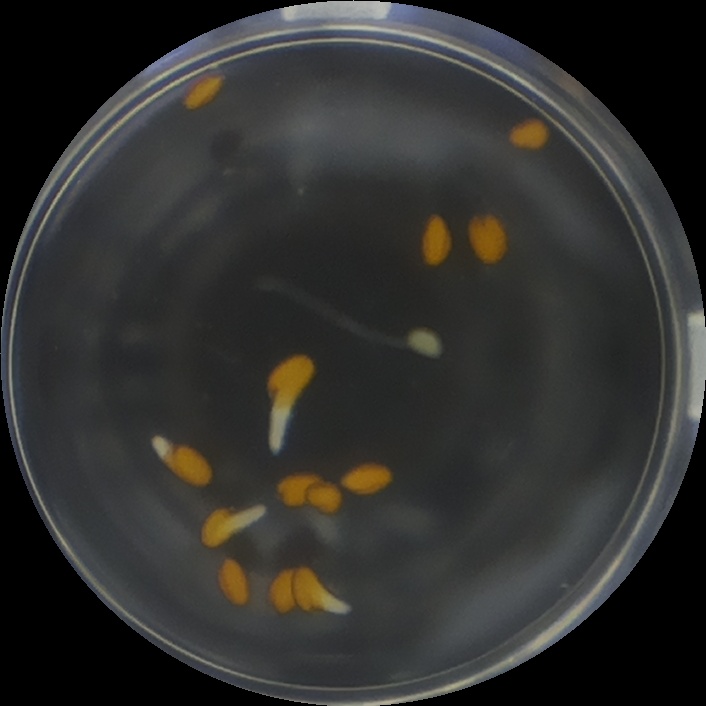

Supplement: Supplementary file 2 — Supplementary Information 2. [file 41598_2020_79115_MOESM2_ESM.zip › PictureOneWell/D5/109_596056]

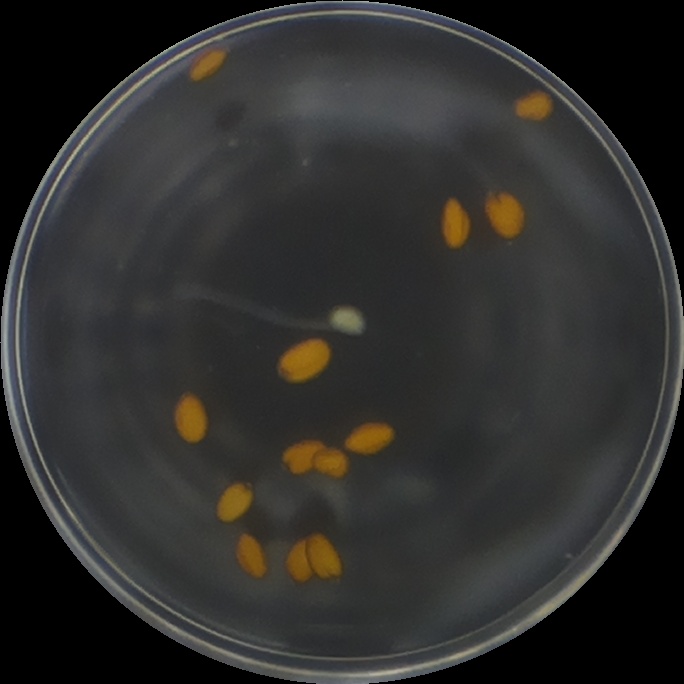

Supplement: Supplementary file 2 — Supplementary Information 2. [file 41598_2020_79115_MOESM2_ESM.zip › PictureOneWell/D5/47_584201]

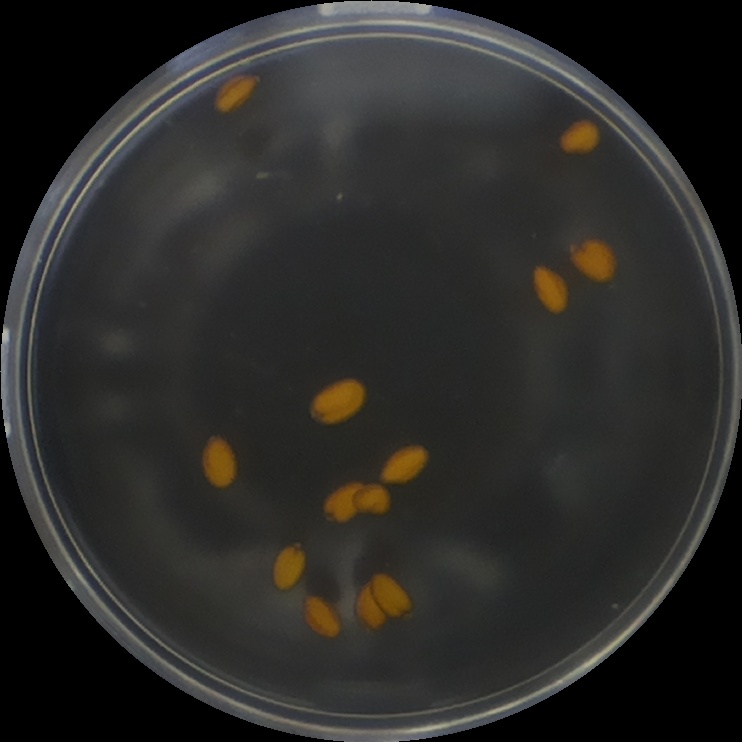

Supplement: Supplementary file 2 — Supplementary Information 2. [file 41598_2020_79115_MOESM2_ESM.zip › PictureOneWell/D5/8_576903]

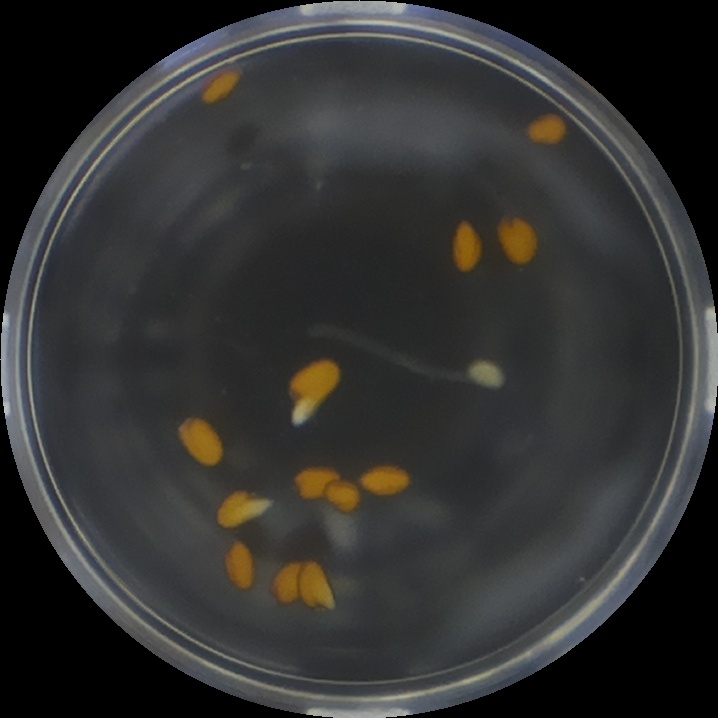

Supplement: Supplementary file 2 — Supplementary Information 2. [file 41598_2020_79115_MOESM2_ESM.zip › PictureOneWell/D5/91_592621]

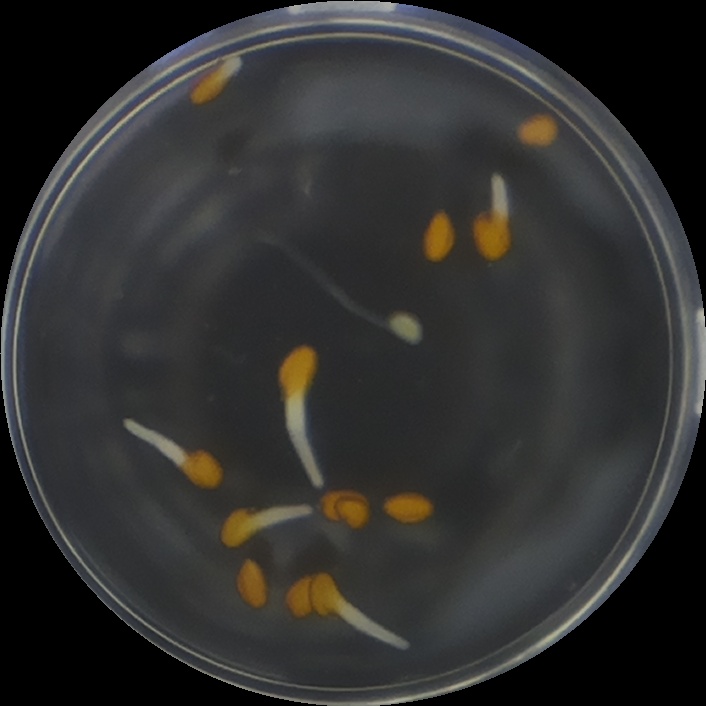

Supplement: Supplementary file 2 — Supplementary Information 2. [file 41598_2020_79115_MOESM2_ESM.zip › PictureOneWell/D5/132_600523]

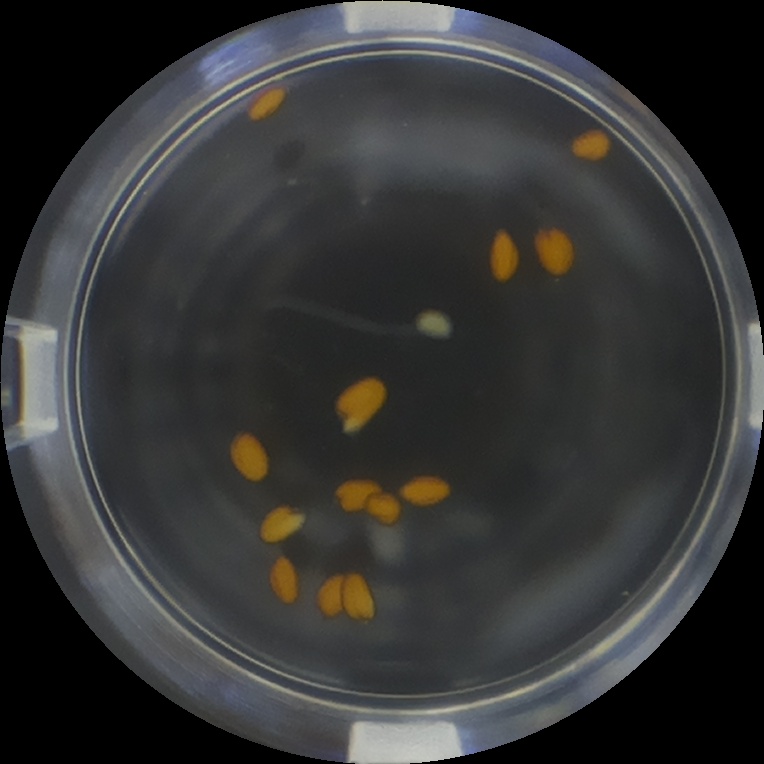

Supplement: Supplementary file 2 — Supplementary Information 2. [file 41598_2020_79115_MOESM2_ESM.zip › PictureOneWell/D5/78_590163]

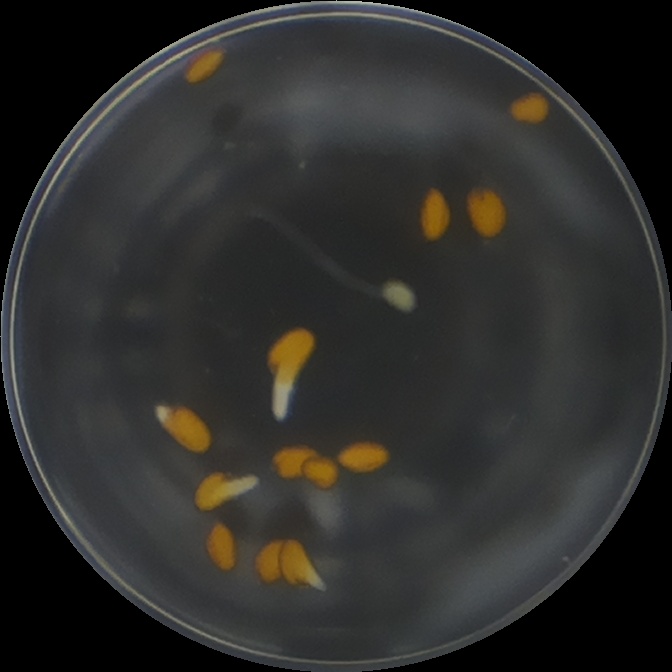

Supplement: Supplementary file 2 — Supplementary Information 2. [file 41598_2020_79115_MOESM2_ESM.zip › PictureOneWell/D5/105_595326]

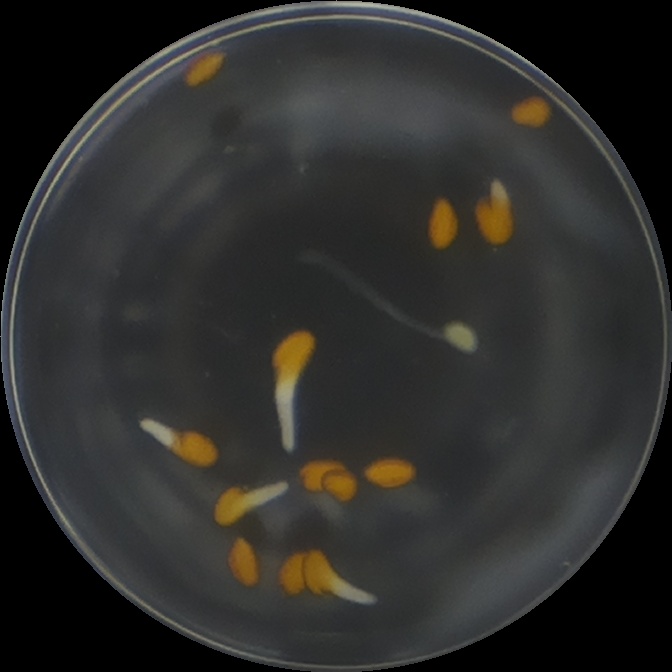

Supplement: Supplementary file 2 — Supplementary Information 2. [file 41598_2020_79115_MOESM2_ESM.zip › PictureOneWell/D5/118_597779]

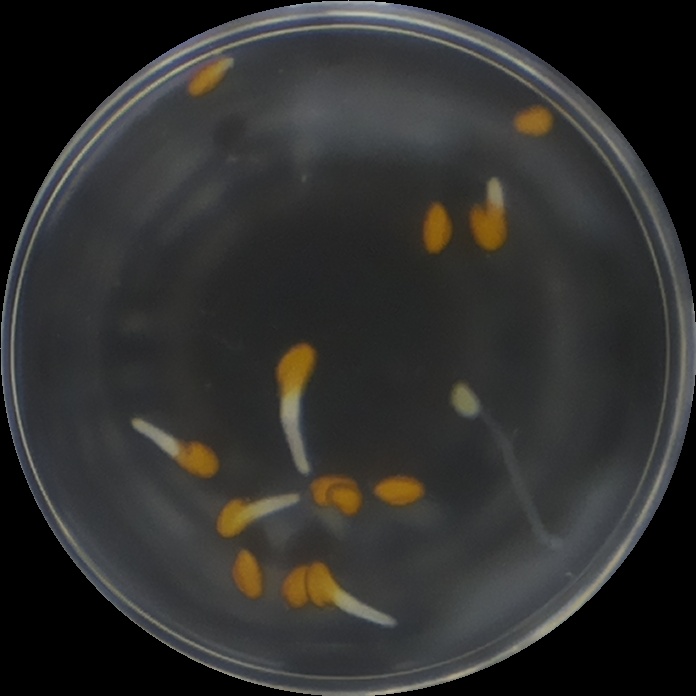

Supplement: Supplementary file 2 — Supplementary Information 2. [file 41598_2020_79115_MOESM2_ESM.zip › PictureOneWell/D5/125_599172]

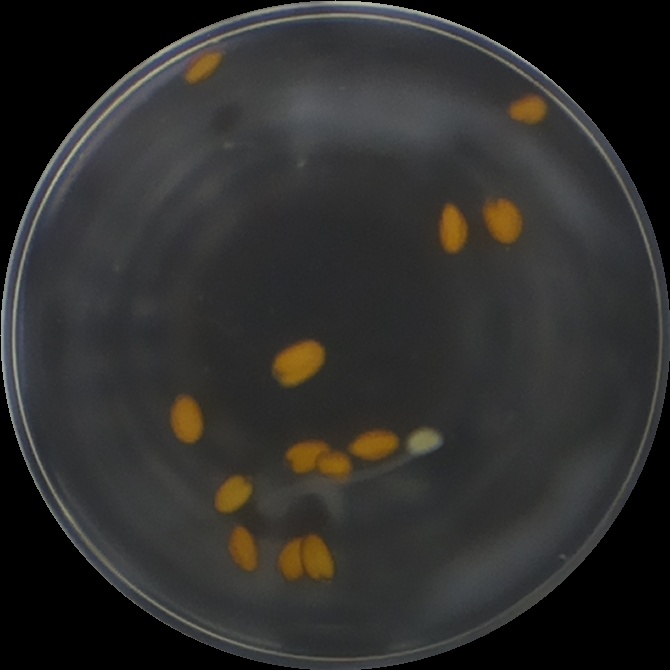

Supplement: Supplementary file 2 — Supplementary Information 2. [file 41598_2020_79115_MOESM2_ESM.zip › PictureOneWell/D5/65_587657]

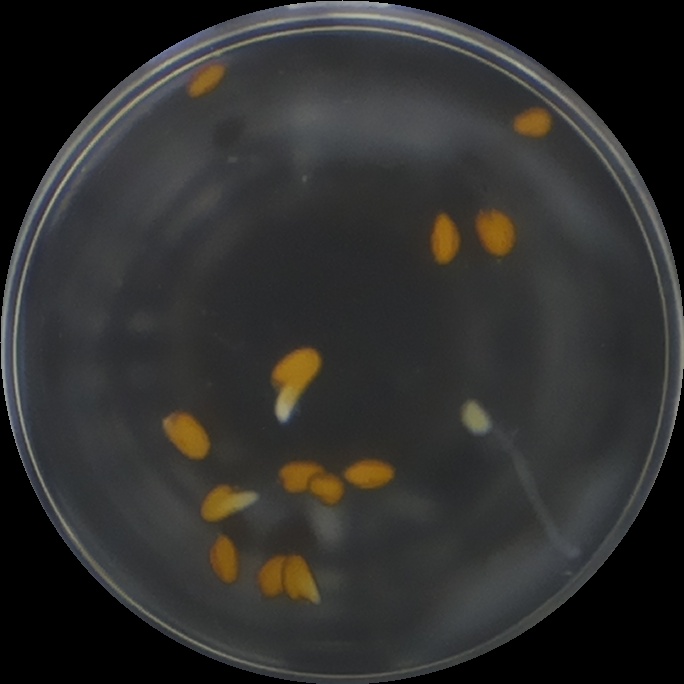

Supplement: Supplementary file 2 — Supplementary Information 2. [file 41598_2020_79115_MOESM2_ESM.zip › PictureOneWell/D5/96_593623]

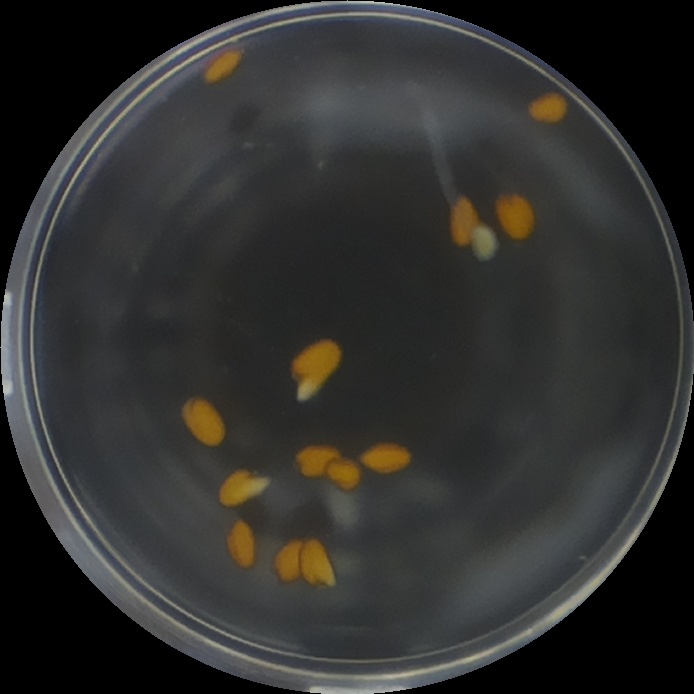

Supplement: Supplementary file 2 — Supplementary Information 2. [file 41598_2020_79115_MOESM2_ESM.zip › PictureOneWell/D5/86_591708]

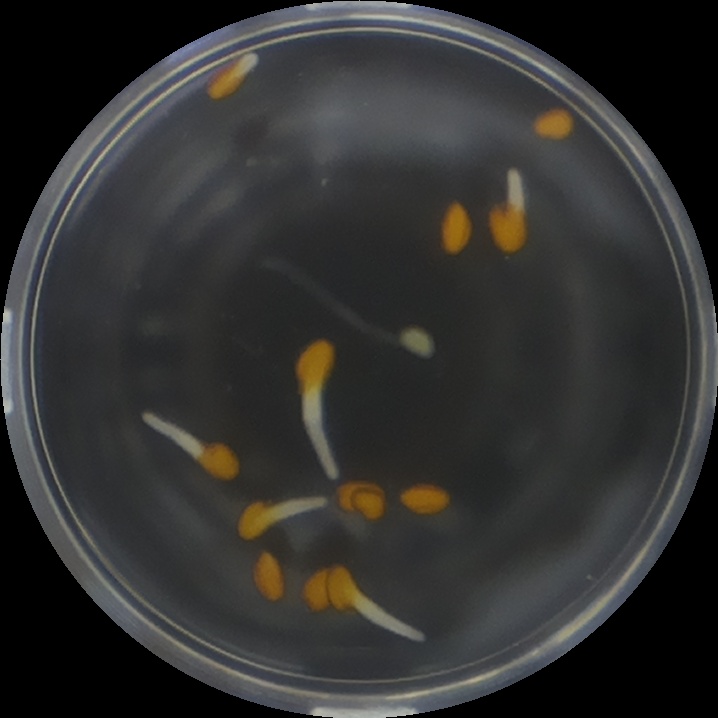

Supplement: Supplementary file 2 — Supplementary Information 2. [file 41598_2020_79115_MOESM2_ESM.zip › PictureOneWell/D5/131_600292]

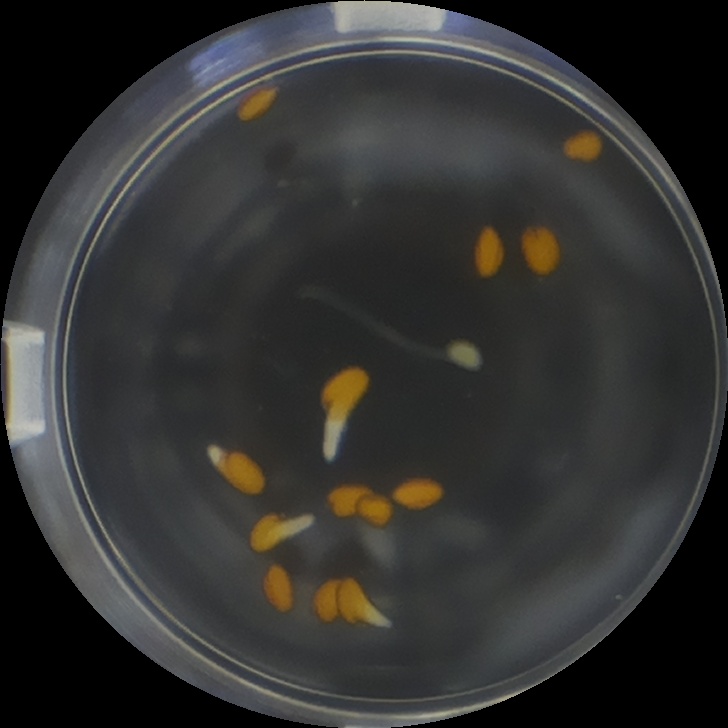

Supplement: Supplementary file 2 — Supplementary Information 2. [file 41598_2020_79115_MOESM2_ESM.zip › PictureOneWell/D5/107_595694]

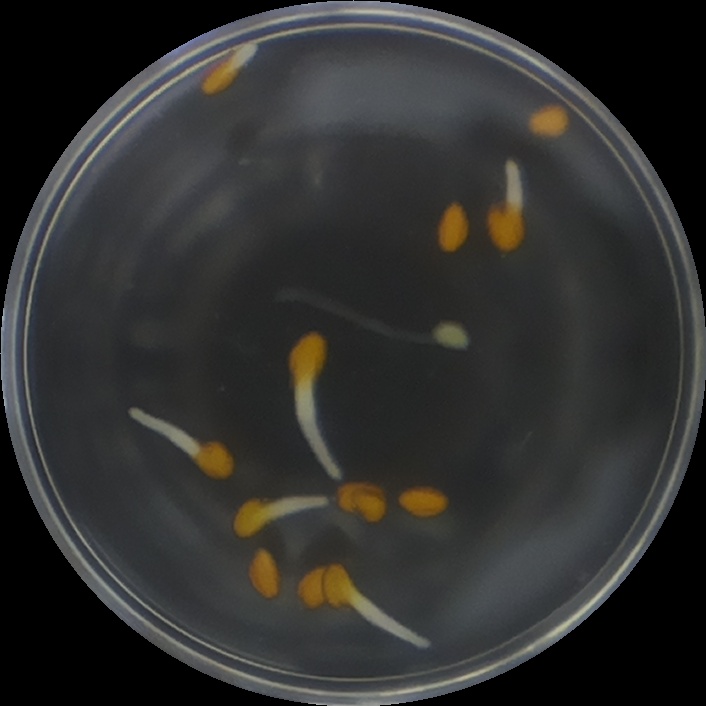

Supplement: Supplementary file 2 — Supplementary Information 2. [file 41598_2020_79115_MOESM2_ESM.zip › PictureOneWell/D5/136_601229]

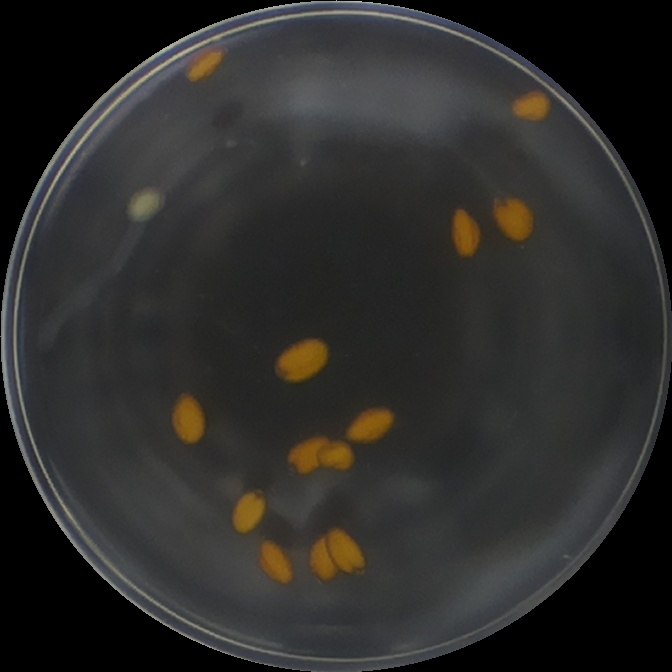

Supplement: Supplementary file 2 — Supplementary Information 2. [file 41598_2020_79115_MOESM2_ESM.zip › PictureOneWell/D5/29_580730]

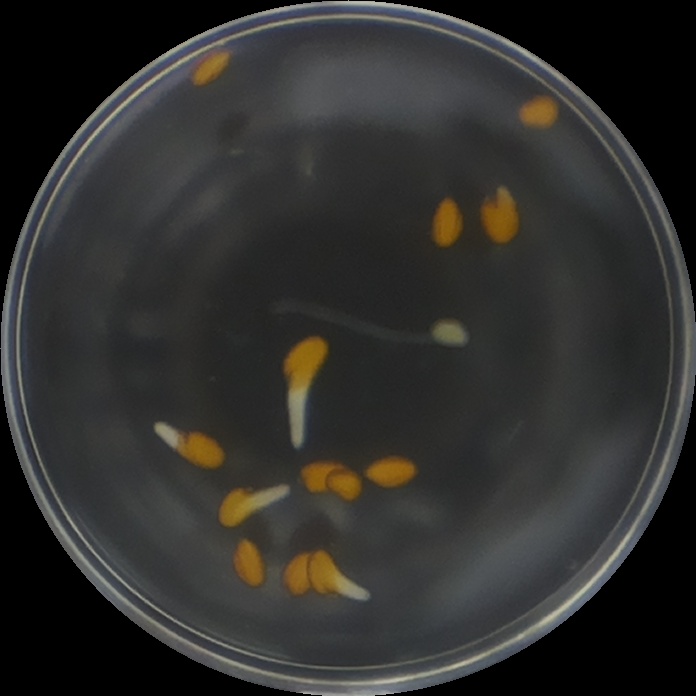

Supplement: Supplementary file 2 — Supplementary Information 2. [file 41598_2020_79115_MOESM2_ESM.zip › PictureOneWell/D5/114_597083]

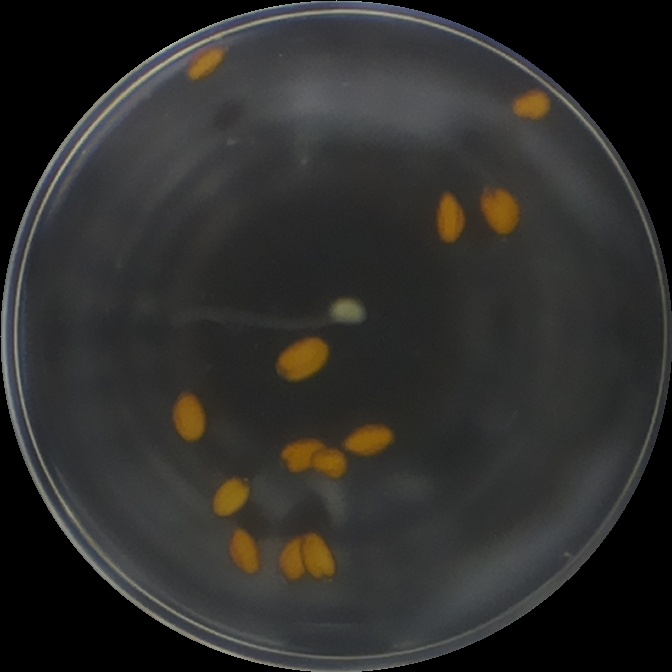

Supplement: Supplementary file 2 — Supplementary Information 2. [file 41598_2020_79115_MOESM2_ESM.zip › PictureOneWell/D5/51_584993]

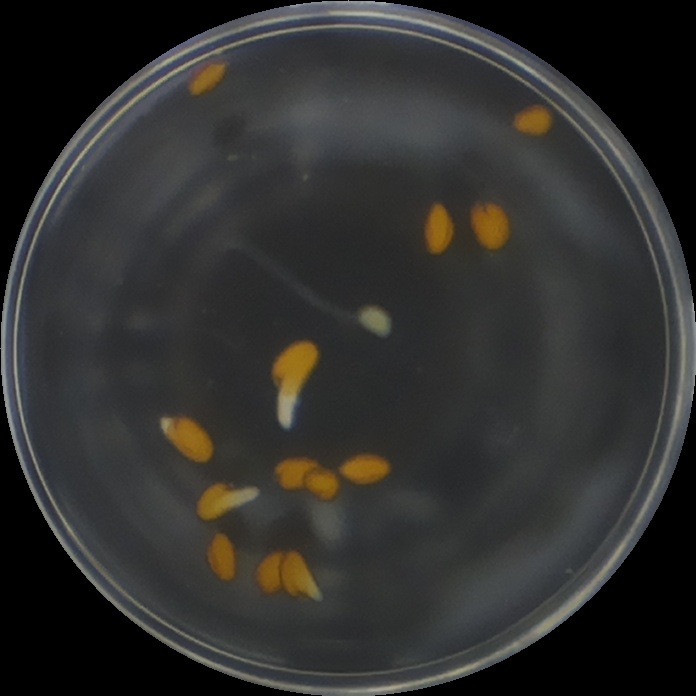

Supplement: Supplementary file 2 — Supplementary Information 2. [file 41598_2020_79115_MOESM2_ESM.zip › PictureOneWell/D5/103_594922]

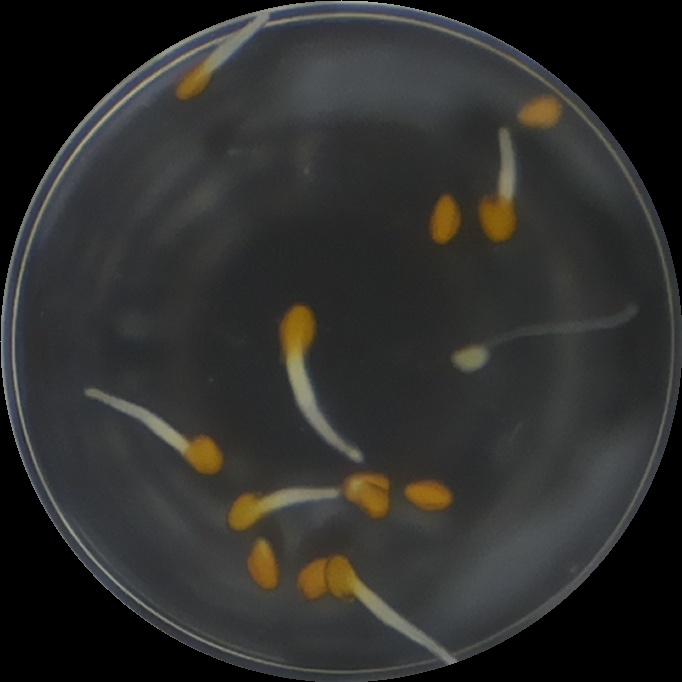

Supplement: Supplementary file 2 — Supplementary Information 2. [file 41598_2020_79115_MOESM2_ESM.zip › PictureOneWell/D5/149_603823]

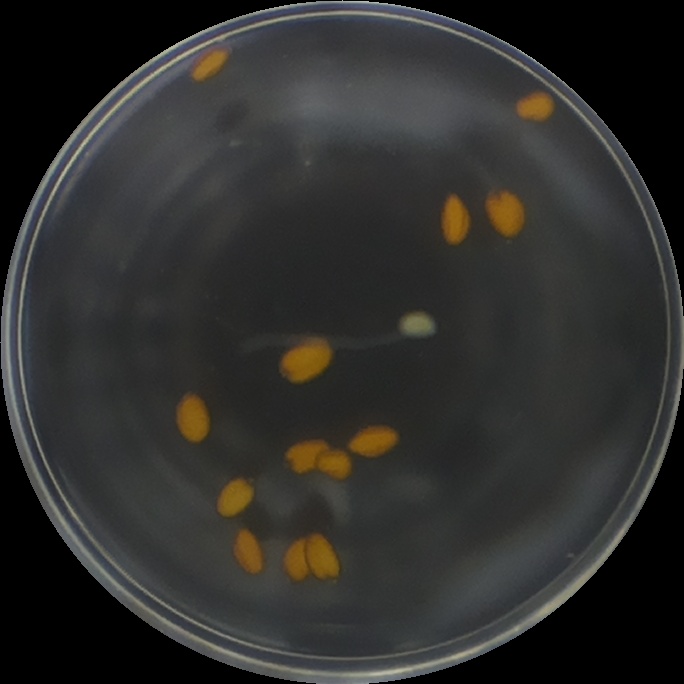

Supplement: Supplementary file 2 — Supplementary Information 2. [file 41598_2020_79115_MOESM2_ESM.zip › PictureOneWell/D5/55_585758]

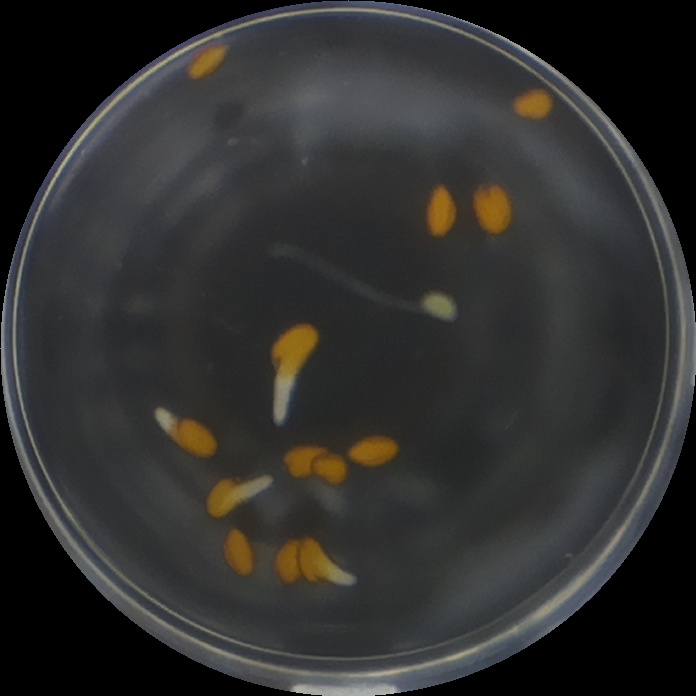

Supplement: Supplementary file 2 — Supplementary Information 2. [file 41598_2020_79115_MOESM2_ESM.zip › PictureOneWell/D5/110_596231]

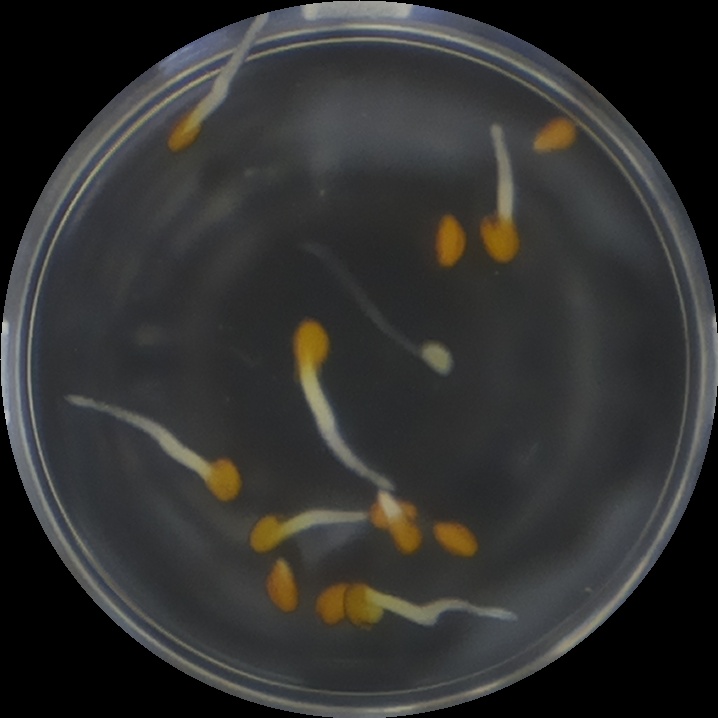

Supplement: Supplementary file 2 — Supplementary Information 2. [file 41598_2020_79115_MOESM2_ESM.zip › PictureOneWell/D5/161_606367]

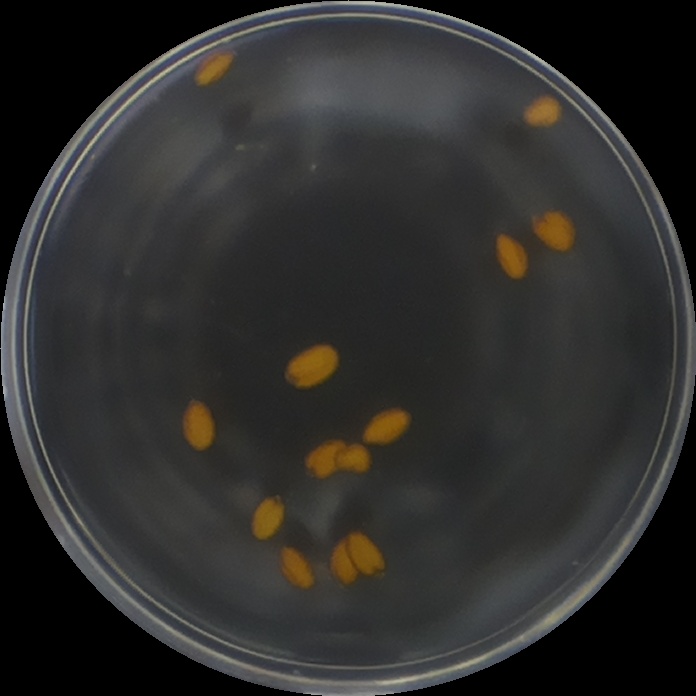

Supplement: Supplementary file 2 — Supplementary Information 2. [file 41598_2020_79115_MOESM2_ESM.zip › PictureOneWell/D5/16_578344]

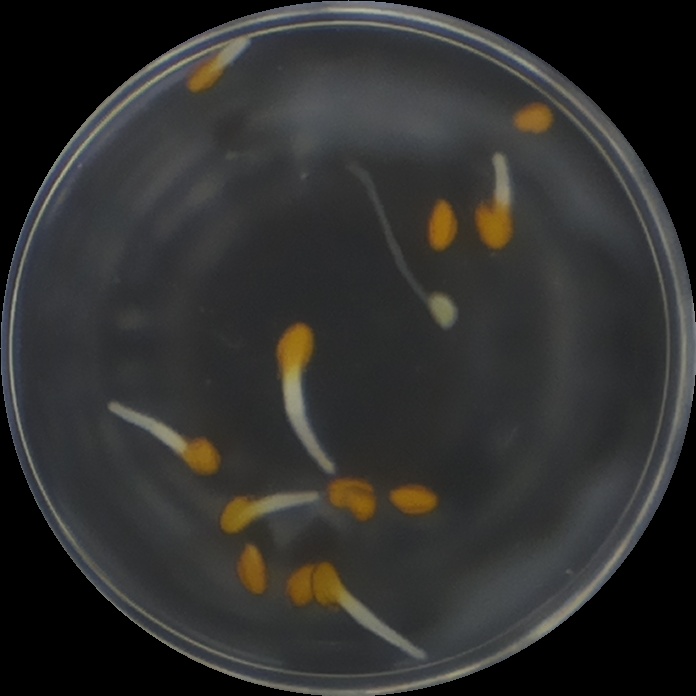

Supplement: Supplementary file 2 — Supplementary Information 2. [file 41598_2020_79115_MOESM2_ESM.zip › PictureOneWell/D5/140_601995]

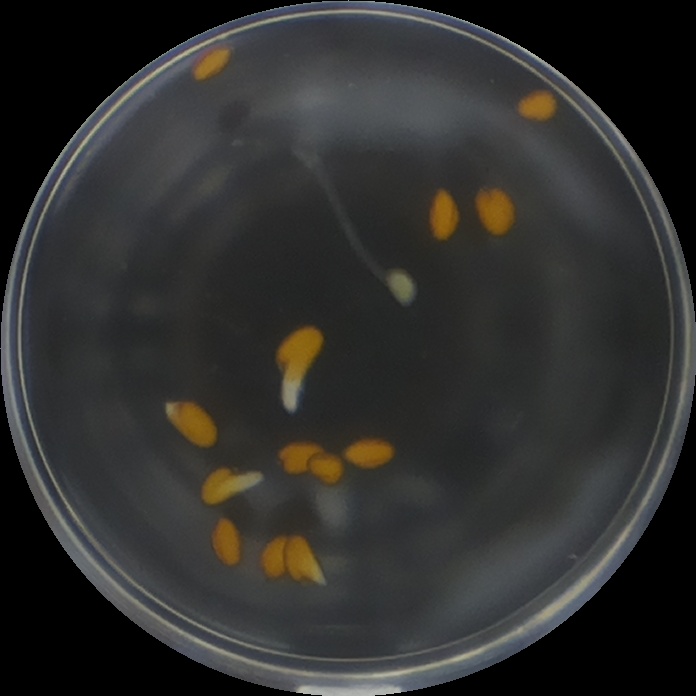

Supplement: Supplementary file 2 — Supplementary Information 2. [file 41598_2020_79115_MOESM2_ESM.zip › PictureOneWell/D5/102_594729]

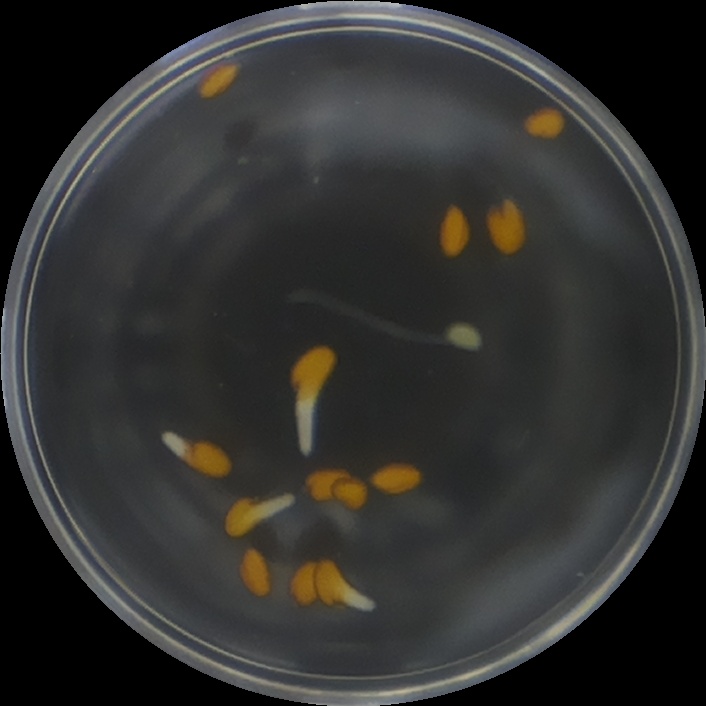

Supplement: Supplementary file 2 — Supplementary Information 2. [file 41598_2020_79115_MOESM2_ESM.zip › PictureOneWell/D5/113_596894]

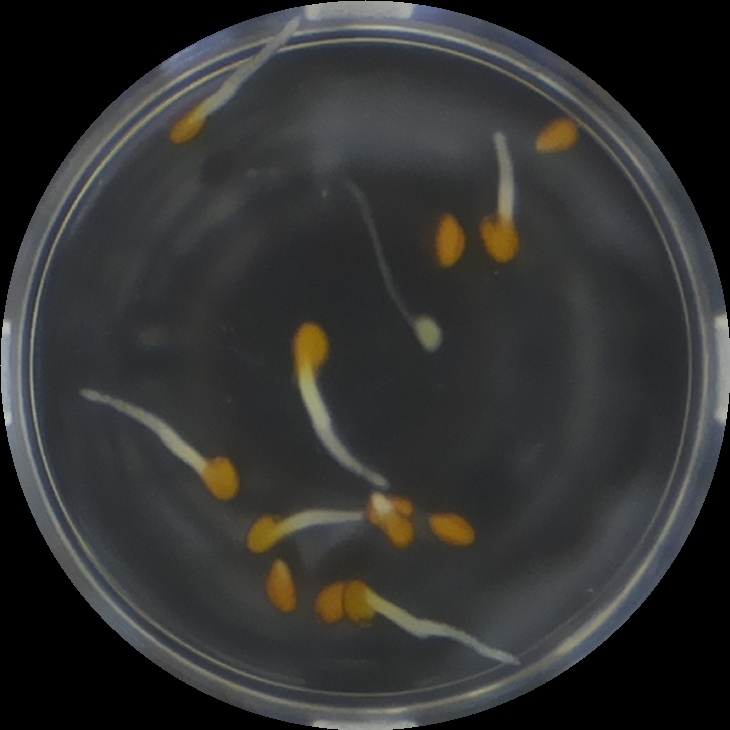

Supplement: Supplementary file 2 — Supplementary Information 2. [file 41598_2020_79115_MOESM2_ESM.zip › PictureOneWell/D5/157_605418]

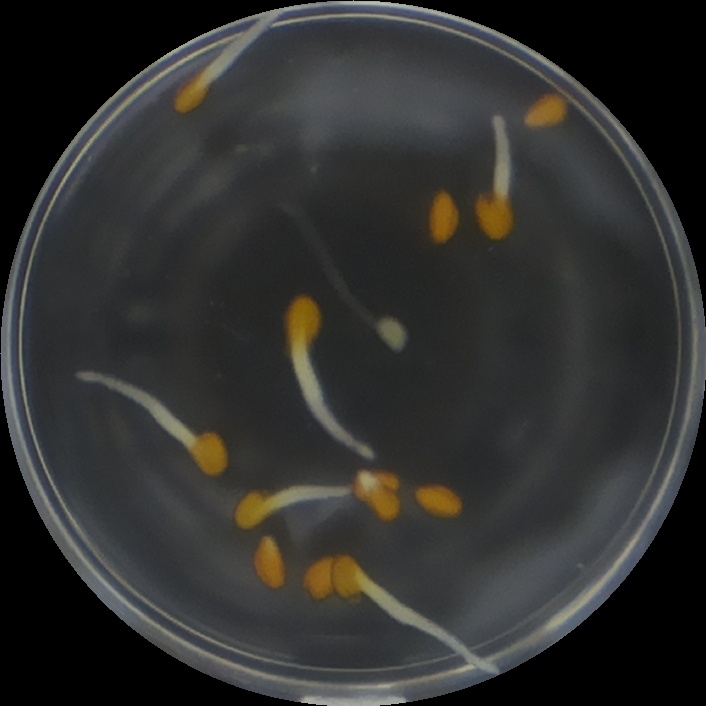

Supplement: Supplementary file 2 — Supplementary Information 2. [file 41598_2020_79115_MOESM2_ESM.zip › PictureOneWell/D5/154_604790]

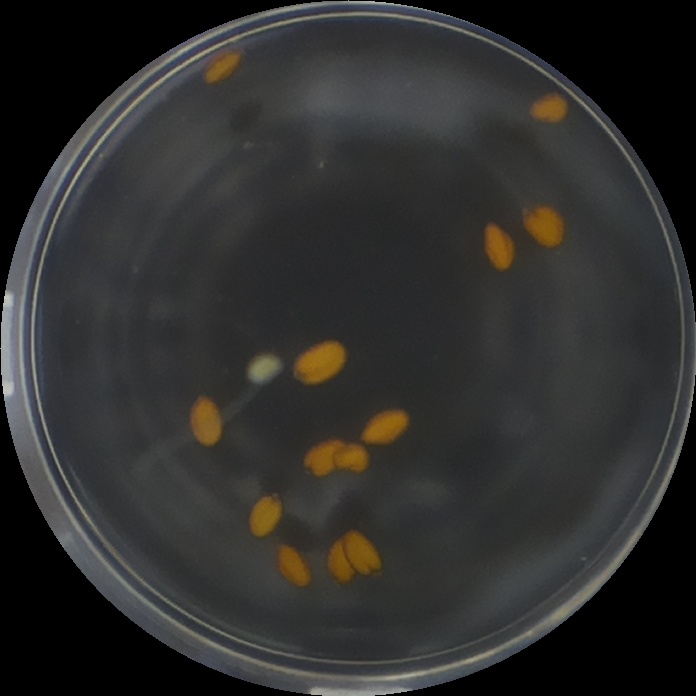

Supplement: Supplementary file 2 — Supplementary Information 2. [file 41598_2020_79115_MOESM2_ESM.zip › PictureOneWell/D5/23_579538]

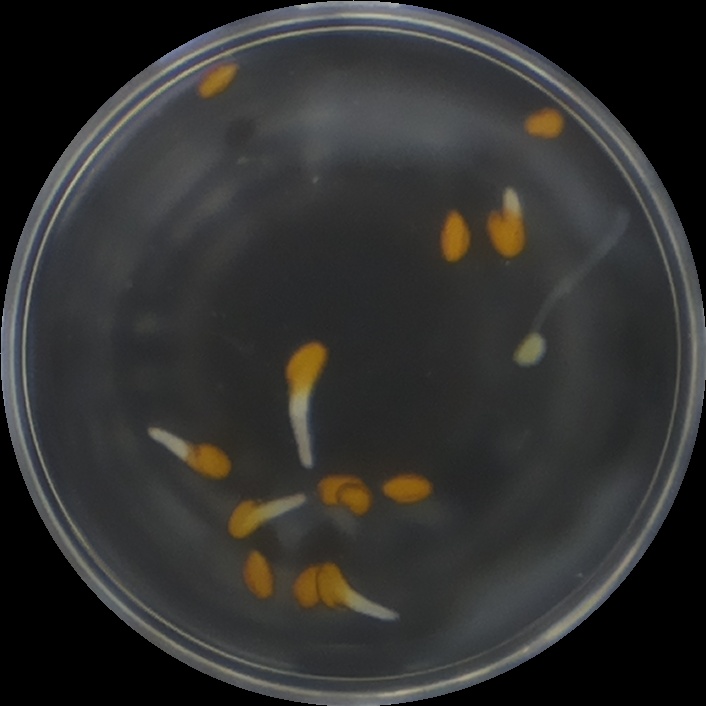

Supplement: Supplementary file 2 — Supplementary Information 2. [file 41598_2020_79115_MOESM2_ESM.zip › PictureOneWell/D5/121_598432]

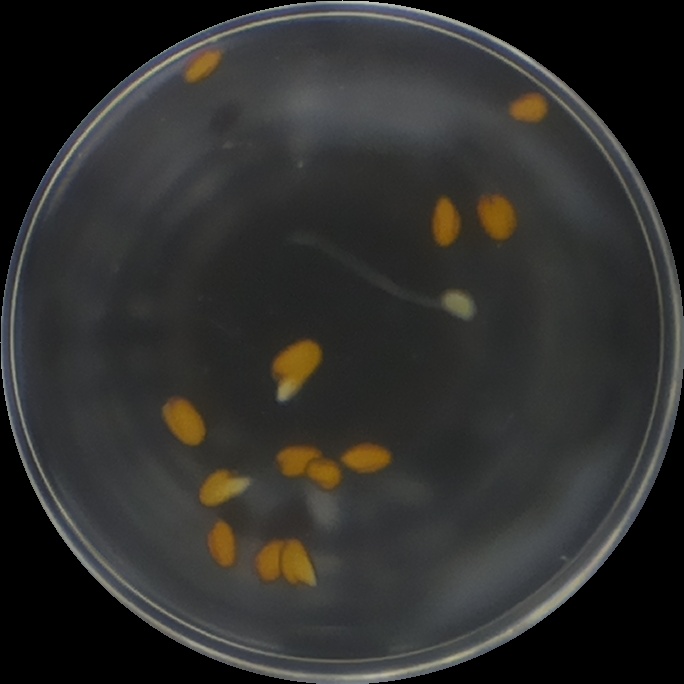

Supplement: Supplementary file 2 — Supplementary Information 2. [file 41598_2020_79115_MOESM2_ESM.zip › PictureOneWell/D5/87_591868]

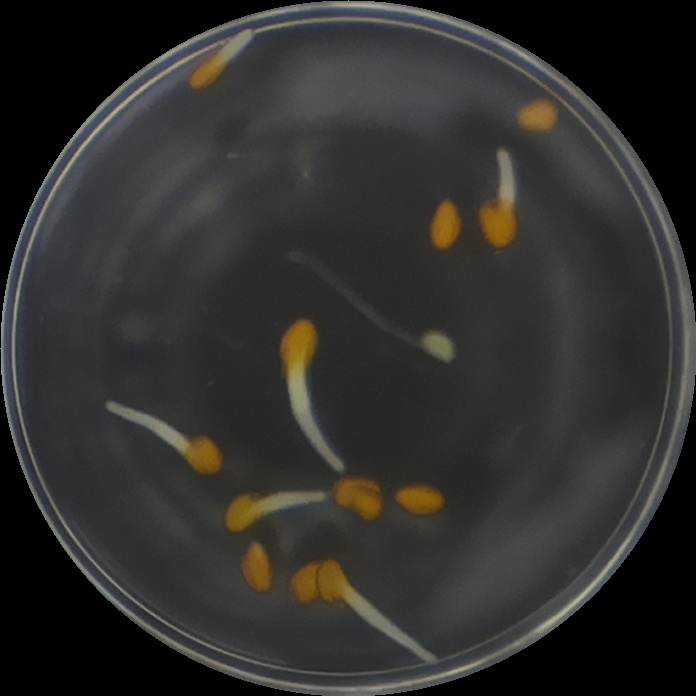

Supplement: Supplementary file 2 — Supplementary Information 2. [file 41598_2020_79115_MOESM2_ESM.zip › PictureOneWell/D5/142_602421]

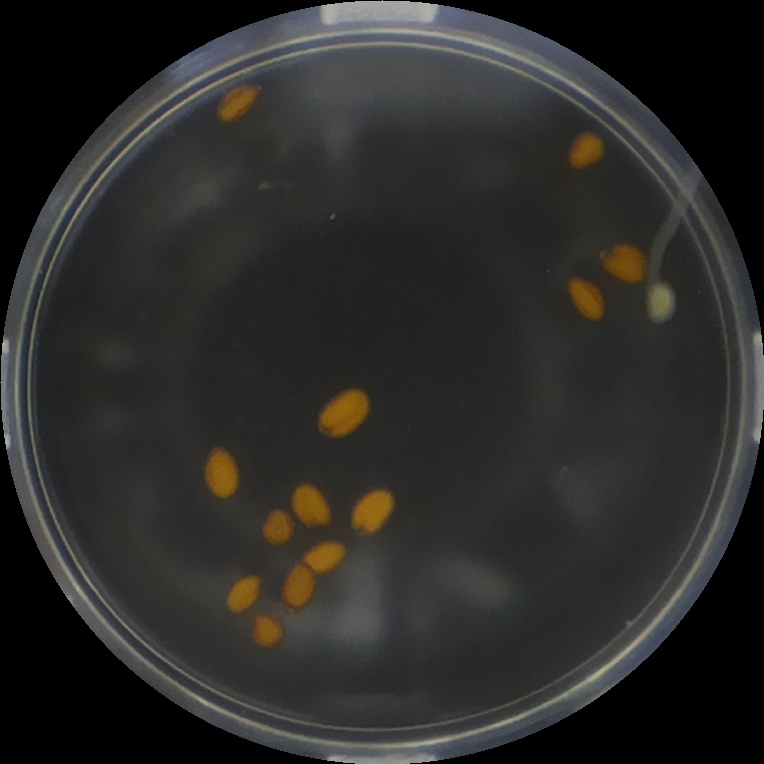

Supplement: Supplementary file 2 — Supplementary Information 2. [file 41598_2020_79115_MOESM2_ESM.zip › PictureOneWell/D5/2_575706]

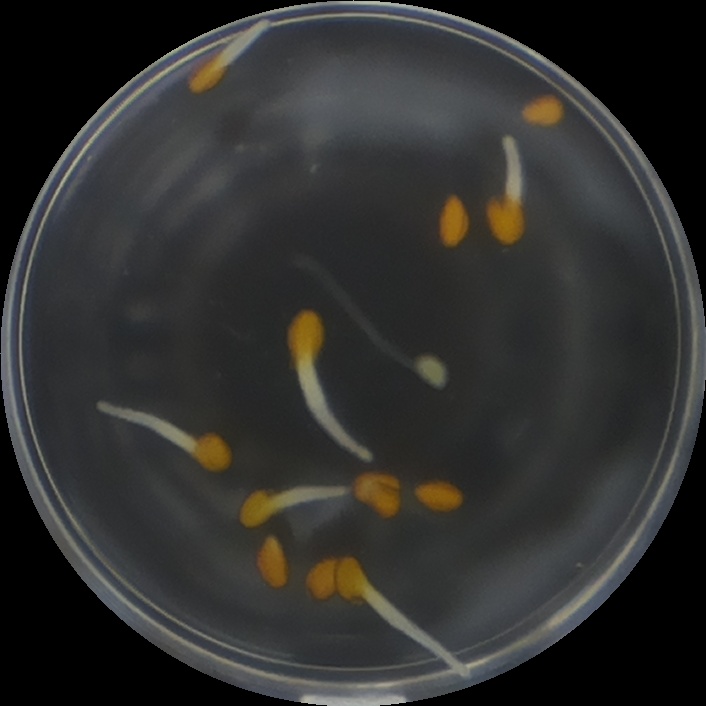

Supplement: Supplementary file 2 — Supplementary Information 2. [file 41598_2020_79115_MOESM2_ESM.zip › PictureOneWell/D5/146_603261]

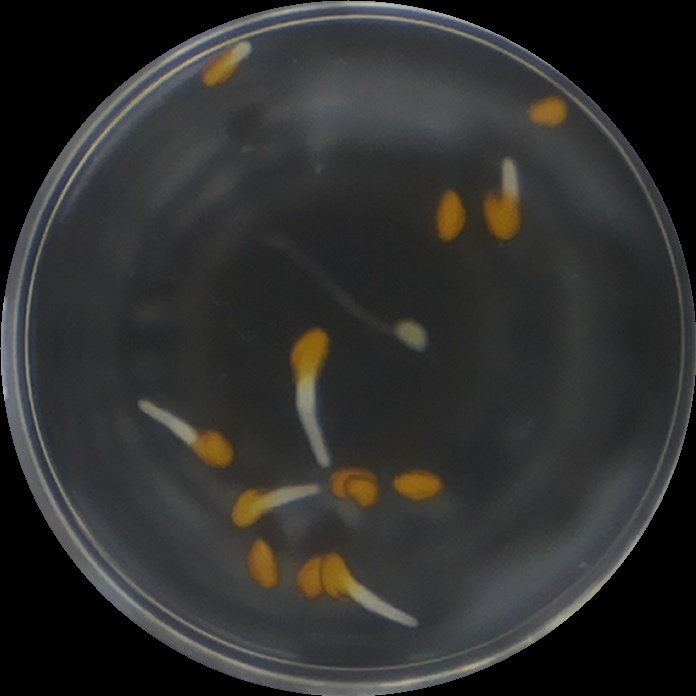

Supplement: Supplementary file 2 — Supplementary Information 2. [file 41598_2020_79115_MOESM2_ESM.zip › PictureOneWell/D5/130_600084]

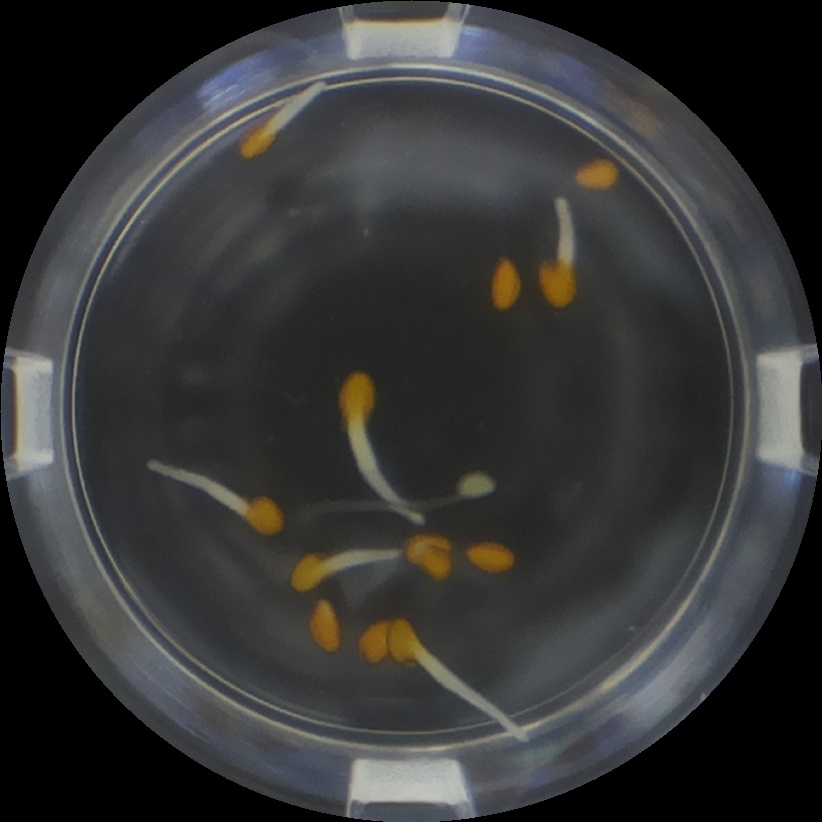

Supplement: Supplementary file 2 — Supplementary Information 2. [file 41598_2020_79115_MOESM2_ESM.zip › PictureOneWell/D5/147_603466]

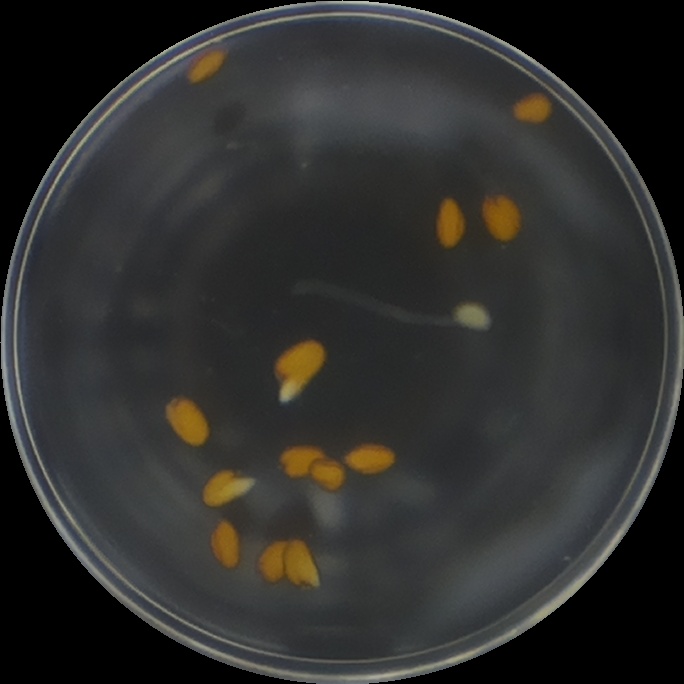

Supplement: Supplementary file 2 — Supplementary Information 2. [file 41598_2020_79115_MOESM2_ESM.zip › PictureOneWell/D5/88_592094]

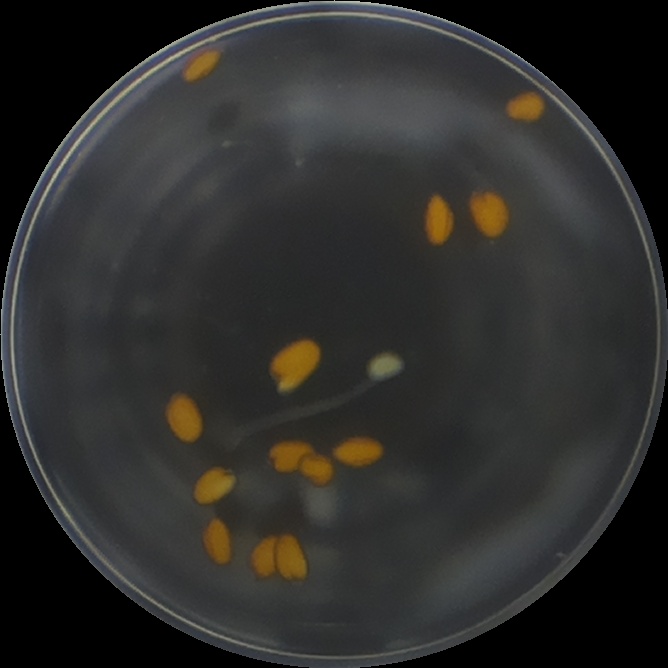

Supplement: Supplementary file 2 — Supplementary Information 2. [file 41598_2020_79115_MOESM2_ESM.zip › PictureOneWell/D5/75_589552]

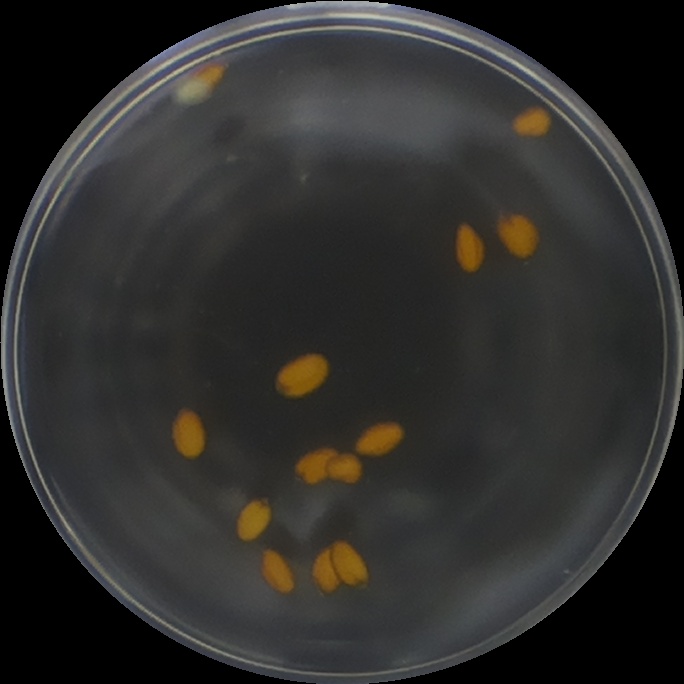

Supplement: Supplementary file 2 — Supplementary Information 2. [file 41598_2020_79115_MOESM2_ESM.zip › PictureOneWell/D5/36_582050]

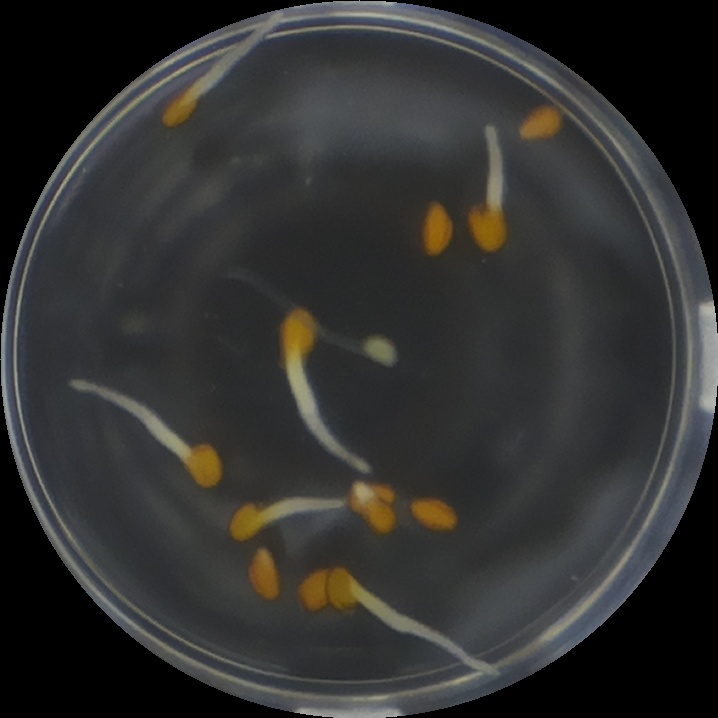

Supplement: Supplementary file 2 — Supplementary Information 2. [file 41598_2020_79115_MOESM2_ESM.zip › PictureOneWell/D5/155_604989]

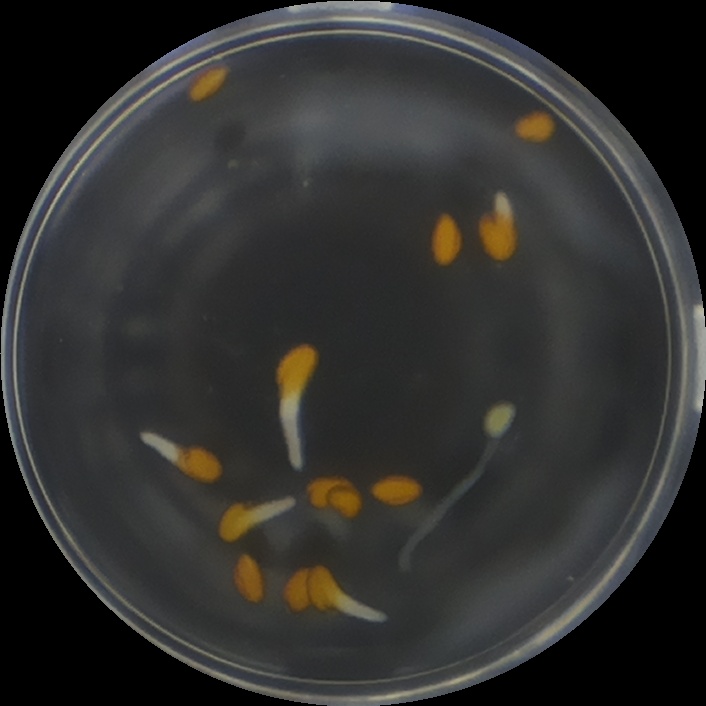

Supplement: Supplementary file 2 — Supplementary Information 2. [file 41598_2020_79115_MOESM2_ESM.zip › PictureOneWell/D5/120_598204]

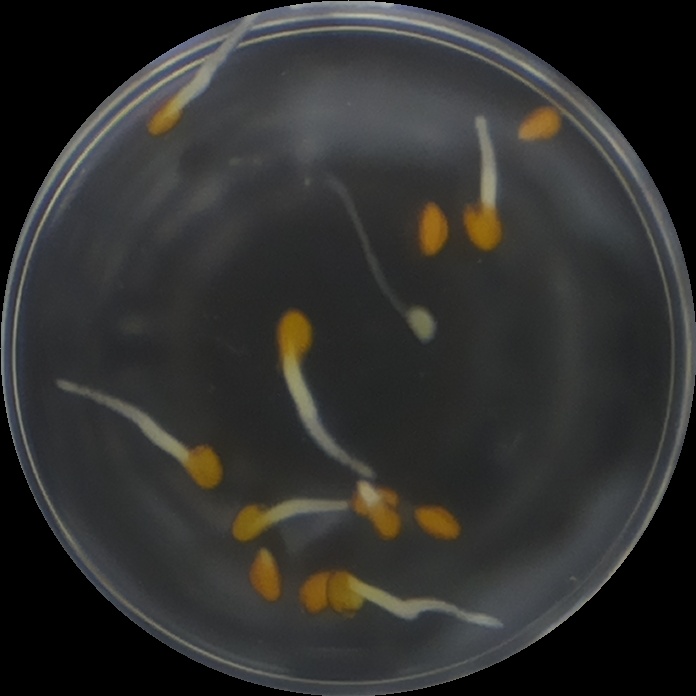

Supplement: Supplementary file 2 — Supplementary Information 2. [file 41598_2020_79115_MOESM2_ESM.zip › PictureOneWell/D5/159_605793]

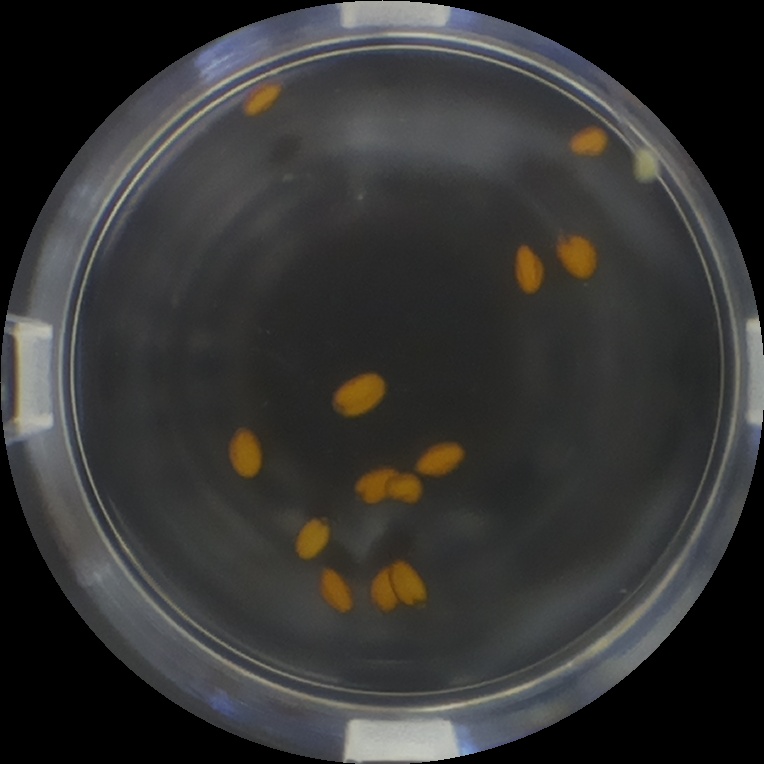

Supplement: Supplementary file 2 — Supplementary Information 2. [file 41598_2020_79115_MOESM2_ESM.zip › PictureOneWell/D5/37_582246]

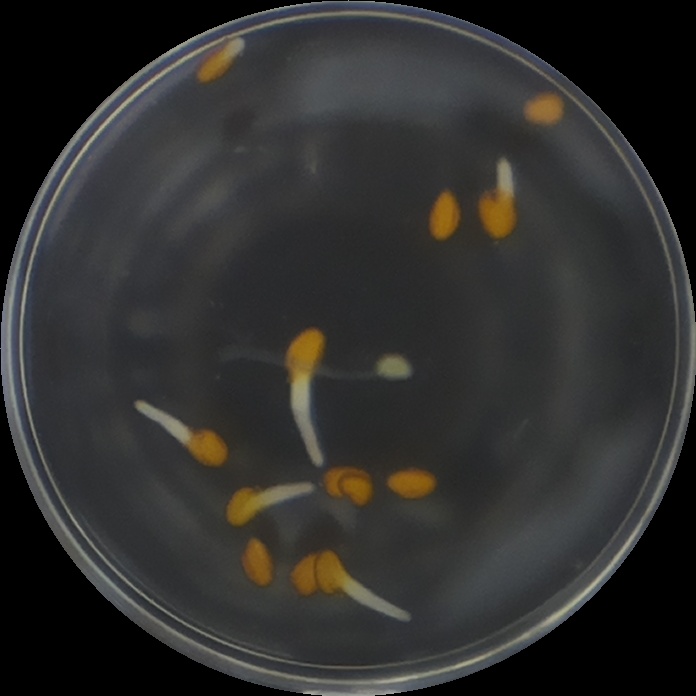

Supplement: Supplementary file 2 — Supplementary Information 2. [file 41598_2020_79115_MOESM2_ESM.zip › PictureOneWell/D5/129_599879]

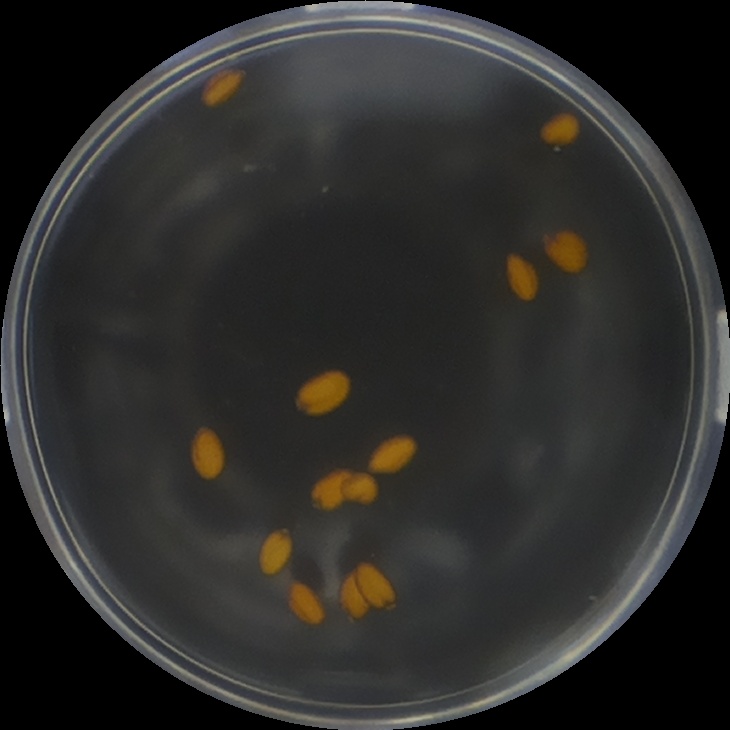

Supplement: Supplementary file 2 — Supplementary Information 2. [file 41598_2020_79115_MOESM2_ESM.zip › PictureOneWell/D5/11_577504]

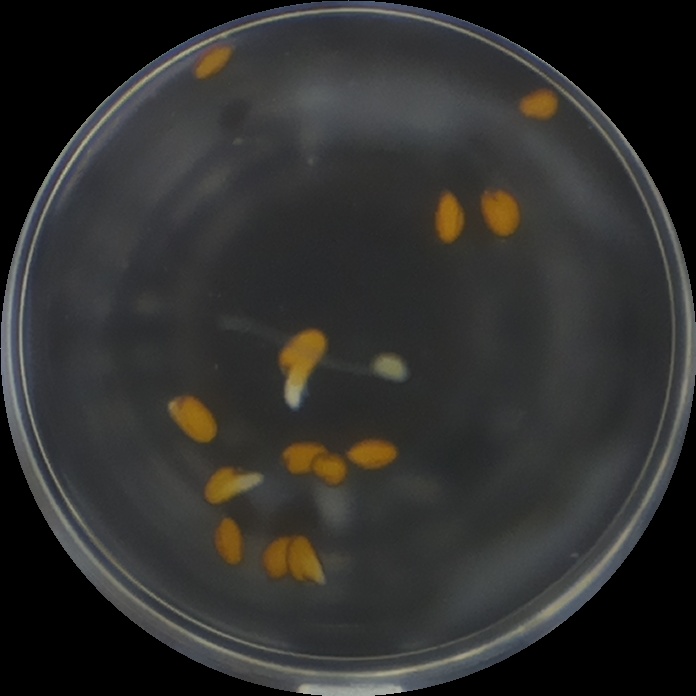

Supplement: Supplementary file 2 — Supplementary Information 2. [file 41598_2020_79115_MOESM2_ESM.zip › PictureOneWell/D5/98_594001]

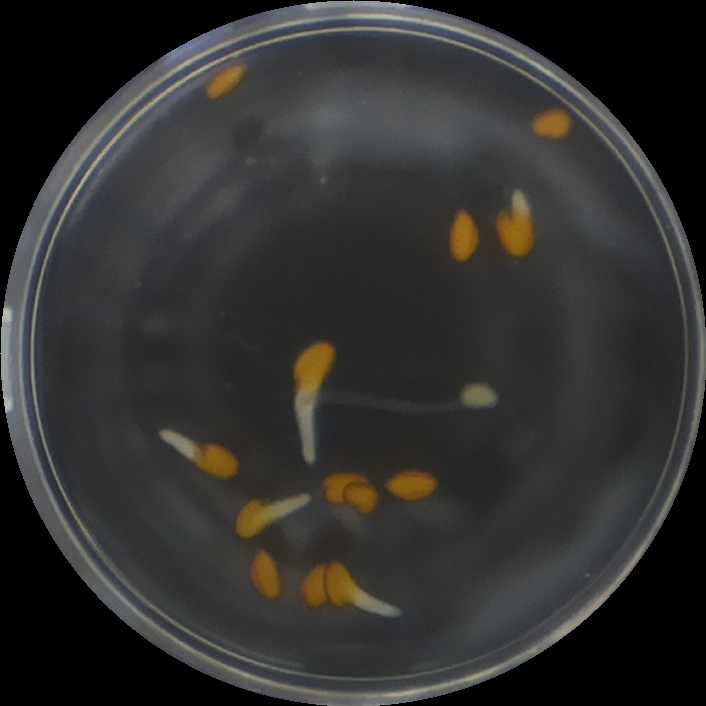

Supplement: Supplementary file 2 — Supplementary Information 2. [file 41598_2020_79115_MOESM2_ESM.zip › PictureOneWell/D5/119_598012]

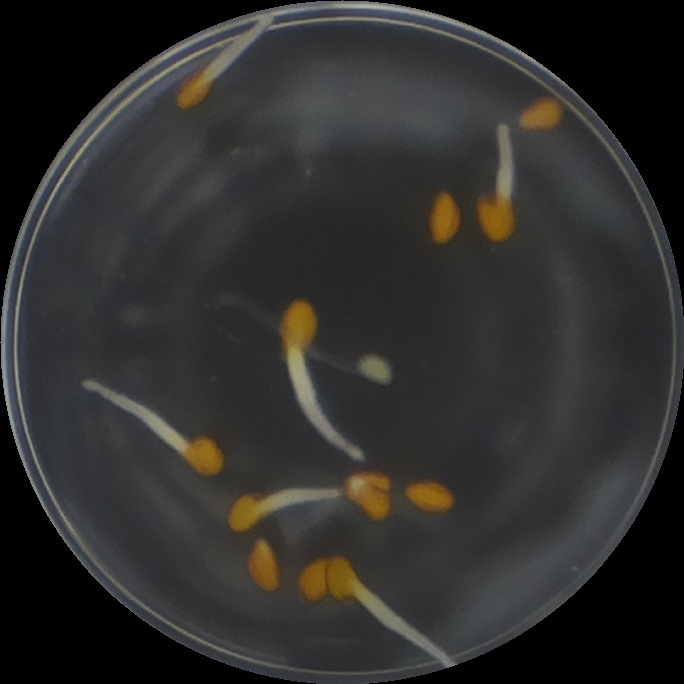

Supplement: Supplementary file 2 — Supplementary Information 2. [file 41598_2020_79115_MOESM2_ESM.zip › PictureOneWell/D5/151_604265]

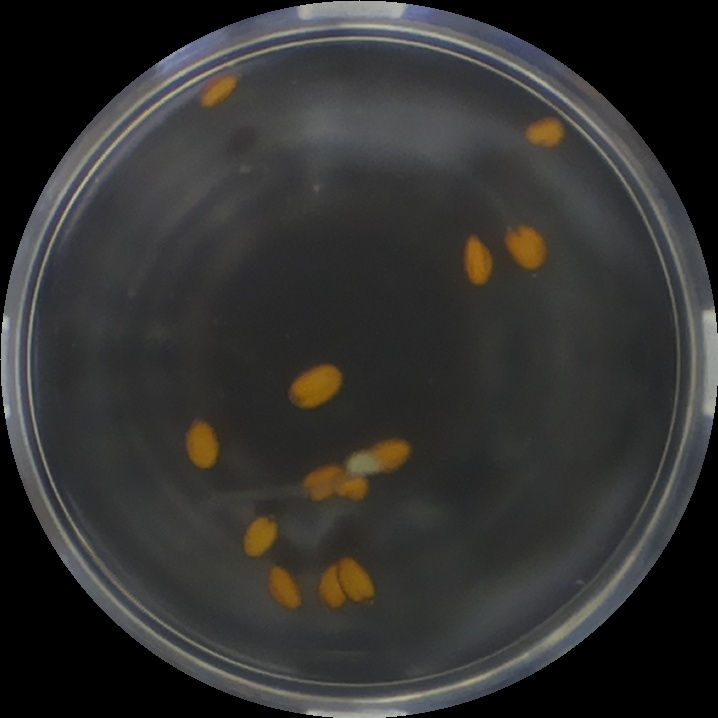

Supplement: Supplementary file 2 — Supplementary Information 2. [file 41598_2020_79115_MOESM2_ESM.zip › PictureOneWell/D5/42_583217]

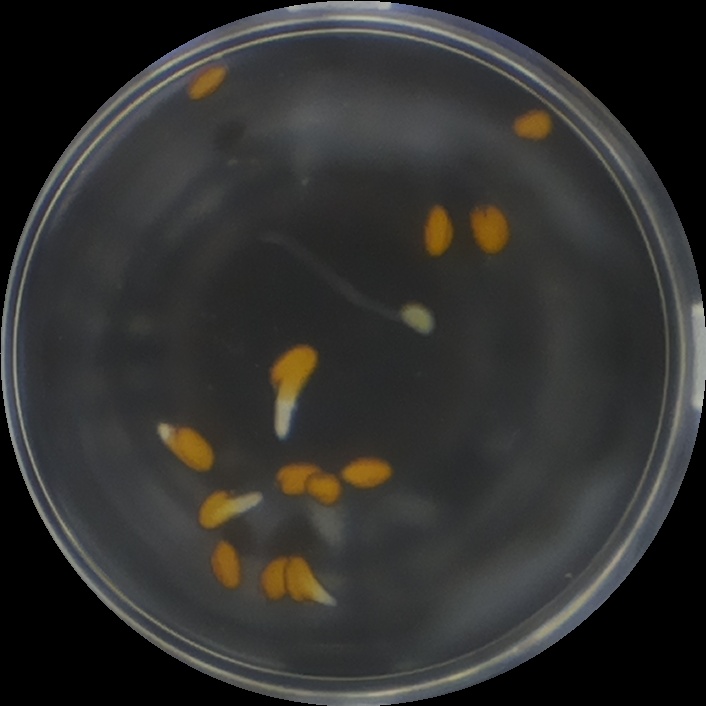

Supplement: Supplementary file 2 — Supplementary Information 2. [file 41598_2020_79115_MOESM2_ESM.zip › PictureOneWell/D5/106_595477]

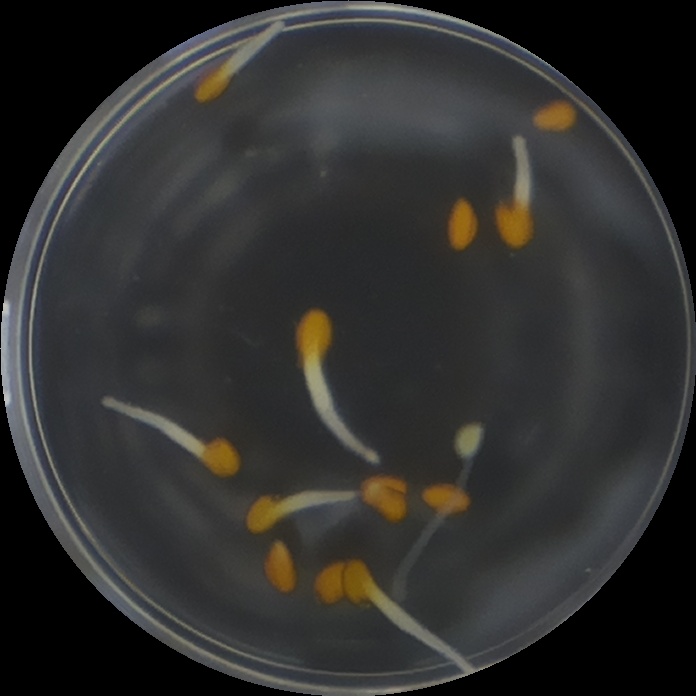

Supplement: Supplementary file 2 — Supplementary Information 2. [file 41598_2020_79115_MOESM2_ESM.zip › PictureOneWell/D5/148_603711]

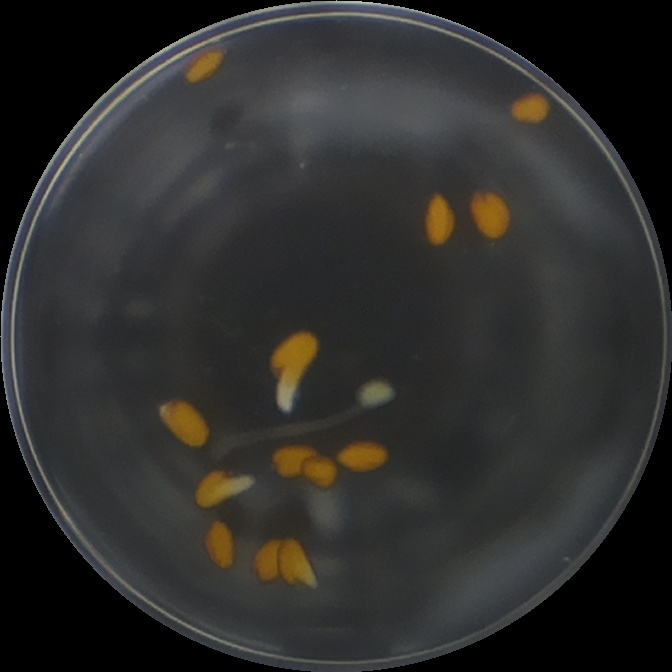

Supplement: Supplementary file 2 — Supplementary Information 2. [file 41598_2020_79115_MOESM2_ESM.zip › PictureOneWell/D5/99_594128]

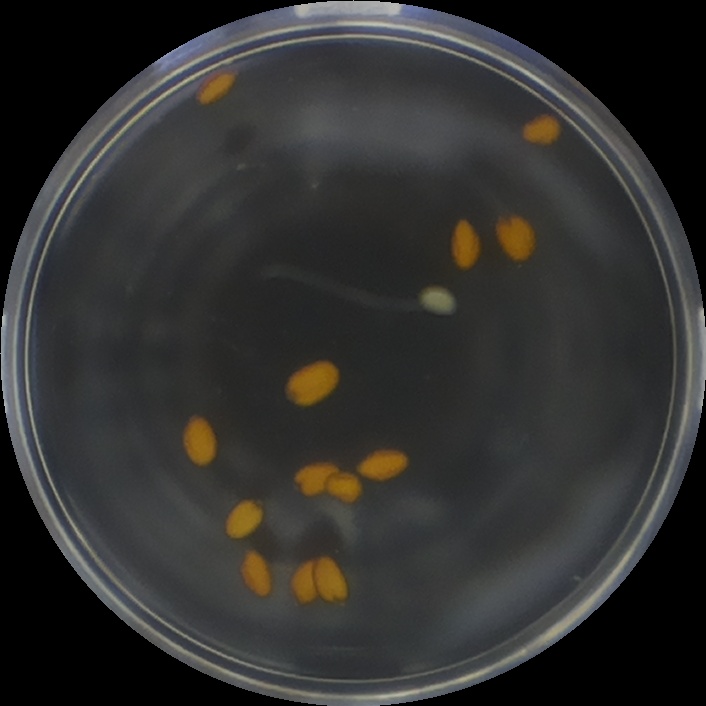

Supplement: Supplementary file 2 — Supplementary Information 2. [file 41598_2020_79115_MOESM2_ESM.zip › PictureOneWell/D5/59_586515]

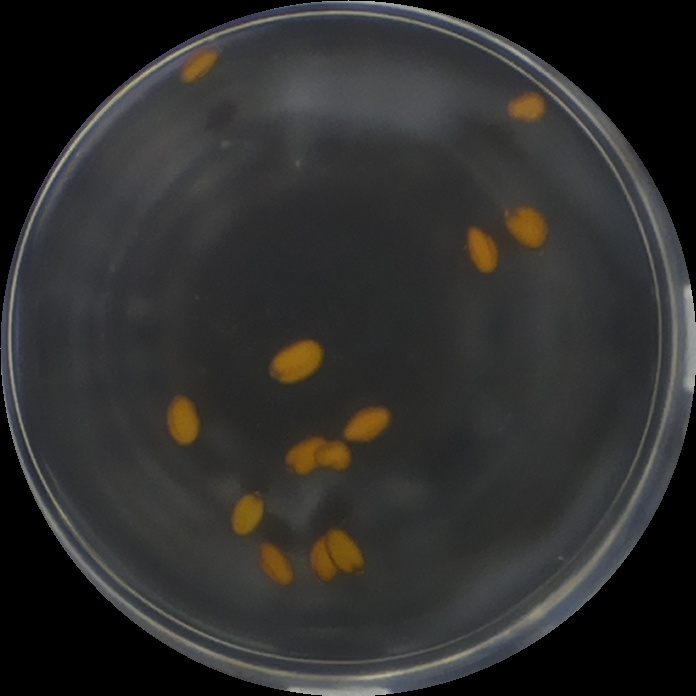

Supplement: Supplementary file 2 — Supplementary Information 2. [file 41598_2020_79115_MOESM2_ESM.zip › PictureOneWell/D5/21_579223]

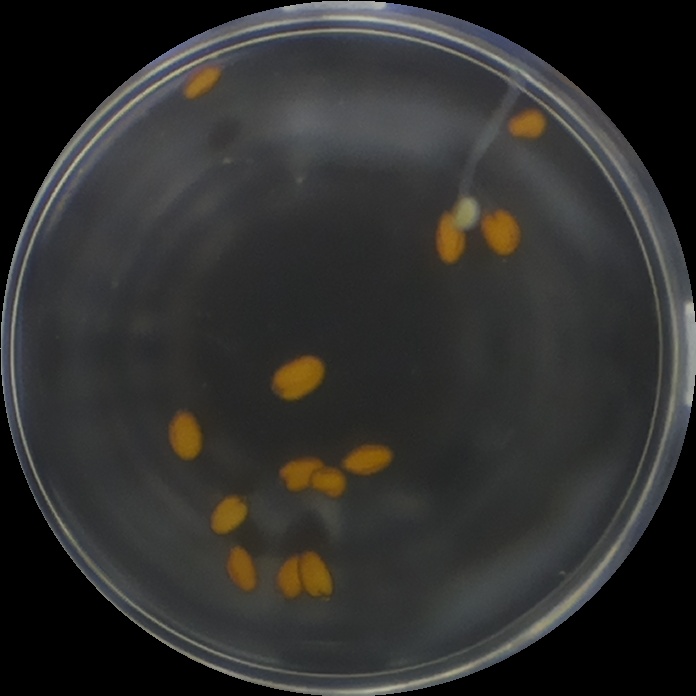

Supplement: Supplementary file 2 — Supplementary Information 2. [file 41598_2020_79115_MOESM2_ESM.zip › PictureOneWell/D5/58_586327]

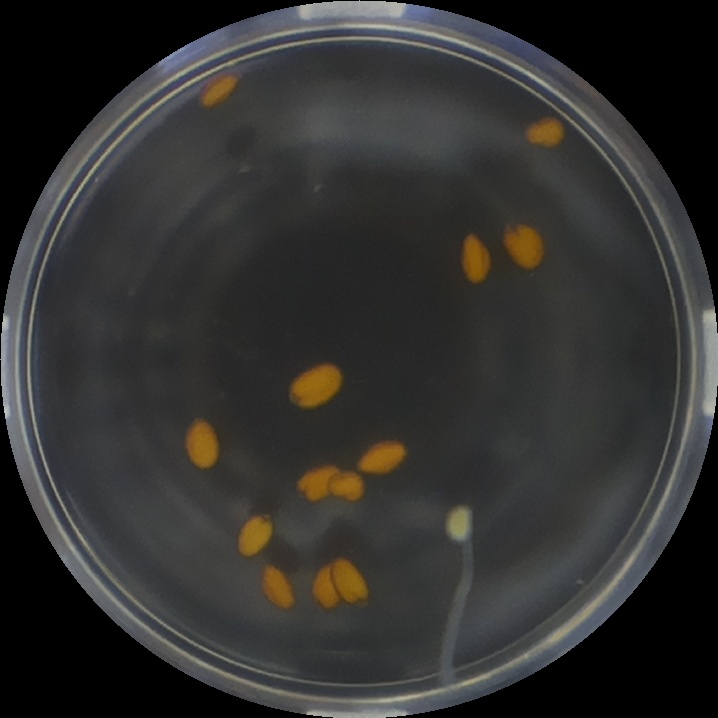

Supplement: Supplementary file 2 — Supplementary Information 2. [file 41598_2020_79115_MOESM2_ESM.zip › PictureOneWell/D5/43_583412]

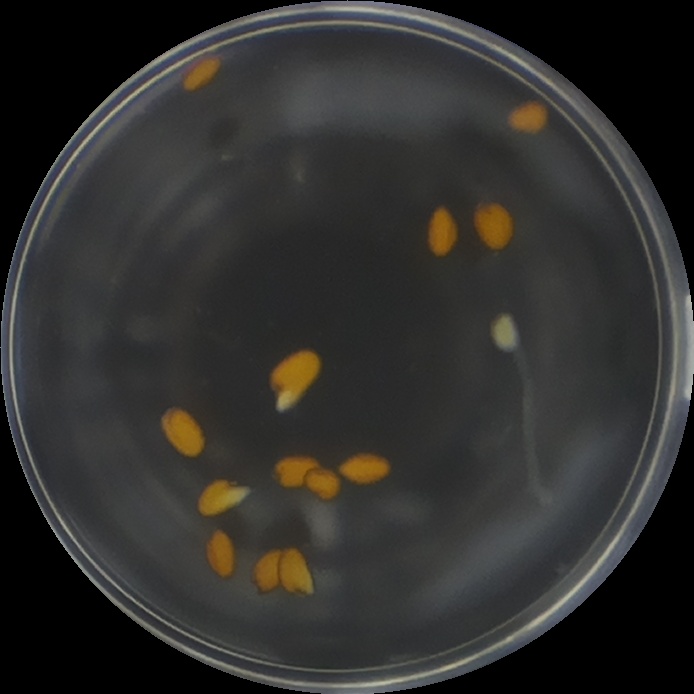

Supplement: Supplementary file 2 — Supplementary Information 2. [file 41598_2020_79115_MOESM2_ESM.zip › PictureOneWell/D5/85_591489]

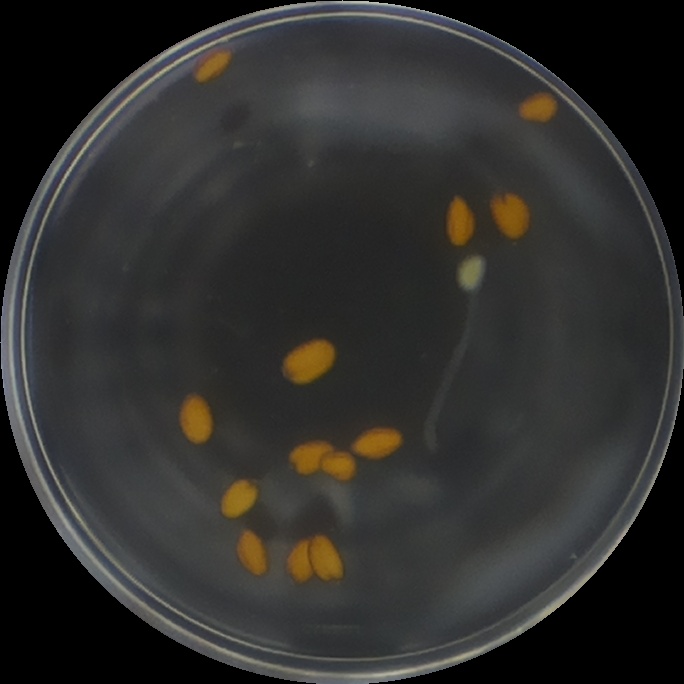

Supplement: Supplementary file 2 — Supplementary Information 2. [file 41598_2020_79115_MOESM2_ESM.zip › PictureOneWell/D5/57_586106]

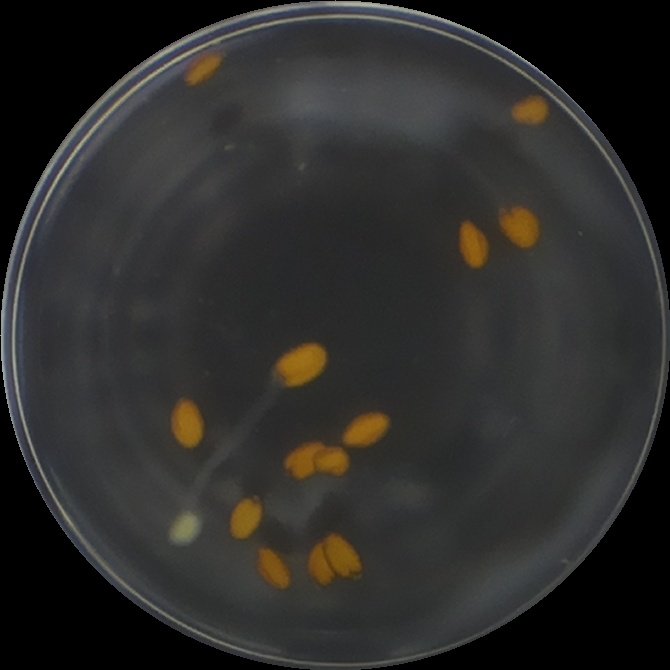

Supplement: Supplementary file 2 — Supplementary Information 2. [file 41598_2020_79115_MOESM2_ESM.zip › PictureOneWell/D5/25_579965]

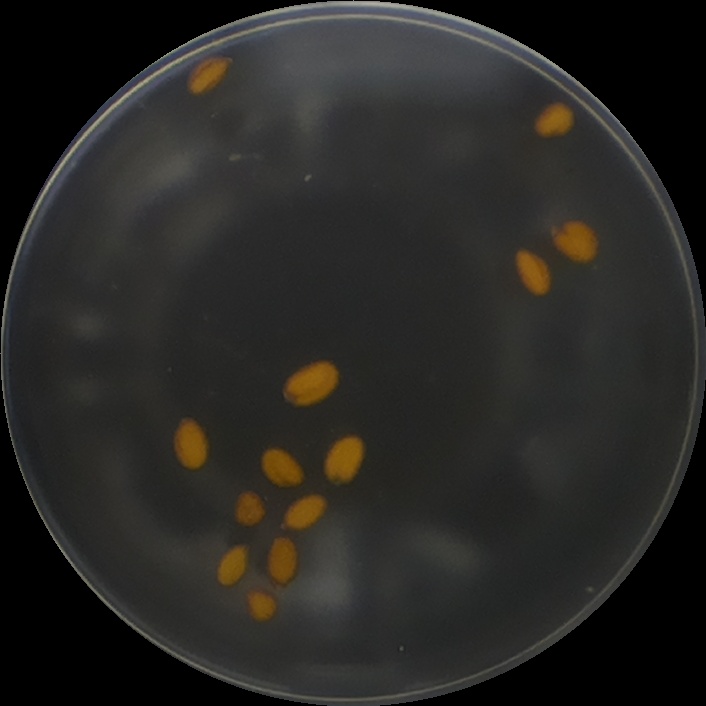

Supplement: Supplementary file 2 — Supplementary Information 2. [file 41598_2020_79115_MOESM2_ESM.zip › PictureOneWell/D5/6_576536]

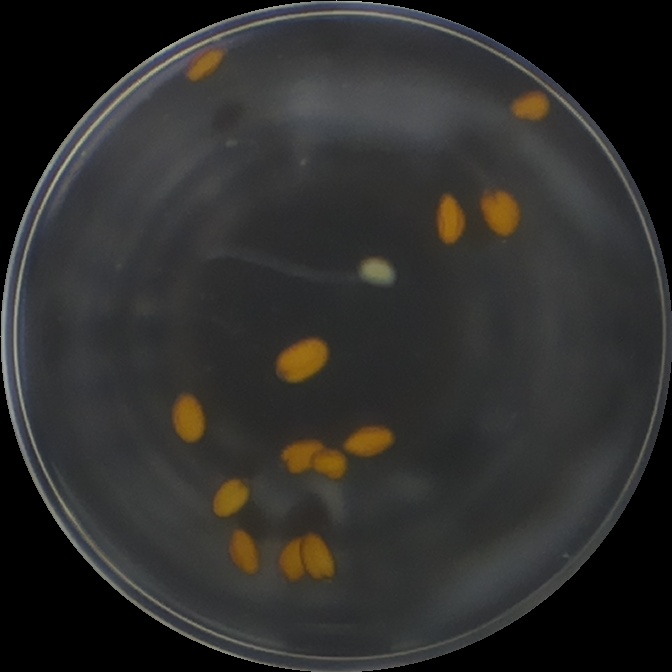

Supplement: Supplementary file 2 — Supplementary Information 2. [file 41598_2020_79115_MOESM2_ESM.zip › PictureOneWell/D5/54_585506]
